# Black Sea picocyanobacteria exploit ancestral traits for energy and chlorophyll production in dark anoxia

**DOI:** 10.1093/ismeco/ycag159

**Published:** 2026-07-17

**Authors:** Maciej Bartosiewicz, Pedro J Cabello-Yeves, Ester M Eckert, Andrea Di Cesare, Richard J Puxty, Anna Przytulska, Helena Osterholz, Anders Meibom, Stephane Escrig, Nina Dzhembekova, Jakob Zopfi, Moritz F Lehmann, Cristiana Callieri

**Affiliations:** Department of Environmental Sciences, University of Basel, CH-4056 Basel, Switzerland; Institute of Geophysics, Polish Academy of Sciences, 01-452 Warsaw, Poland; Evolutionary Genomics Group, Departamento de Producción Vegetal y Microbiología, Universidad Miguel, Hernández, 03550 San Juan de Alicante, Spain; Cavanilles Institute of Biodiversity and Evolutionary Biology, University of Valencia, 46980 Paterna, Valencia, Spain; School of Life Sciences, University of Warwick, Coventry CV4 7AL, United Kingdom; National Research Council, Institute of Water Research, 28922 Verbania, Italy; National Research Council, Institute of Water Research, 28922 Verbania, Italy; School of Life Sciences, University of Warwick, Coventry CV4 7AL, United Kingdom; Institute of Geophysics, Polish Academy of Sciences, 01-452 Warsaw, Poland; Marine Chemistry, Leibniz Institute for Baltic Sea Research Warnemünde, 18119 Rostock, Germany; Laboratory for Biological Geochemistry, École Polytechnique Fédérale de Lausanne (EPFL), CH-1015 Lausanne, Switzerland; Center for Advanced Surface Analysis, Institute of Earth Sciences, University of Lausanne, CH-1015 Lausanne, Switzerland; Laboratory for Biological Geochemistry, École Polytechnique Fédérale de Lausanne (EPFL), CH-1015 Lausanne, Switzerland; Institute of Oceanology, Bulgarian Academy of Sciences, 9000 Varna, Bulgaria; Department of Environmental Sciences, University of Basel, CH-4056 Basel, Switzerland; Department of Environmental Sciences, University of Basel, CH-4056 Basel, Switzerland; National Research Council, Institute of Water Research, 28922 Verbania, Italy

**Keywords:** Black Sea, picocyanobacteria, *Cyanobium,* NanoSIMS, chlorophyll biosynthesis, hydrogen metabolism, fermentation

## Abstract

Picocyanobacteria, typically associated with oxygenated waters, also thrive in oxygen minimum zones (OMZs) and anoxic environments. The extent to which retention of diverse ancestral anaerobic traits facilitate their metabolic flexibility with regards to transitioning between oxic and anoxic habitats is poorly understood. Here, using Black Sea *Cyanobium* (BSA11S), we performed ^13^C-glucose and ^15^N-ammonium incubations coupled with nanoscale ion mass spectrometry (NanoSIMS) and transcriptomics to characterize the growth mode and gene expression during transition from light-to-dark and oxic-to-anoxic conditions. Incubated picocyanobacteria assimilate ^13^C-glucose and ammonium under anoxic-dark conditions, grow slowly without light, and retain the capability to synthesize photosynthetic pigments. These organisms moderately upregulate genes responsible for chlorophyll-*a*/bilin biosynthesis, uptake of C sources, debranching of glycogen storage, and the pentose phosphate pathway for ATP, NADH, and NADPH production, as well as genes involved in NAD+ regeneration, including proton-reduction hydrogen-metabolism coupled with lactic acid fermentation. Our results support the remarkable versatility, plasticity, and potential for mixotrophy in photoautotrophs that allow them to dominate diverse aquatic systems worldwide.

## Introduction

Among all photosynthetic microorganisms, *Prochlorococcus* and *Ca*. Marinosynechococcus/*Synechococcus*-SC5.1 numerically dominate in the ocean’s oxic euphotic waters and contribute ~20%–25% of the global oxygen production [1]. These phototrophs belong to the picocyanobacterial “cluster 5”, which is widespread in freshwater, euryhaline, and marine environments [2–4], and also includes other genera such as, eg, *Cyanobium*-SC5.2 and *Ca*. Juxtasynechococcus-SC5.3 [5]. However, discoveries of novel *Prochlorococcus* strains widespread in oxygen minimum zones (OMZs) [6] have challenged the classic assumption that picocyanobacteria thrive only in well-oxygenated waters. Another recent discovery revealed the presence of *Synechococcus* in the dark anoxic waters of the Black Sea (BS) [7], further challenging the above-mentioned assumption and raising new questions with regards to metabolic versatility and adaptive capacities of these picocyanobacteria [8].

Although classically viewed as oxygenic phototrophs, cyanobacteria can survive without light in environments that are periodically or permanently anoxic [9]. In these habitats, oxygenic photosynthesis may be suppressed mainly due to the absence of light and/or the inhibiting effect of sulfide on photosystem II [10]. Some cyanobacteria, however, can use sulfide as an electron donor to perform anoxygenic photosynthesis [11], while others display alternative modes of energy generation, including photoheterotrophy and mixotrophy [12, 13].

The latter process involves a nutritional mode, in which an organism uses both photosynthesis and consumption of organic matter to acquire energy and nutrients [12]. In dark anoxic conditions, cyanobacteria can perform chemoorganotrophy, taking up glucose, fructose, or sucrose to generate energy through fermentation [9]. Along these lines, with many cyanobacteria aerobically metabolizing endogenously stored glycogen, some ferment the osmoprotectant trehalose to acetate [7]. Under anoxic conditions, cyanobacteria have also been reported to get rid of excess reducing power by H_2_ generation through proton reduction. All of the enzymatic components (Hox bidirectional hydrogenases) used to produce hydrogen are ubiquitous in cyanobacteria [14, 15] and allow them to grow under oxygen-free conditions. In this context, recent work on SC5.2 picocyanobacteria (*Cyanobium*) isolates from the deep sulfidic waters of the BS revealed not only survival in oxygen-free conditions but also chlorophyll accumulation [7, 8] in the dark anoxic waters. Crucial insights into the ecological and molecular mechanisms supporting picocyanobacterial growth under such adverse conditions are, however, still missing.

To address this knowledge gap, we performed incubation experiments with the BS picocyanobacterium (*Cyanobium* strain BSA11S) under different light and oxygen conditions. We tested whether this strain was able to do the following: (i) survive under dark/anoxic conditions, (ii) produce chlorophyll in the dark, (iii) use glucose and ammonium as metabolic substrates under dark/anoxic conditions, and (iv) use other reductant sources coupled to hydrogen production. We assessed the effect of potential fermentation and hydrogen metabolism on photosynthetic pigment accumulation and followed gene expression and DOM excretions under different oxygen and light regimens to elucidate the metabolic pathways that likely support the survival of picocyanobacteria in euxinic waters.

## Materials and methods

### Experimental design, cell counting, and chlorophyll measurements

**Figure 1:**
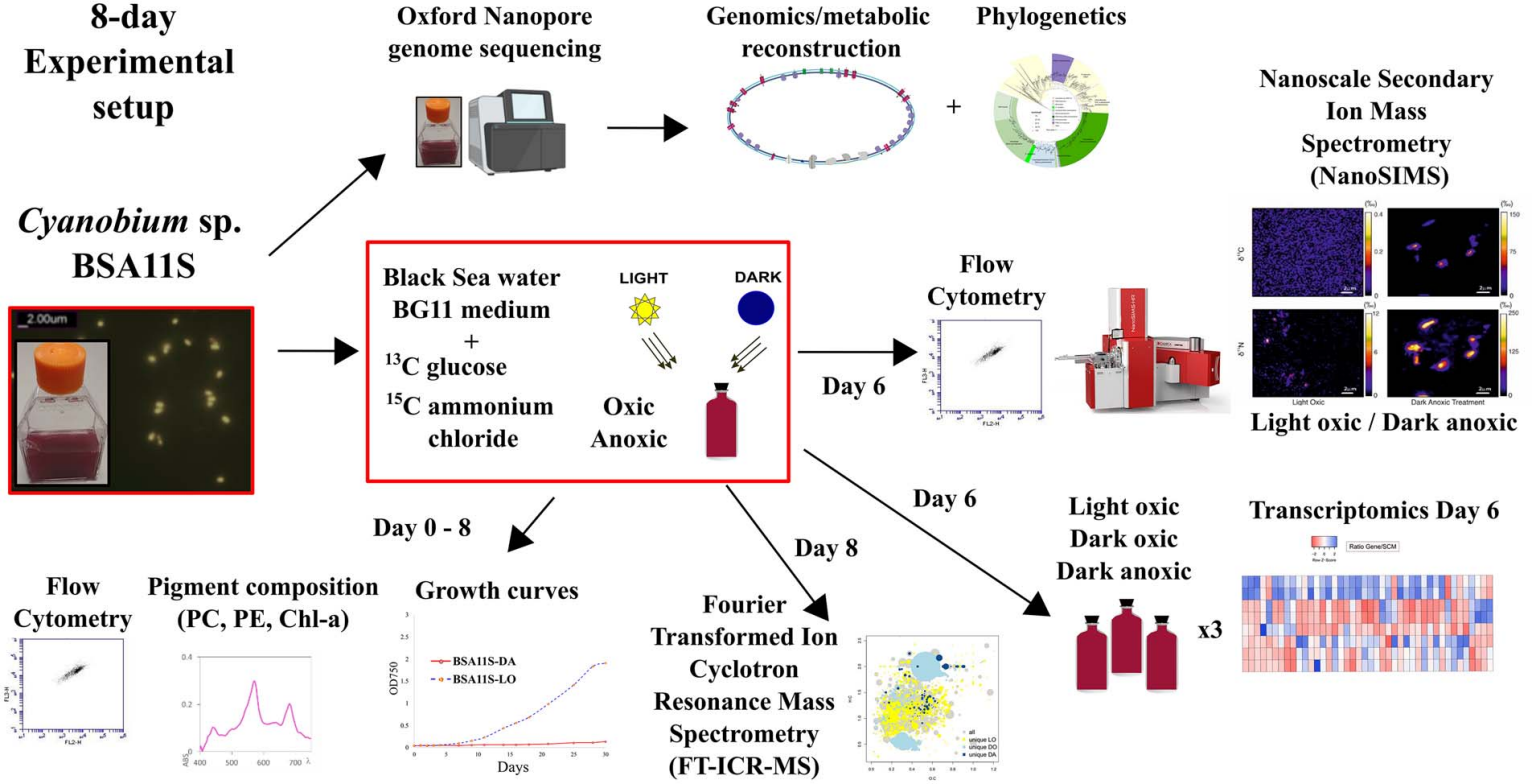
Detailed experimental approach used in all experiments with the Black Sea picocyanobacteria. The different techniques used in our approach and the time points (days) are displayed accordingly.

For our experiments we used the BS picocyanobacterium *Cyanobium* (strain BSA11S, subcluster 5.2) that was isolated from the western part of the BS at 6.5 m of depth, as previously described [7, 8]. Flow cytometry and cell sorting allowed us to obtain a pure, monoclonal, and nearly axenic culture with a single *Erythrobacter*-like associated heterotroph, which represented only <5% of all cells. This procedure was the beginning of incubations subjected to dark/anoxic (DA), dark/oxic (DO), and light/oxic (LO, control) conditions (Fig. 1 provides an overview of the experimental system). Incubations were performed in modified BG11 medium [16, 17] prepared with 0.2-μm-filtered BS water (from 750 m). For the DA treatment, the autoclaved medium was prepurged with N2 gas for >1 hour. The medium was then enriched with ^13^C-glucose (100 nM, final concentration) and ^15^N ammonium chloride (500 nM) and inoculated with strain BSA11S at a final density of 1.6 × 10^6^ cells mL^−1^.

For the DA and DO treatments, 120 mL serum vials were first covered with nontransparent black tape and then placed in a dark incubator. For the LO treatment, the inoculated vials were kept at 10 μmol photons m^−2^ s^−1^. All vials (30 per treatment, 90 in total) were incubated without headspace on rotating tables in thermostatic chambers (ThermoScientific) at 20°C. Total incubation time was 8 days, with sampling of 5 replicates, respectively, every other day (ie D0, D2, D4, D6, D8) for picocyanobacterial cell counts and chlorophyll determination. Additional vials for each treatment were equipped with a dissolved oxygen sensor (PyroScience) to assure persistent anoxia in dark anoxic incubations. Samples for NanoSIMS stable-isotope imaging and transcriptome analyses (after transferring the remaining unopened vials into an anoxic chamber) were collected at D6. These 2 complementary methods allowed us to verify that active cells observed with the microscope and flow cytometry were picocyanobacteria and not any other associated bacteria. In particular, we were interested in verifying their activity under the most difficult conditions for photosynthetic cells: that is, in the dark and in the absence of oxygen (DA).

Biomass for molecular work in the DO and DA treatments was collected using a light-shielded setup, and DA-treatment samples were processed in an anoxic glove box purged continuously with N_2_. Picocyanobacterial cells were counted using a flow cytometer (Accuri C6, Becton Dickinson, Oxford, UK), equipped with a 20 mW 488 nm Solid State Blue Laser and a 14.7 mW 640 nm Diode Red Laser. Cells were quantified using light-scattering signals (forward and side light scatter referred to as FSC-H and SSC-H, respectively), orange fluorescence (FL2-H channel = 585/40 nm), and red fluorescence (FL3-H channel >670 nm and FL4-H channel 675/25) as in previous work [7]. Samples for chlorophyll concentration analyses were filtered on glass fiber filters (GF/F) and stored at −20°C until further processing. Concentrations of chlorophyll *a* were assessed spectrophotometrically, following the extraction of the filtered biomass with acetone [18]. An additional experiment to monitor picocyanobacterial growth during 21 days was conducted (see Fig. S1). Briefly, we used light and dark controls with and without 0.1% glucose (5 mM) under oxic conditions in triplicates and measured absorbance at 750 nm using a Bibby Scientific™ Jenway™ 7305 spectrophotometer. Net growth (μ) was calculated as ln(ODt2)-ln(ODt1)/t2-t1, with t indicating the days.

### Stable isotope probing

For analysis by Nanoscale Secondary Ion Mass Spectrometry (NanoSIMS), flow-cytometry-sorted picocyanobacterial cells (in paraformaldehyde solution at 1.6% v/v; Day 6,) were directly collected (as a single layer) on laser-marked polycarbonate filters (GTTP, Millipore). This innovative method allows us to establish cell-level activities (for NanoSIMS images see also Supplementary NanoSIMS Images file). Filters were first rinsed with freshwater BG11 medium to remove salt then dried and mounted on a 5-mm round aluminum support with a double-sided adhesive copper tape. Epifluorescence microscopy land laser markings were used to locate specific regions of interest (ROIs) containing pigmented cells. Filters were subsequently sputter coated with a layer of gold film (20-nm thickness) before introduction into a NanoSIMS 50 L at the Laboratory for Biological Geochemistry of the EPFL Lausanne. The selected ROIs areas were presputtered with a Cs^+^ primary ion beam of 15 pA for 5 minutes to remove surface contamination, implant Cs^+^ ions on the sample, and achieve a stable ion emission rate. A primary Cs^+^ ion beam with a current between 1 and 1.2 pA and a beam diameter of ∼150 nm was rastered across the cells for analysis with a dwell time of 5 ms per pixel. Secondary ion images for ^12^C^12^C^−^, ^12^C^13^C^−^, ^12^C^14^N^−^, ^12^C^15^N^−^, and ^32^S^−^ were simultaneously recorded at a mass resolution of around 9000 (Cameca definition) from areas of typically 20 × 20 μm^2^ covered by 256 × 256 pixels [19, 20]. The rastering was repeated 4 times over the same area to improve the precision of the quantification of isotopic enrichment. Images were processed using L’IMAGE software (developed by Pr. L. Nittler, Arizona State University, USA). Statistical analyses were performed using the Statistica package 2019. Isotopic enrichments are presented either as absolute isotope ratios or in δ notation, ie, in parts-per-thousand (‰) relative to a control sample of natural-abundance isotopic composition prepared and analyzed in an identical manner (yielding ^12^C^13^C/^12^C_2_ equals ~0.02116 and ^12^C^15^N/^12^C^14^N = 0.003737 as references for the δ-values). Statistical comparison of treatments was done using a Wilcoxon rank sum.

### Fourier transformed ion cyclotron resonance mass spectrometry

For each treatment (DA, DO, LO), parallel incubations were conducted in 500-mL serum bottles under the same conditions as described above for the 120-mL vials. At D_8_ dissolved organic matter (DOM) was collected for analysis by Fourier Transform Ion Cyclotron Resonance Mass Spectrometry (FT-ICR-MS) [21]. Specifically, experimental medium was filtered, acidified to pH 2, and passed through a solid-phase extraction column (Bond Elut PPL cartridge, 1 g, Agilent) [21]. Methanol extracts were diluted with ultrapure water (1:1 ratio, v:v), to yield a final concentration of ~4–5 mg C L^−1^. Duplicate mass spectra for the 3 samples were obtained on a 15 T FT-ICR-MS (Bruker Daltonics), coupled to an electron spray ionization (ESI) ion source (Bruker Apollo II), with a capillary voltage of 4.5 kV in negative ion mode. Then 200 broadband scans using 8 megaword datasets in a mass range of 92–2000 Da were co-added, and duplicate analyses were performed for each sample. Molecular formulas were assigned using ICBM-Ocean [22] in the range C_1–50_H_1–200_O_1–50_ N_0–4_S_0-2_P_0–1_, with a tolerance of 0.5 ppm in the mass range from 100 to 700 Da, and excluding isotope-ratio mismatches below a signal-to-MDL (method detection limit) ratio of 6. Singlet molecular formulas were excluded. The relative intensities of all peaks were then normalized to the sum of intensities per sample. From this dataset (Table S1), we calculated the intensity-weighted elemental ratios, compound group contribution, a modified aromaticity index (AImod) [23], and the nominal oxidation state of carbon (NOSC = 4-((4 • C_count+H_count – 3 • N_count-2 • O_count – 2 • S_count) C_count) [24].

### DNA extraction, nanopore sequencing, and genome assembly

Genomic DNA of *Cyanobium* sp. BSA11S was extracted using a CTAB-lysis buffer as described elsewhere [3]. Approximately 5 μg purified DNA was sequenced with an Oxford Nanopore MinION using a FLO-MIN106 flow cell and a SQK-LSK109 sequencing kit. Sequencing provided 211 226 reads with an average read size of 2011 bp for a total sequenced 424 780 710 bp. Only reads >1 kb were used for genome assembly (Flye v.2.9-b1778 with parameters: –nano-hq –m 1000 –t 64 –o) [25] resulting in a closed genome for *Cyanobium* sp. BSA11S of 3 073 547 bp and a total GC% of 65.48, with 3465 CDS (Coding Sequence), 2 ribosomal. operons, and 46 tRNAs.

### Gene prediction, annotation, and individual gene/protein phylogenetic analysis

The complete genome of *Cyanobium* sp. BSA11S was annotated using the following pipeline: Gene prediction was assessed using Prodigal v2.6.3 [26], and each CDS was annotated with Diamond [27] and blastx v2.0.6.144 ran against the non-redundant KEGG [28], SEED [29], COG [30] and TIGRFAMs [31] databases, to obtain the latest and most robust annotation. The tRNA and rRNA genes were detected with tRNAscan-SE 2.0.5 [32] and ssu-align [33], respectively. Details of all gene annotations are shown in Supplementary Dataset 1. To construct a protein phylogenetic tree for lactate dehydrogenases and the alpha subunit HoxH of the bidirectional hydrogenase, individual protein sequences were downloaded from the NCBI-nr and aligned with MAFFT v7.453 [34]. Aligned sequences were then processed with IQ-TREE 1.6.12 [35] (parameters -bb 1000 -nt AUTO -alrt 1000) to determine the optimal model for each protein type (LG + F + R10 for hydrogenase HoxH and WAG+F + R8 for lactate dehydrogenases) and construct the final phylogeny.

### Transcriptomics analysis

Transcriptomic analyses were conducted for triplicate samples, collected at D_6_ as 40-mL aliquots, concentrated at 13 000 g for 15 min, snap frozen in liquid N_2_, and stored at −80°C. RNA was extracted using the MasterPure™ Complete DNA and RNA Purification Kit (Lucigen, Middleton, WI). The RNA purification protocol was followed after resuspension of the frozen pellets in 600 μL tissue- and cell-lysis solution containing 2 μL proteinase K. After DNA removal, RNA was resuspended in 35 μL TE Buffer. A Zymo-Seq RiboFree total RNA library preparation kit (Zymo Research Corp., Irvine, CA) was used for library preparation (library type: fr-firststrand, fr-secondstrand). The quantity and quality of extracted RNA was determined with an Agilent 2100 Bioanalyzer RNA assay or Caliper (PerkinElmer, Waltham, MA). Final libraries were checked using a Qubit 2.0 fluorometer (Invitrogen, Carlsbad, CA), Agilent Bioanalyzer DNA assay, or Caliper assays (PerkinElmer, Waltham, MA), and sequenced in single-end 150-bp mode on a NextSeq 500 system (Illumina, San Diego, CA).

Sequenced transcriptomes provided between 19 and 49 × 10^6^ reads for each treatment (Table S2), yielding 1–4.9 Gb at an average length of 112–140 bp. Raw reads were trimmed with Trimmomatic [36] v0.3934 with SE –threads 64 –phred33 and LEADING:3 TRAILING:3 SLIDINGWINDOW:4:15 MINLEN:36 parameters. First, we estimated the similarity of each transcriptome dataset using SIMKA [37] with –kmer-size 21, −abundance-min 2 parameters. Using the same software, we performed Bray–Curtis dissimilarity presence/absence and abundance clustering (hclust) analyses and used PCA to evaluate the similarity between LO, DA, and DO treatments. Then, the individual trimmed reads for each treatment were mapped to each CDS from the closed genome using bowtie2 [38] with — sensitive-local —no-unal —no-mixed —no-discordant —threads 64 parameters. This output (.sam files) was processed with SAMtools [39], using view (to produce .bam files); sort, index; and idxstats to obtain the total read counts mapped against each CDS.

Read counts were normalized in R using the *DESeq2* package (v 1.36.0) [40], and differential expression of the genes was evaluated by estimation of size factors and dispersal, as well as the Negative Binomial GLM fitting and Wald statistics. The dataset was evaluated in terms of similarity (Euclidean distance) with the function normTransform and plotted with the function pheatmap, and the influence of specific treatments (ie, light and oxygen) on the similarity of samples was evaluated using PERMANOVA (*vegan* v. 2.6.2) [41]. Additional analyses were conducted to compare LO and DA treatments with regard to (a) the complete genome and (b) focusing on a subset of genes defined as key genes for the targeted metabolisms (see Supplementary Dataset 2 for up- and down-regulated genes). The log2 of the fold changes of all genes and of key genes were plotted in a violin diagram and a barplot using *ggplot2* [42] (see Fig. 3, Left). The R package *ashr* was used to estimate false discovery rates (FDR) [43]. All scripts used for transcriptome analysis and generating plots are located in the Github repository: https://github.com/EsterME/LO_DA_transcriptome.

### Data accessibility

The complete genome of *Cyanobium* sp. BSA11S and raw sequences of transcriptomes have been deposited at NCBI under Bioproject PRJNA966923.

## Results

### Growth rate and pigmentation

**Figure 2:**
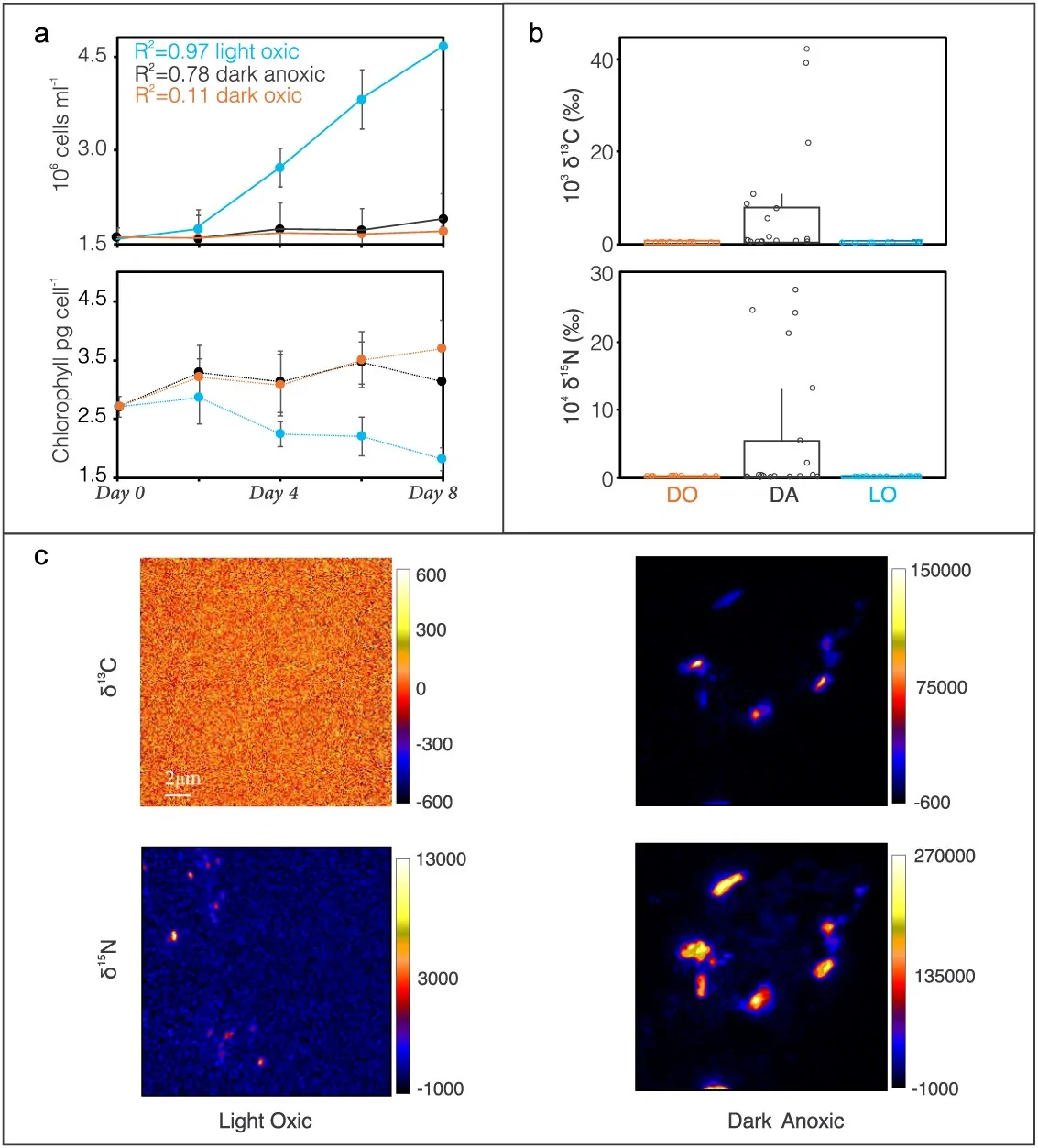
(a) Picocyanobacterial abundance (upper panel) and pigmentation (lower panel) during incubations under light oxic (LO), dark oxic (DO), and dark anoxic (DA) conditions; T = day. (b) R^2^ indicates significant change in time. NanoSIMS measurements of individual cells (open circles, *n* = 30 for DA and *n* = 24 for LO + DO) incubated with ^13^C-glucose and ^15^N-ammonium chloride. Boxes represent box-and-whisker plots of cellular isotope enrichments under the different light and redox conditions (interquartile range, and range). Consistent with autotrophic vs. heterotrophic metabolism, cells incubated under LO and LA conditions are significantly different from enriched cells under DA conditions. (C) Examples of NanoSIMS maps showing ^13^C and ^15^N enrichments of individual picocyanobacterial cells isolated from LO and DA conditions, respectively.

Emulating habitat complexity in the BS, we followed cell number and pigmentation in dark/anoxic (DA), dark/oxic (DO), and light/oxic (LO) treatments. Throughout all experiments, picocyanobacteria maintained a positive growth rate (Fig. 2a). Cells multiplied most rapidly in the LO treatment (specific growth rate = 0.13 d^−1^), and the cell density during incubation increased from 1.6 × 10^6^ (Day 0) to 4.7 × 10^6^ cells mL^−1^ (Day 8). Picocyanobacteria in the DO treatment grew from 1.6 × 10^6^ (Day 0) to a final density of 1.7 × 10^6^ cells mL^−1^ (Day 8), with specific growth rate at only 0.01 d^−1^. Cells in DA also survived and multiplied from 1.6 × 10^6^ (Day 0) to a final density of 1.9 × 10^6^ cells mL^−1^ (Day 8, maximum between replicates = 2.2 × 10^6^ cells mL^−1^), yet again at a much slower rate (0.02 d^−1^) than in the LO treatment. *Cyanobium* sp. BSA11S remained pigmented during all experiments, irrespective of the treatment (Fig. 2a). Total chlorophyll *a* content increased between D_0_ and D_8_, from 2.7 (±0.18) pg cell^−1^ to 3.7 (±0.46) pg cell^−1^ in DO, and to 3.2 (±0.31) pg cell^−1^ in DA. In contrast, the per-cell chlorophyll content decreased in the LO treatment to 1.8 (±0.18) pg cell^−1^. An additional 21-day growth curve was obtained under oxic conditions including light without glucose, light supplemented with glucose, dark with glucose, and dark without glucose (see Fig. S1). The resulting net growth rates reached 0.1478 d^−1^ in light without glucose followed by light with glucose treatment (μglobal = 0.1472 d^−1^). Then, the dark treatment supplemented with glucose showed growth rates at 0.0116 d^−1^ that were 5 times greater than those for dark without glucose, matching the results from our main 8-day experiment (Fig. 2a).

### Picocyanobacterial growth mode

Visualization of individual cells grown in the presence of ^13^C–glucose by NanoSIMS showed that *Cyanobium* sp. BSA11S exposed to DA conditions had a ^13^C/^12^C ratio an order of magnitude higher than under LO/DO conditions (Fig. 2b), ie, an average δ^13^C of 6578 ± 2678.6‰ (*n* = 30) in DA conditions compared to 32 ± 187‰ (largely unlabeled/sporadically enriched; AVG ± SE, *n* = 24) under DO and LO conditions (Wilcoxon tank sum exact test: W = 56, p = 5.5 × 10^−6^, Fig. 2b, 2c). A similar pattern was observed for the ^15^N/^14^N ratios, with much greater ^15^N enrichment in samples incubated with ^15^N-ammonium under DA conditions (mean δ^15^N = 6.3 × 10^4^), compared to DO (mean δ^15^N = 216) or LO conditions (mean δ^15^N = 103) Fig. 2b, Wilcoxon tank sum exact test: W = 12, p = 5 × 10^−10^. The apparently lower ^15^N enrichment in LO and DO treatments can reflect an isotopic dilution effect: cells in LO grew ~6× faster, distributing the same ^15^N tracer pool (at 500 nM) across a much larger biomass, reduced per-cell enrichment below NanoSIMS detection sensitivity. Additionally, cyanobacteria could have consumed ammonium faster in LO, since there is a preference for its use over nitrate via hierarchical nitrogen control.

Molecular characterization of *Cyanobium* sp. BSA11S exudates revealed 3723 molecular formulas (MFs), of which 1641 were present in all samples. Most of the MFs that were treatment specific (Fig. S2) were assigned to LO (9% of the total), compared to DA and DO (0.8% and 4%, respectively). Unique exudates contained more phosphorus and sulfur than the core DOM pool, and in LO and DA they were also enriched in N compared to C. The few unique exudates in DA had, on average, high molecular masses and were characterized by high H/C ratios, pointing to release of large aliphatic compounds. Overall, carbohydrate-type exudates were relatively more abundant (as total intensity) in the DA treatment (0.47%) compared to the DO and LO treatments (LO: 0.11%, DO: 0.17%, Table S1 and S3). The nominal oxidation state of carbon, indicative of DOM reactivity, in *Cyanobium* sp. BSA11S exudates was lowest in DO (−0.53 ± 0.01), followed by DA (−0.31 ± 0.001) and LO (−0.2 ± 0.02; Table S1).

### Genomic features - carbohydrate and anaerobic metabolism of *Cyanobium* sp. BSA11S

**Figure 3:**
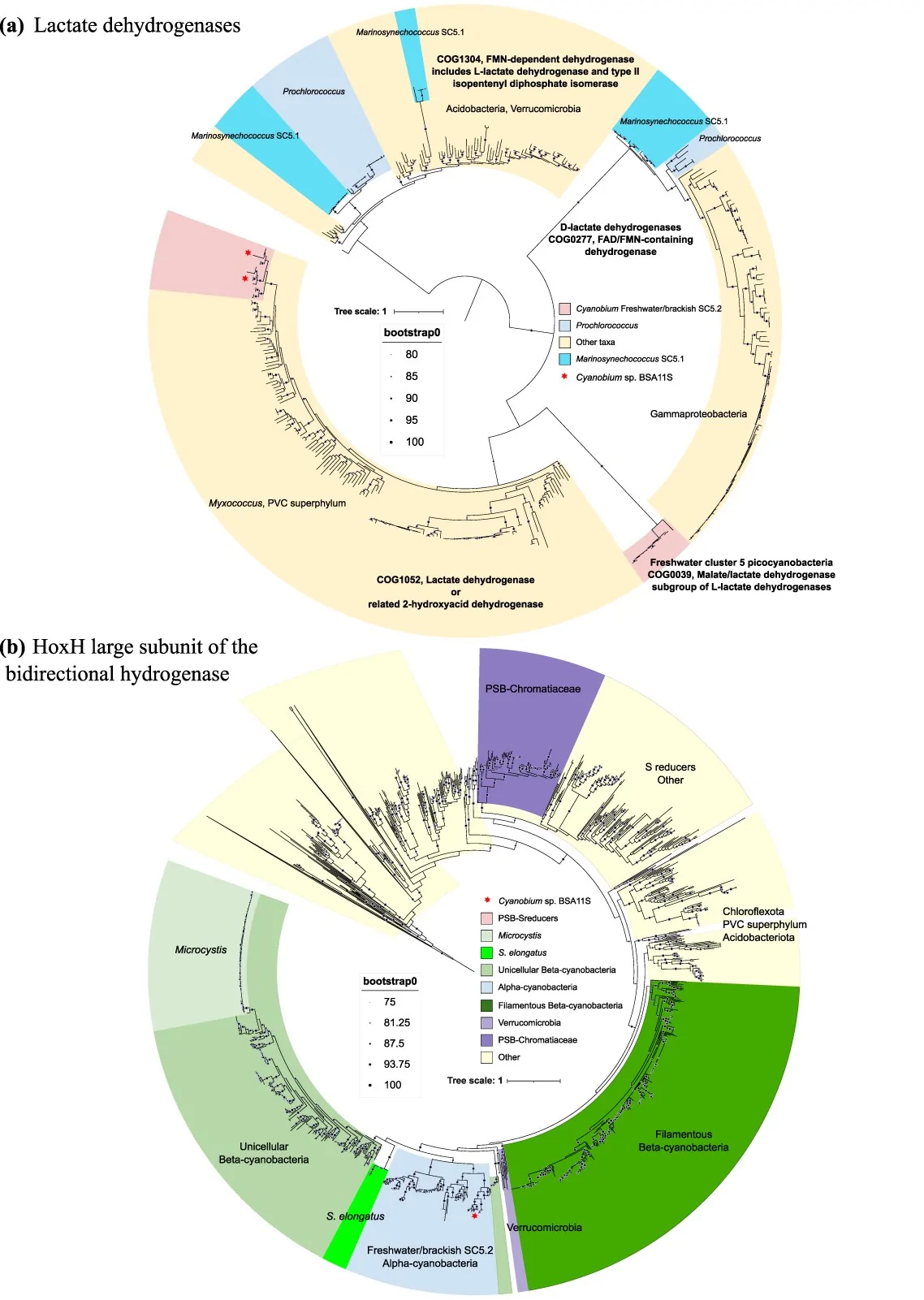
Mid-point rooted (a) D/L/FMN-type lactate dehydrogenases and (b) HoxH large subunit of the bidirectional hydrogenase phylogenies showing picocyanobacteria and closest bacterial phyla featuring lactic fermentation/hydrogenase enzymes. Only bootstraps >80 are shown for the different branches. The Black Sea strain BSA11S/BSF8S lactate dehydrogenases/hydrogenase are colored in red and coded with a red star. Different taxa are also color coded. The phylogenetic tree was produced with IQ-TREE software with model prediction mode, which provided the best models WAG+F + R8 for lactate dehydrogenases and LG + F + R10 for the large subunit of the bidirectional hydrogenase.

Genomic features of *Cyanobium* sp. BSA11S that are pertinent to carbohydrate metabolism include glycolysis (ie, the Embden-Meyerhof-Parnas pathway) via (C) core modules, the complete (oxidative/nonoxidative) pentose phosphate pathway (PPP), glycogen storage/debranching, pyruvate oxidation, TCA cycle, and the Entner-Doudoroff pathway. Moreover, *Cyanobium* sp. BSA11S also harbors a lactate fermentation pathway with 2 different 2-hydroxyacid/D-lactate dehydrogenases (COG1052). These D-lactate dehydrogenases phylogenetically cluster with those of some other picocyanobacterial strains (ie, subcluster 5.2), and other anaerobes/facultative aerobes (ie, Planctomycetes, Verrucomicrobia, *Myxococcus*, *Vitosangium*, or *Archangium*, Fig. 3). In addition, *Cyanobium* sp. BSA11S encodes an additional A/V-type (archaeal/vacuolar-type) rotary ATP-ase, which can function as an ATP-driven proton (or Na^+^) pump to modulate cytoplasmic pH and ion homeostasis, or, under particular energetic conditions, operate in reverse to synthesize ATP from an existing ion gradient. The respective genes are located in close proximity to one of the D-lactate dehydrogenases (COG1052, here CDS3406/7), suggesting that they are part of the same regulon. Hydrogen maturation genes (namely *HypABCDEF*) and bidirectional hydrogenase genes (*HoxEFUYH*) are also encoded in the genome of *Cyanobium* sp. BSA11S, which suggests alternative ways of obtaining [H] and enabling NAD^+^ regeneration under oxygen-limited conditions. Our phylogenetic tree (Fig. 3) for the large subunit of the bidirectional hydrogenase (HoxH) placed subcluster 5.2 picocyanobacterial strains (alpha cyanobacteria) with other unicellular beta cyanobacteria, suggesting that hydrogenases in all unicellular cyanobacteria were likely inherited from a filamentous cyanobacterial ancestor. Additionally, the strain harbors the *acsF2-ho2-hemN2* gene cluster encoding proteins involved in pigment biosynthesis under oxygen-limited conditions.

### Transcriptomics under dark/light and anoxic/oxic conditions

To further explore the metabolic potential of *Cyanobium* sp. BSA11S for the various dark/anoxic pathways mentioned above, we compared gene expression profiles in picocyanobacteria under DA, DO, and LO conditions. Analysis of the transcriptomes showed that light strongly controlled gene expression. Indeed, PERMANOVA revealed that 72% of the variance was explained by light, and only 3% by oxygen. In the PCA ordination space, the LO data formed a clearly separated cluster with respect to the DO and the DA treatments, which grouped together both at the raw-read similarity levels and with regard to the CDS expression (Fig. S3).

**Figure 4:**
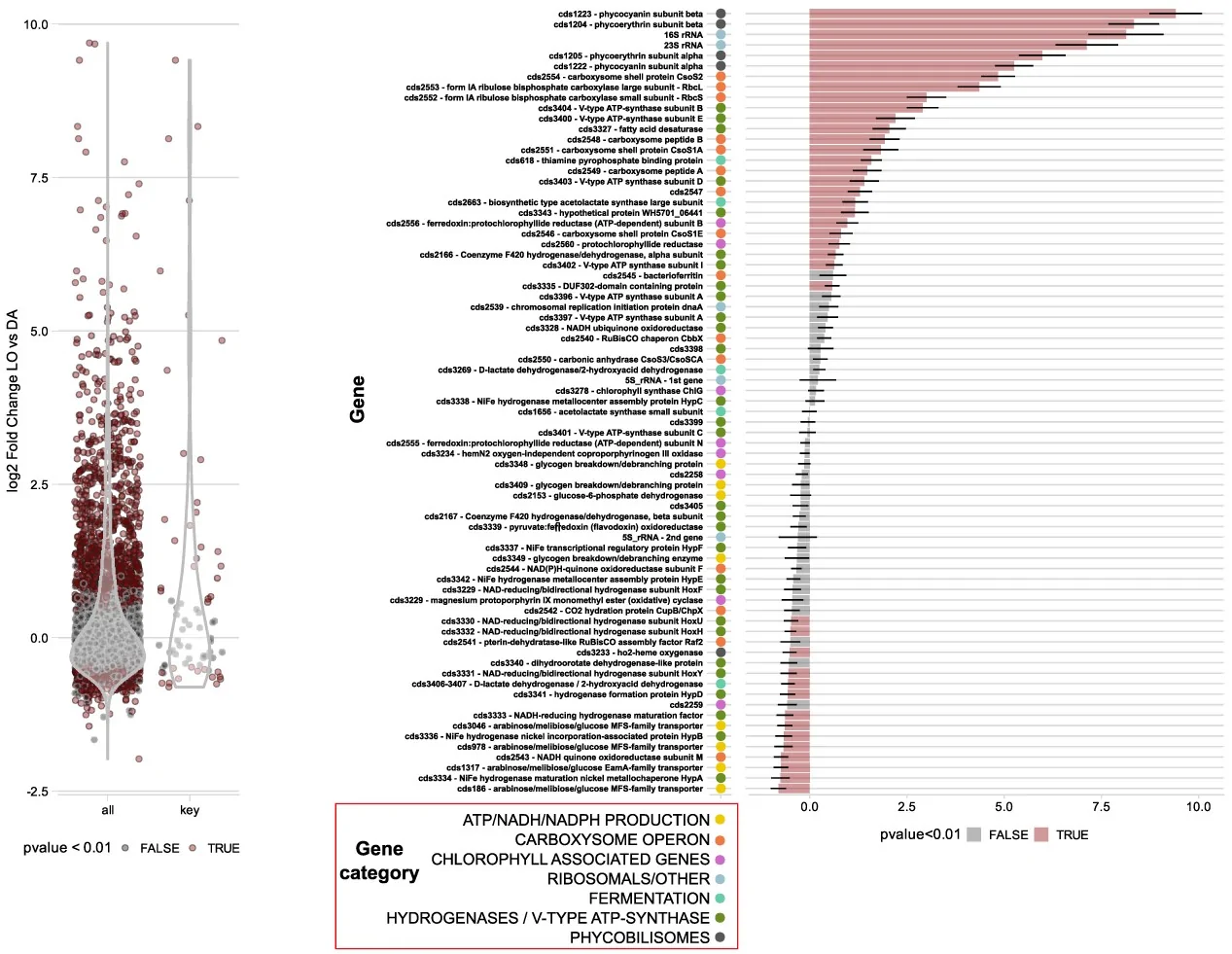
Up- and down-regulation of BSA11S genes (log2Fold change up to 10) under light-oxic (LO) vs dark-anoxic (DA) conditions. The red colour of dots and bars indicates significant (true) change as log2-fold change and a *P*-value <0.01. Grey dots and bars indicate non-significant changes. Left: Fold change of all analysed genes (all) and a subset of key genes (inside a red box) under the following categories: ATP/NADH/NADPH production, carboxysome operon, chlorophyll associated genes, ribosomals/other, fermentation, hydrogenases/V-type ATP-synthases and phycobilisomes. Right: List of key genes relatively overexpressed in the LO treatment (positive values), and genes that are relatively overexpressed under DA conditions (negative values). Different genes are circle-colour coded according to different metabolic pathways or processes.

Given that the transcriptomic analyses revealed relatively similar gene expression profiles for DA and DO conditions (Fig. S3), especially for some key genes which we compared below (Fig. 4, Fig. S4, and Fig. S5), we focused on the “most extreme/different” comparison between LO and DA (Supplementary Dataset 2). Comparing LO and DA treatments, we observed in LO treatments a higher expression (log2-fold change up to 10) of genes involved in photosynthesis (ie, phycoerythrin and phycocyanin alpha/beta subunits, CDS1204–1205–1222-1223) and the synthesis of carboxysome components like carbonic anhydrase and cellular RuBisCO form IA (CDS2546–2554, see Fig. 4 and Supplementary Dataset 2), which we used as a positive control to distinguish treatments. Likewise, genes involved in glycolysis (Embden-Meyerhof-Parnas pathway) and in pyruvate oxidation (CDS1550–1642-1850) exhibited higher gene expression levels in LO compared to DA conditions, whereas genes involved in the Entner-Doudoroff pathways did not show any significant increase in gene expression under LO compared to DA conditions. (Supplementary Dataset 2).

In fact, while there was a general trend of downregulation of 1458 genes under DA compared to LO conditions (with log2-fold changes >0.1), 1594 genes were moderately upregulated (Fig. 4, Fig. S4, supplementary text, and Supplementary Dataset 2), with log2-fold changes between −0.1 and −2 under LO compared to DA conditions (Fig. S4 and Supplementary Dataset 2). Notably, this upregulation was observed for arabinose/melibiose/glucose MFS family of transporters (CDS186, CDS482, CDS978, CDS1317, CDS3046), the glycogen breakdown/debranching enzymes (CDS3409, CDS3348, CDS3349), and the glucose-6-phosphate dehydrogenase (CDS2153), involved in the first step of the PPP pathway, and with that, associated with ATP, NADH, and NADPH production. Genes involved in fermentation, such as D-lactate dehydrogenase (CDS3407–8), and both hydrogenase maturation (*hyp* genes) and bidirectional hydrogenases (*hox* genes) (Fig. 4 and Supplementary dataset 2), were also moderately upregulated under DA compared to LO conditions, with log2-fold rates changing between −0.1 and −0.55. Notably, the vast majority of the above-mentioned key genes were moderately overexpressed in DA vs DO, which is the reason why we focused all comparisons on DA vs LO (Fig. S5).

While there was no significant difference in the expression of some chlorophyll *a–*associated genes (ie, *chlG* or light/dark-dependent NADPH:protochlorophyllide oxidoreductase - LPOR/DPOR) under LO versus DA conditions, we observed a log2-fold increase between 0.1 and 0.5 in DA compared to LO conditions for other LPOR-like short-chain dehydrogenase (SDR) variants (CDS2258–2259), as well as for *acsF2, ho2, hemN2* genes (CDS3229, 3233, and 3234, respectively). These genes are involved in chlorophyll, phycocyanobilin, and tetrapyrrole (heme) biosynthesis, respectively, also under oxygen-limited conditions (Fig. 4 and Supplementary Data set 2).

## Discussion

**Figure 5:**
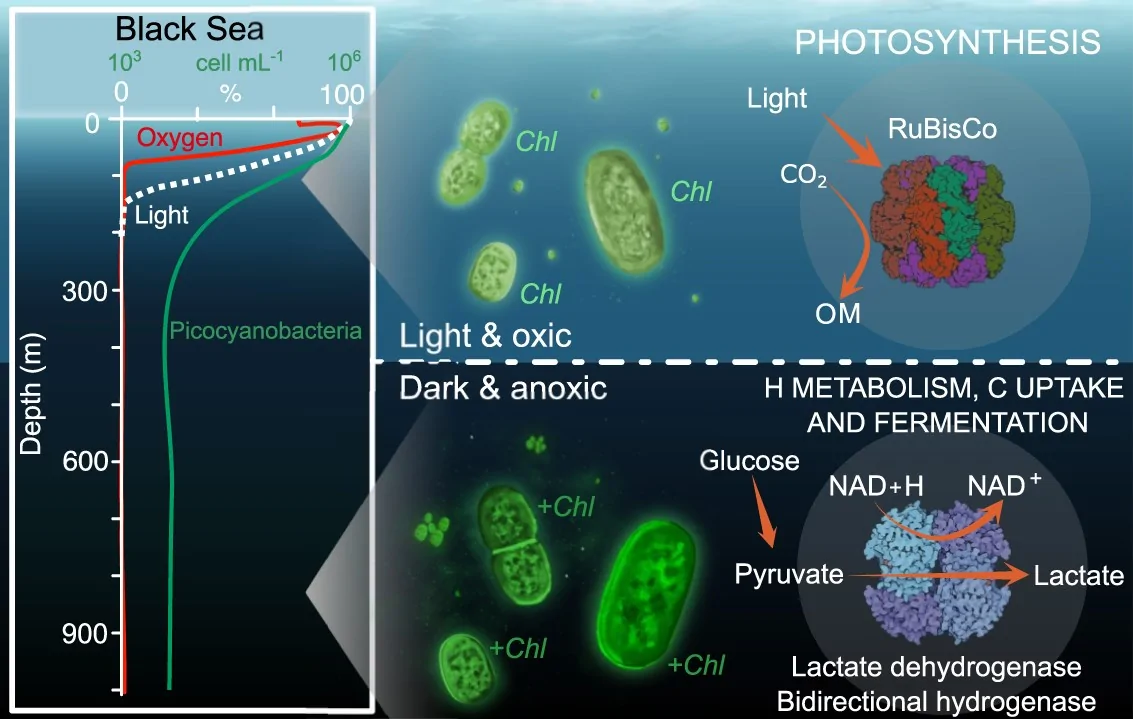
Photoautotrophic (photosynthesis; upper panel) and heterotrophic (hydrogen metabolism, fermentation; lower panel) life modes in the picocyanobacterium *Cyanobium* sp. BSA11S from the Black Sea (picocyanobacterial abundance, oxygen and light profiles on the left panel). This schematic illustrates adaptation to transient conditions between oxic and anoxic habitats, under both light and dark conditions. In the presence of light, picocyanobacteria use inorganic carbon as a substrate for organic matter production; in the absence of light, they exploit internal or extracellular organic carbon (ie, glucose, glycogen) to generate ATP and NADH, while pyruvate-to-lactate conversion and hydrogen metabolism are used to regenerate NAD^+^.

Our experiments show that *Cyanobium* sp. BSA11S can survive under dark anoxic conditions, exploiting simple organic substrates and using the acquired energy to produce pigments (see overview in Fig. 5, graphical abstract). Being pigmented does not provide an obvious advantage in the absence of light, but it may be beneficial under variable conditions in highly dynamic habitats. Indeed, combining laboratory-based experiments, single-cell isotope probing, and transcriptomics, we showed that picocyanobacteria from the BS accumulate chlorophyll and have the ability to adapt to various C sources in anoxic/euxinic waters, where diverse pathways for exploiting organic carbon as an energy source have been identified in oceanic taxa. This finding is evidenced by the metagenome assembled genomes (MAGs) obtained from depths of 150 and 750 m [44]. Moderate gene expression from our transcriptomics experiments revealed that the adaptation to dark anoxic conditions likely involves several steps: (i) uptake/transport of C sources and utilization of stored C (glycogen degradation), (ii) ATP, NADH, and NADPH production through the pentose phosphate pathway (via glycerol-6-phosphate dehydrogenases), and (iii) NAD^+^ regeneration through hydrogen metabolism (via bidirectional hydrogenases) coupled with the reduction of pyruvic acid (ie, glucose fermentation to lactate; lactic acid fermentation).

Cyanobacterial hydrogenases have been studied for decades as alternative proton uptake mechanisms coupled to N_2_ fixation [15]. In addition, there has been a growing interest in characterizing the potential of hydrogen production by different species, including *Synechocystis* [44, 45]. Adaptation to perform fermentation is widespread among filamentous cyanobacteria from low-oxygen environments, such as microbial mats [9]. However, the presence of lactate dehydrogenases and the potential involvement of hydrogenases as energy valves in picocyanobacteria from the cluster 5 (sub-cluster 5.2) point towards a novel evolutionary trait in the globally most abundant phototrophs. Indeed, certain representatives of this cluster seem to retain specific ancestral characteristics, as evidenced by the close phylogenetic relationship of their bidirectional hydrogenases and lactate dehydrogenases (COG1052) to those in anaerobes or facultative aerobes (Fig. 3), such as members of the PVC superphylum and Myxobacteria [46]. In the case of hydrogenases, phylogenetics suggests that they were inherited by all unicellular cyanobacteria from a filamentous cyanobacterial ancestor (Fig. 3). Notably, these genes are completely absent in all marine Ca. Marinosynechocccus-SC5.1 and *Prochlorococcus* isolates but are present in various *Cyanobium*-SC5.2 from freshwater and brackish sources. This suggests that several important ancestral anaerobic pathways have been maintained in freshwater systems—possibly inherited from filamentous and other cyanobacterial lineages—but lost in well-oxygenated marine waters [3]. On the other hand, other members within the cluster 5 of picocyanobacteria retain genes enabling fermentative pathways as well as genes coding for other oxygen-sensitive enzymes. As for the latter, we emphasize here the significance of these enzymes in the biosynthesis of pigments such as chlorophyll, heme, and phycobilins (acsF2, ho2, and hemN2, respectively), which were recently identified in *Prochlorococcus* inhabiting oceanic oxygen minimum zones (OMZs), and in various *Synechococcus* strains [6]. These genes were previously examined in cyanobacterial model organisms, including the freshwater *Synechocystis* PCC6803 and the euryhaline/coastal/marine *Synechococcus* PCC7002, and exhibited high expression levels exclusively under oxygen-limited conditions [47, 48].

A noteworthy recent discovery regarding the adaptive abilities of picocyanobacteria is their capacity for living photoautotrophically, as well as photoheterotrophically [12]. This nutritional mode has been termed “mixotrophy” [12]. In the absence of light, picocyanobacteria rely solely on glucose, fructose, or sucrose as source of energy [9, 12, 13]. This behavior has been documented primarily in marine picocyanobacteria (SC5.1 and *Prochlorococcus*) [12, 13]. Our results provide additional insight into these capabilities in *Cyanobium*-SC5.2*,* which incorporates and uses organic carbon to support heterotrophic growth through glucose-6-phosphate dehydrogenase (catalyzing the first step of the PPP pathway), glycogen-debranching enzymes, or the fermentative transformation of glucose to lactate, albeit the culture was not fully axenic, the picocyanobacterium represented ca. 95% of the cells, minimizing the noise of transcriptomics and NanoSIMS. Our data support the potential for mixotrophy previously detected in marine picocyanobacteria and extend this capability to freshwater and brackish representatives. This finding adds to the broad spectrum of metabolic plasticity observed within this group of small photosynthetic microorganisms [3, 49].

Our findings, albeit limited to a single picocyanobacterium strain that accumulates chlorophyll in the dark and generates energy via fermentation, nevertheless enable further clarification of cyanobacterial evolutionary pathways during a period in Earth’s history when the Ocean was largely suboxic or anoxic [50]. In this context, dark incubations of *Cyanobium* sp. BSA11S revealed a significant release of carbohydrate-like exudates (see Table S3). These compounds have been proposed to fulfil important supplementary functions in photoautotrophs, including, for example, the removal of toxic metabolites, acting as signalling molecules [51]; improved acquisition of trace elements, as well as osmoprotection; and additional defence against heterotrophs [52]. We speculate that in the dark anoxic waters of the BS (and possibly in other similar habitats, both contemporary and ancient), the primary advantage of releasing carbohydrate-like exudates was associated with enhanced sorption and bioaccumulation of trace metals.

The metabolic versatility observed in picocyanobacteria from the BS provides insights into the evolution and transitional nature of euryhaline/brackish cyanobacteria [3]. This is important, given that the most abundant phototrophs on Earth, ie, marine *Prochlorococcus* and *Marinosynechococcus*-SC5.1, evolved from a freshwater/brackish ancestor belonging to SC5.2 (*Cyanobium*) [3, 53–55]. In turn, many members of the SC5.2 (including BS isolate) retain some of the ancestral anaerobic pathways [3] that were donated from filamentous and/or other ancient (unicellular) cyanobacteria (Fig. 3). Since halotolerance is one of the common features in picocyanobacteria from SC5.2, it was likely developed in BS picocyanobacterial populations [3], when salinity changed dramatically between ~128′000 and 65′000 years ago [56]. Throughout this period, endemic picocyanobacteria likely experienced regime shifts and niche differentiation across diverse habitats (ie, marine, brackish, and freshwater). Our findings thus imply that metabolically versatile and adaptable cyanobacteria, already present in the ancient oceans and other aquatic systems, were likely well equipped to withstand some of the most challenging periods in their evolutionary history.

## Supplementary Material

Web_Material_ycag159

## Data Availability

All data derived from this work is publicly available in the NCBI-Genbank databases.
