# Supplementary material for: Black Sea picocyanobacteria exploit ancestral traits for energy and chlorophyll production in dark anoxia
Source: ISME Commun. 2026 Jul 17;6(1):ycag159. doi: 10.1093/ismeco/ycag159 (PMC13588713; doi:10.1093/ismeco/ycag159)
Supplement: Supplementary file 1 [file web_material_ycag159.zip › Supplementary NanoSIMS Images.pdf]

Control C1

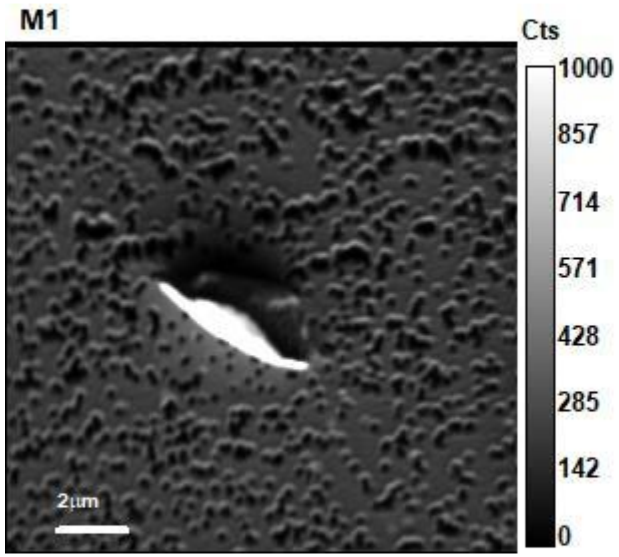

Im-1, D1-4,15x15, 4 layers.

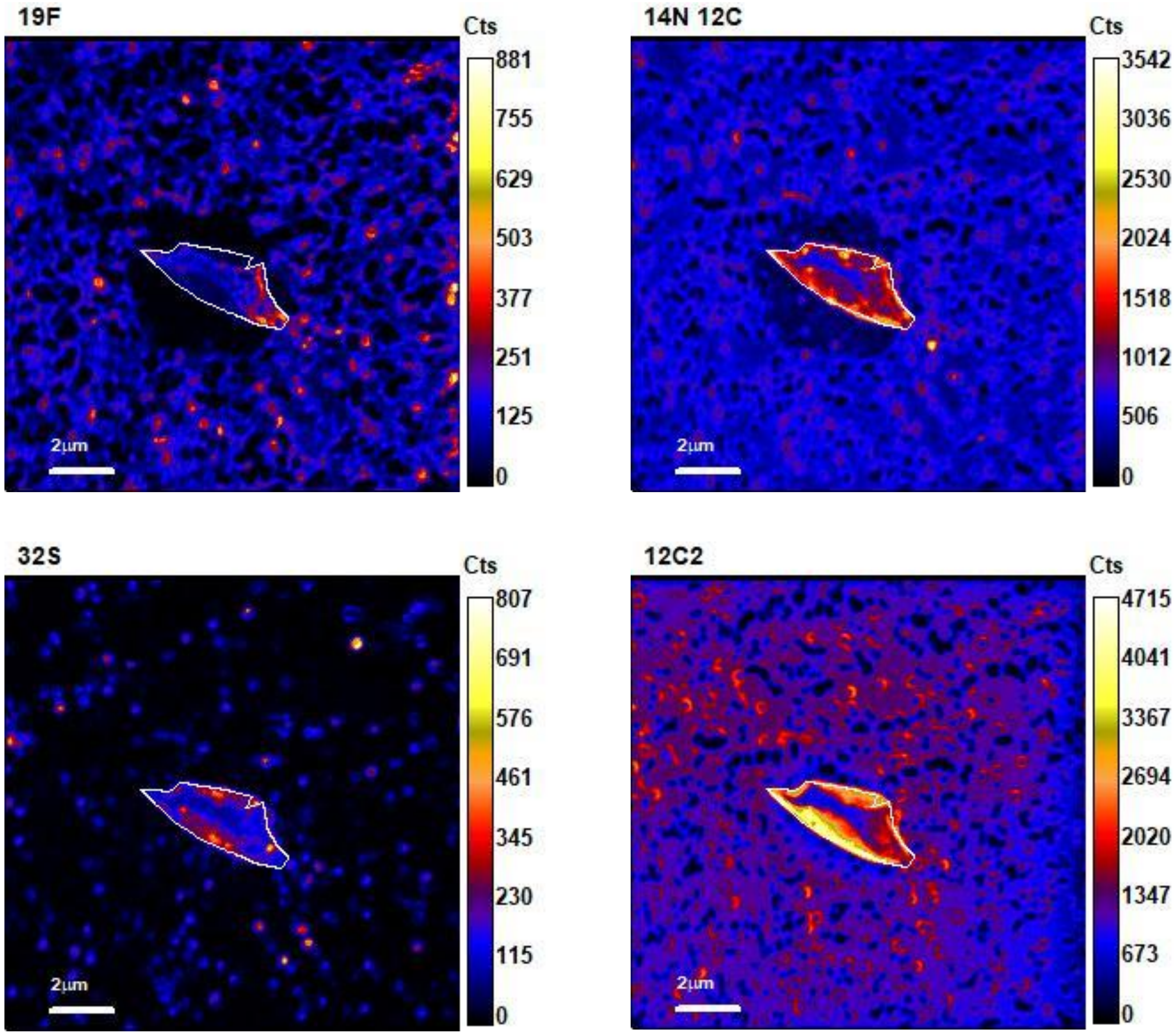

Control C1

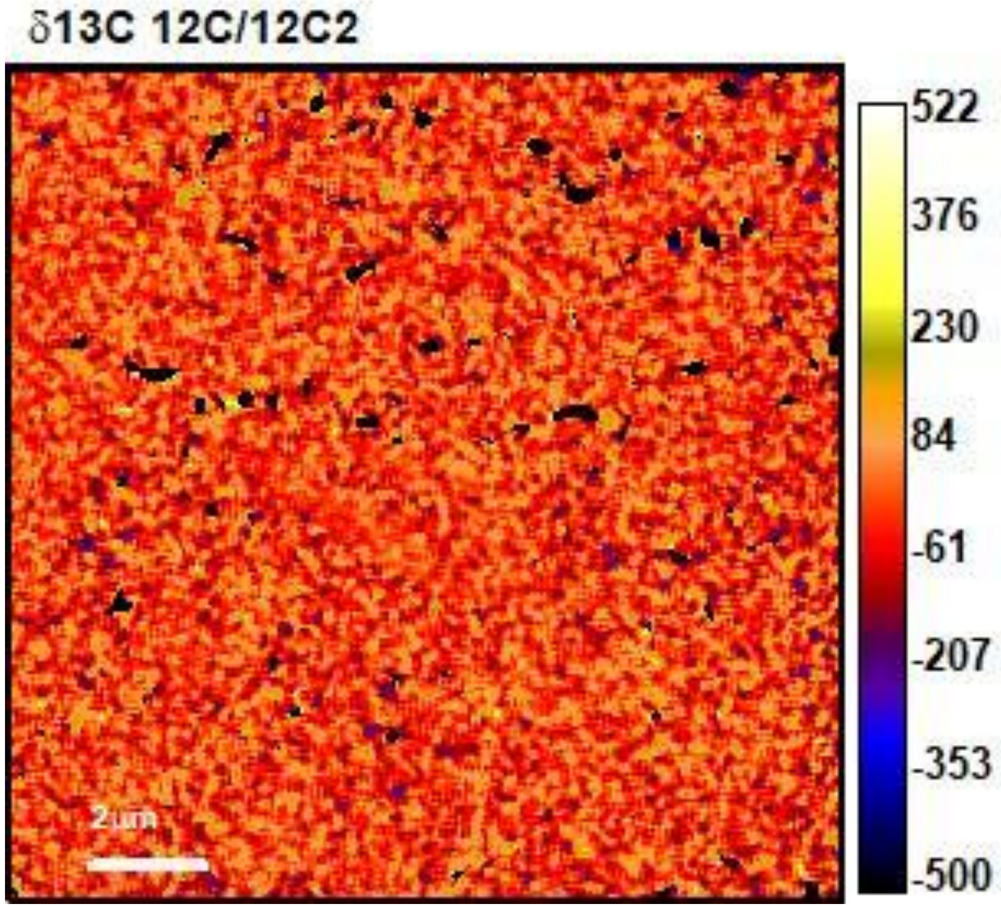

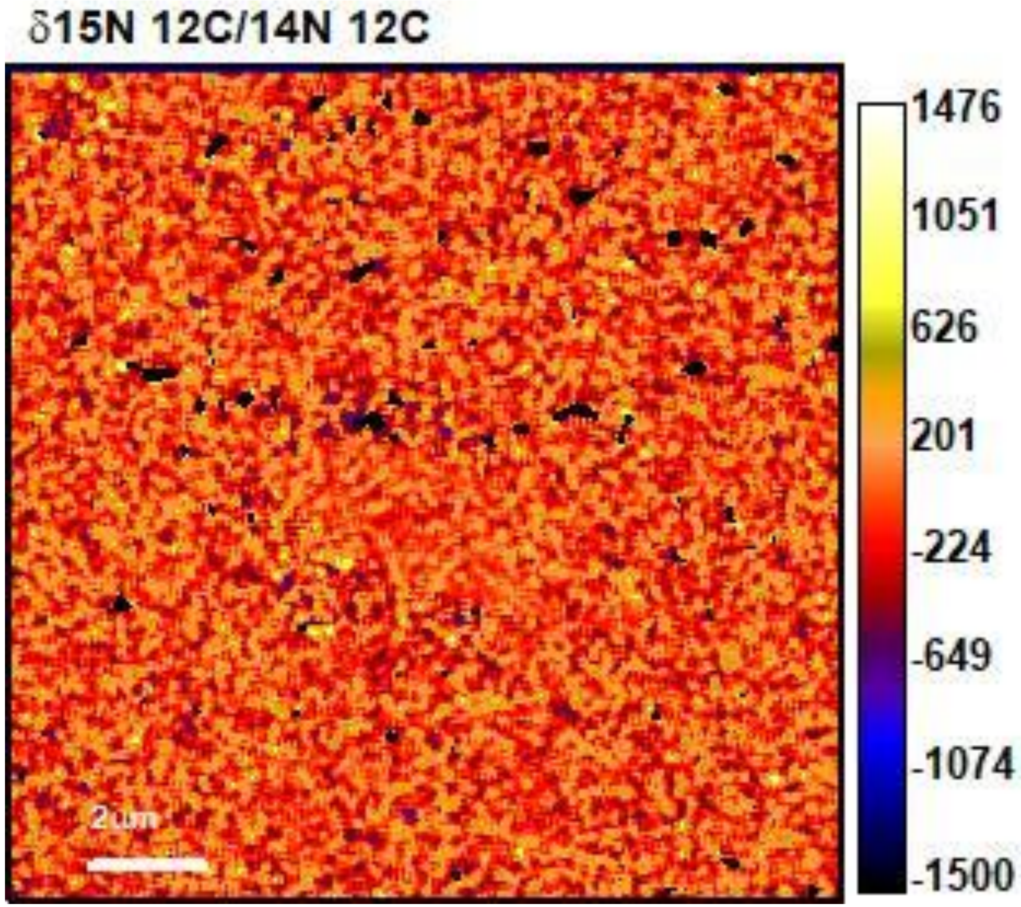

No enrichment

13C12C/12C2~0.02143±7

15N12C/14N12C~0.00374±4

Sample DA1

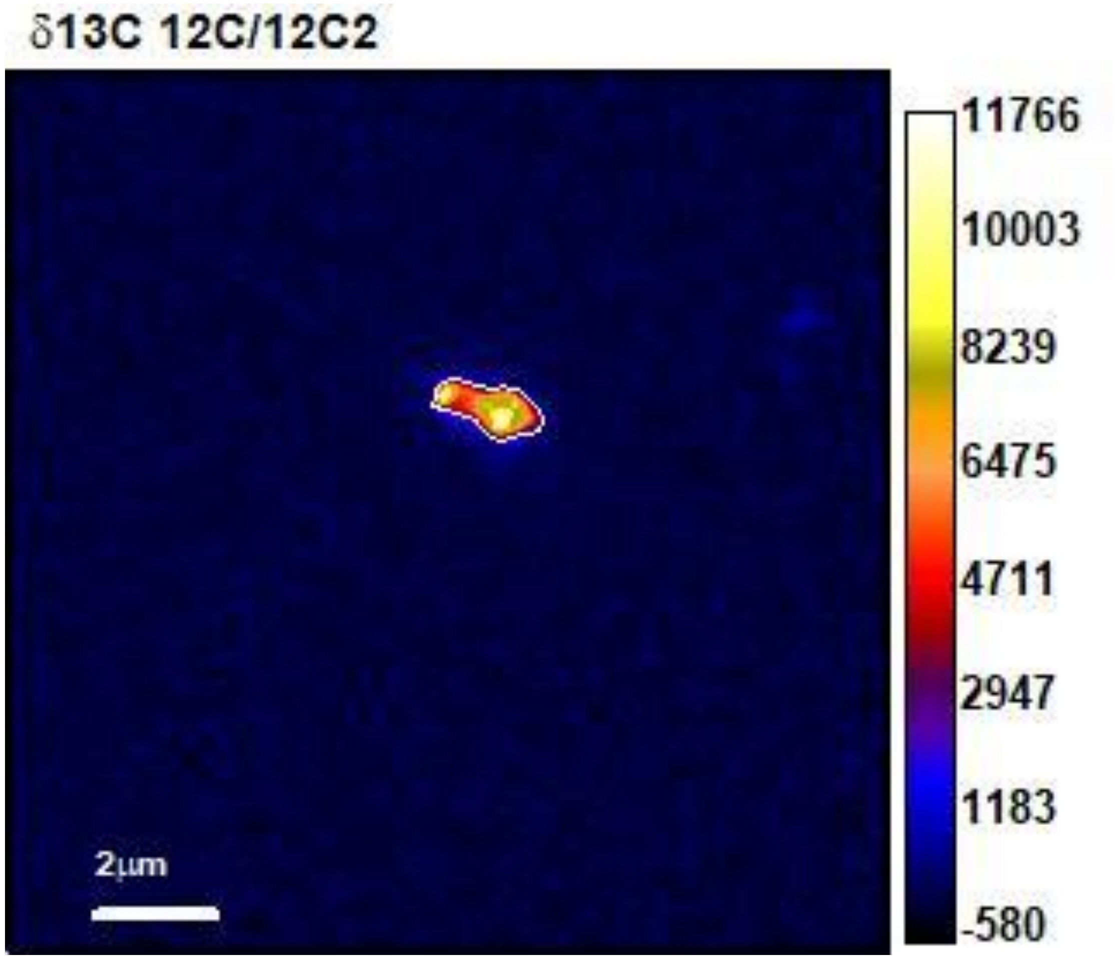

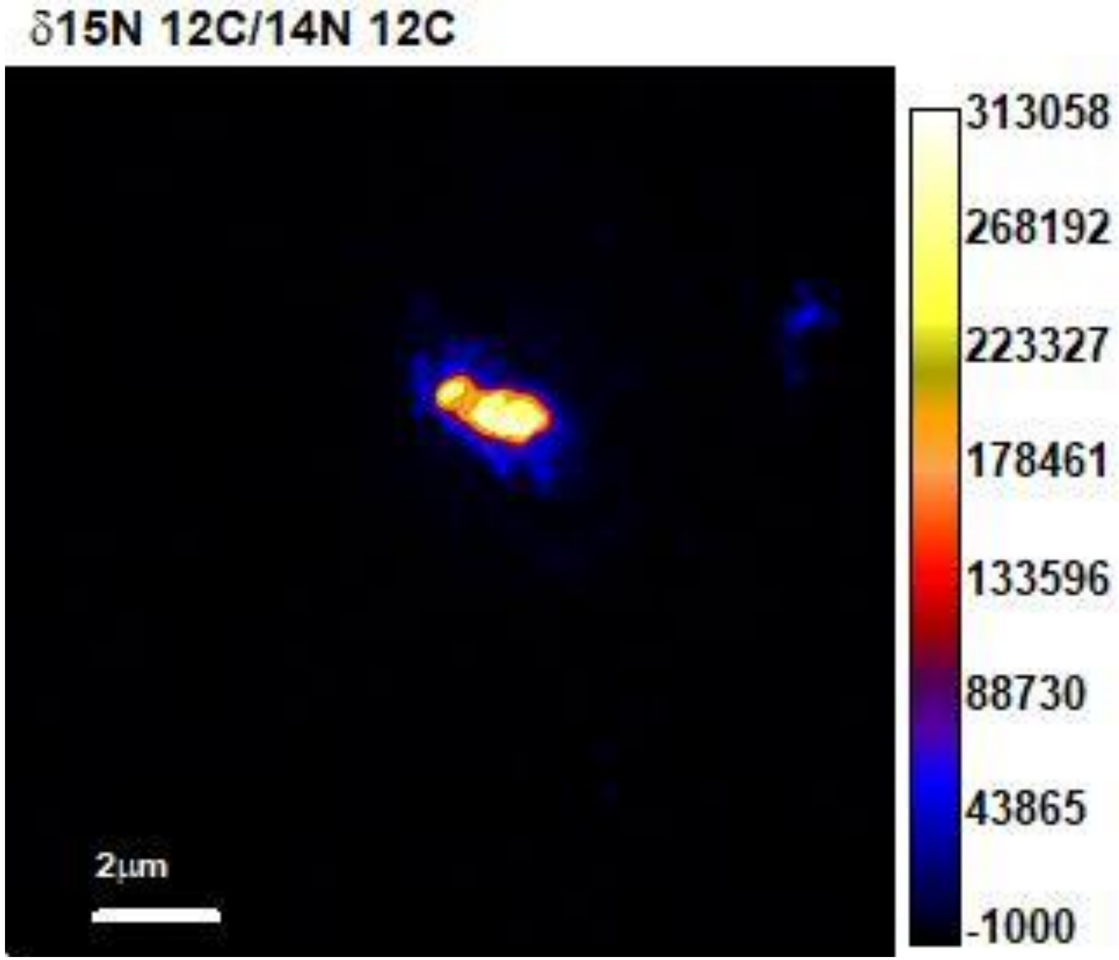

Im-2, D1-4,15x15, 4 layers.

δ-13C12C/12C2~6015±48

δ-15N12C/14N12C~245492±1180

Sample DA1

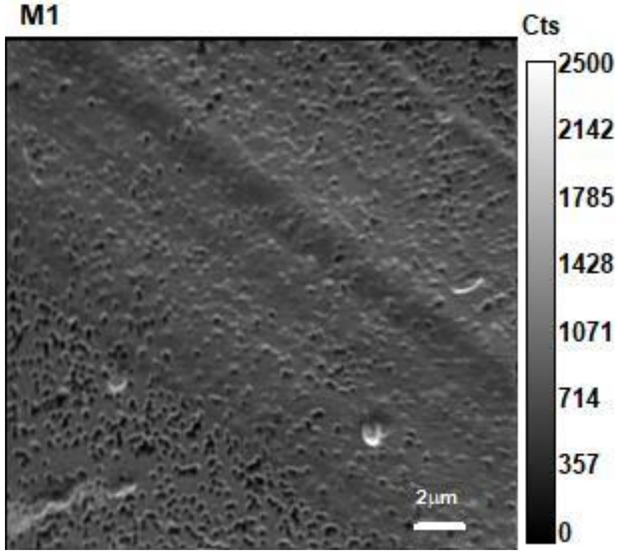

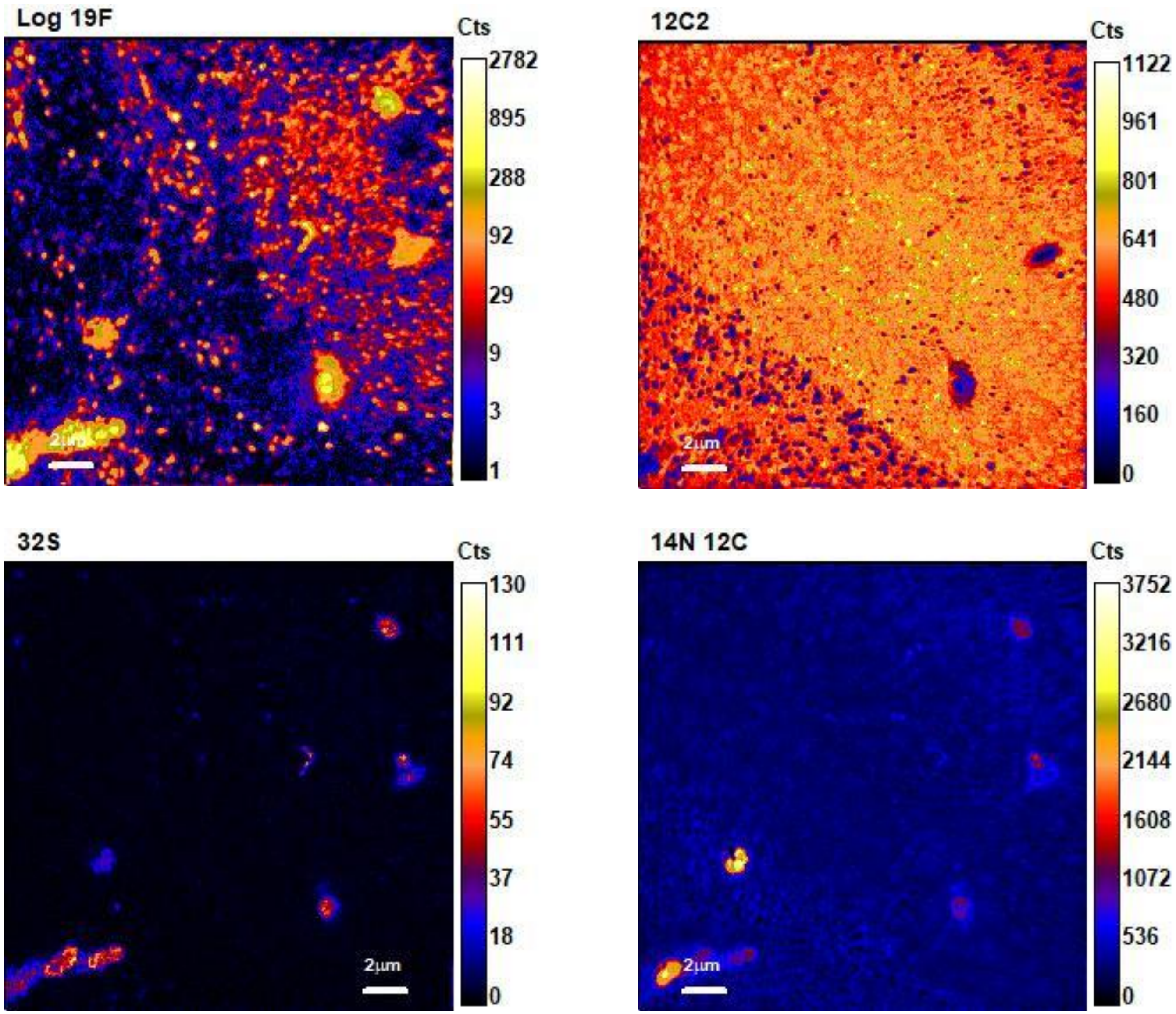

Im-3, D1-4,22x22, 10 layers.

Sample DA1

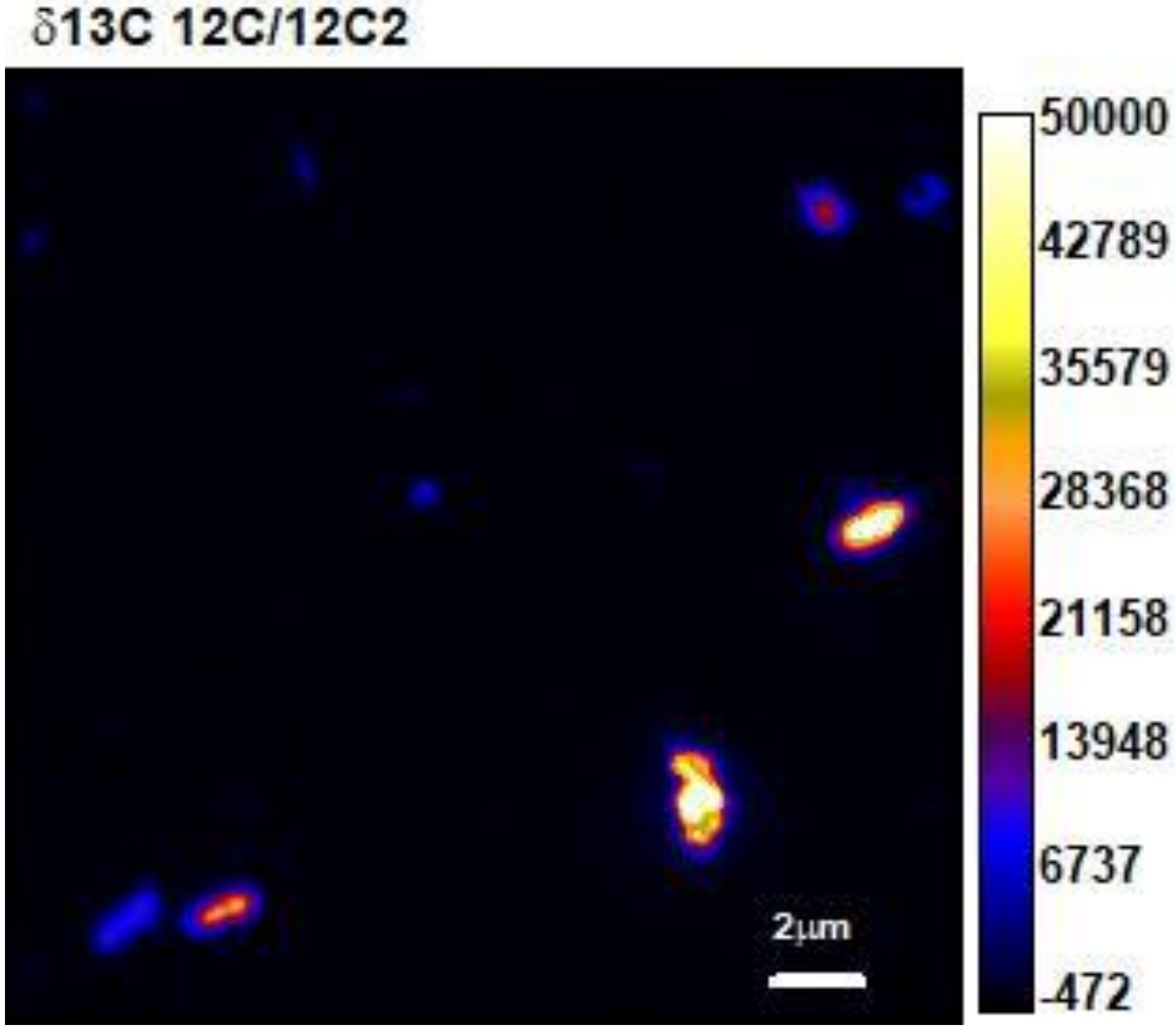

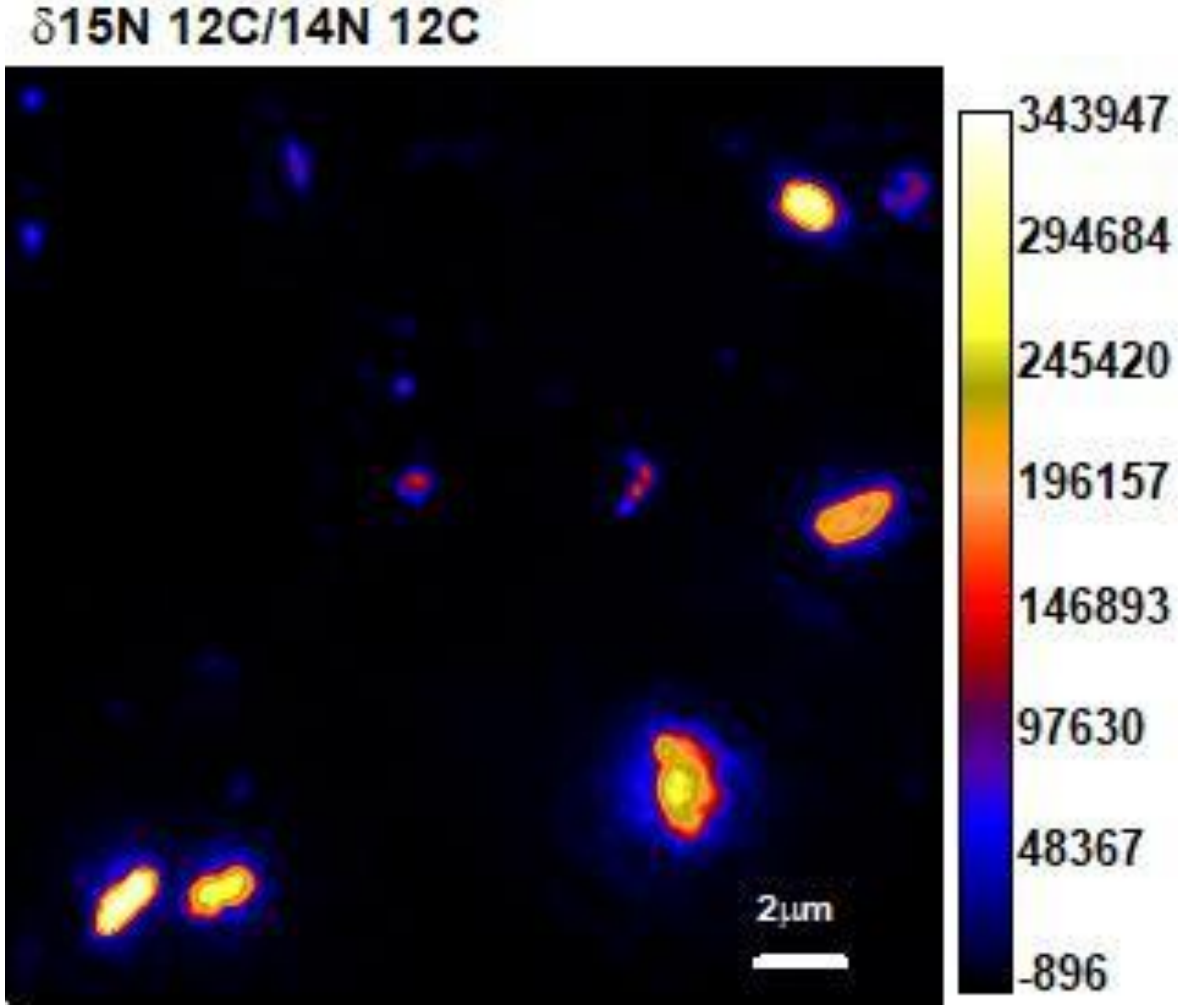

Im-3, D1-4,22x22, 10 layers.

Several ROIS to be defined

Control C1

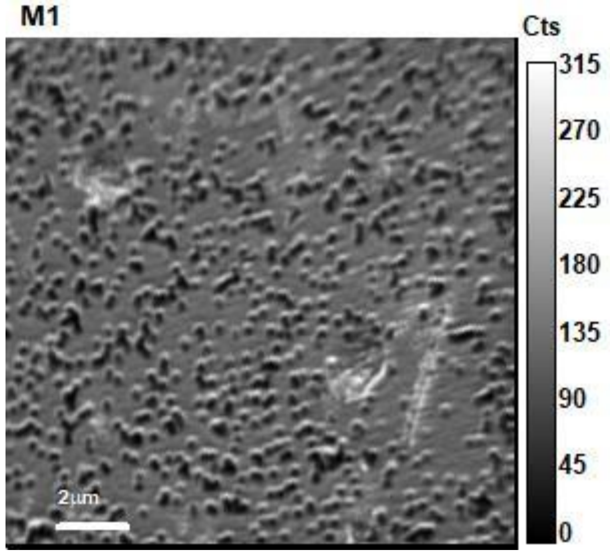

Im-4, D1-4,15x15, 4 layers.

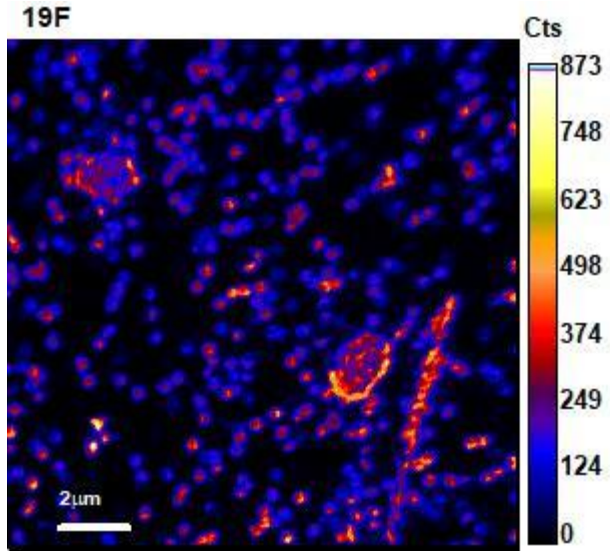

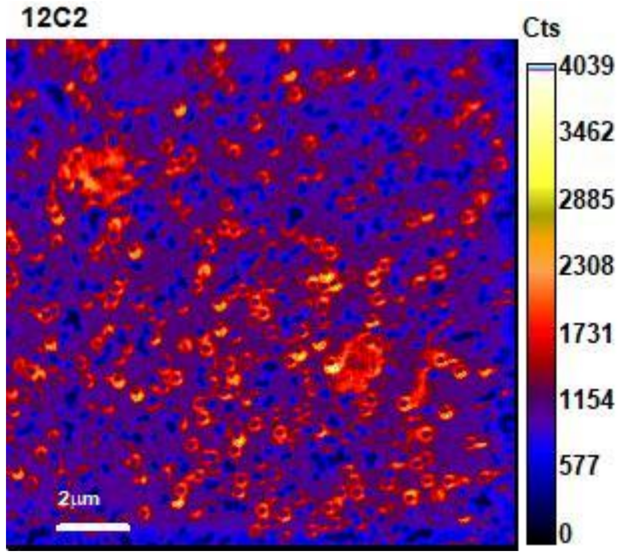

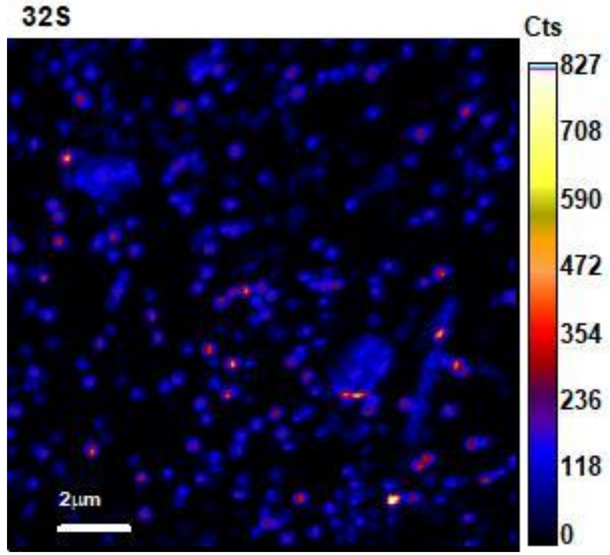

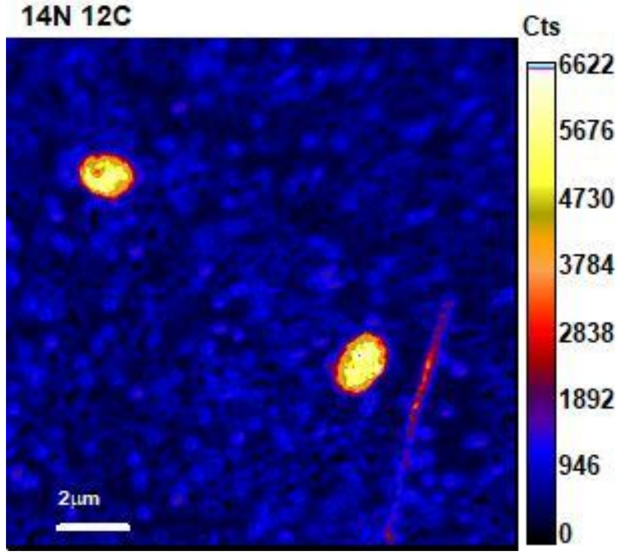

Control C1

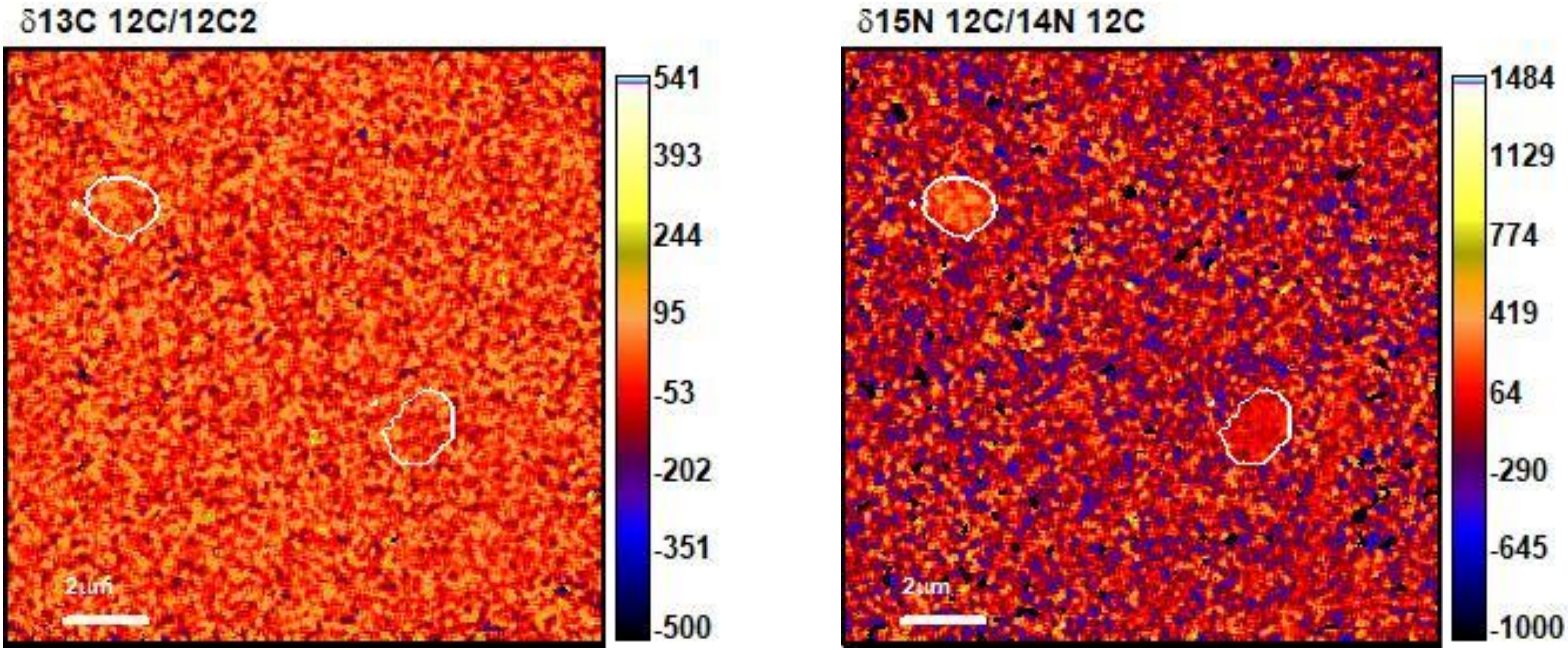

No enrichment

δ-13C12C/12C2~14 and 22
δ-15N12C/14N12C~65 and 232

Sample LO1

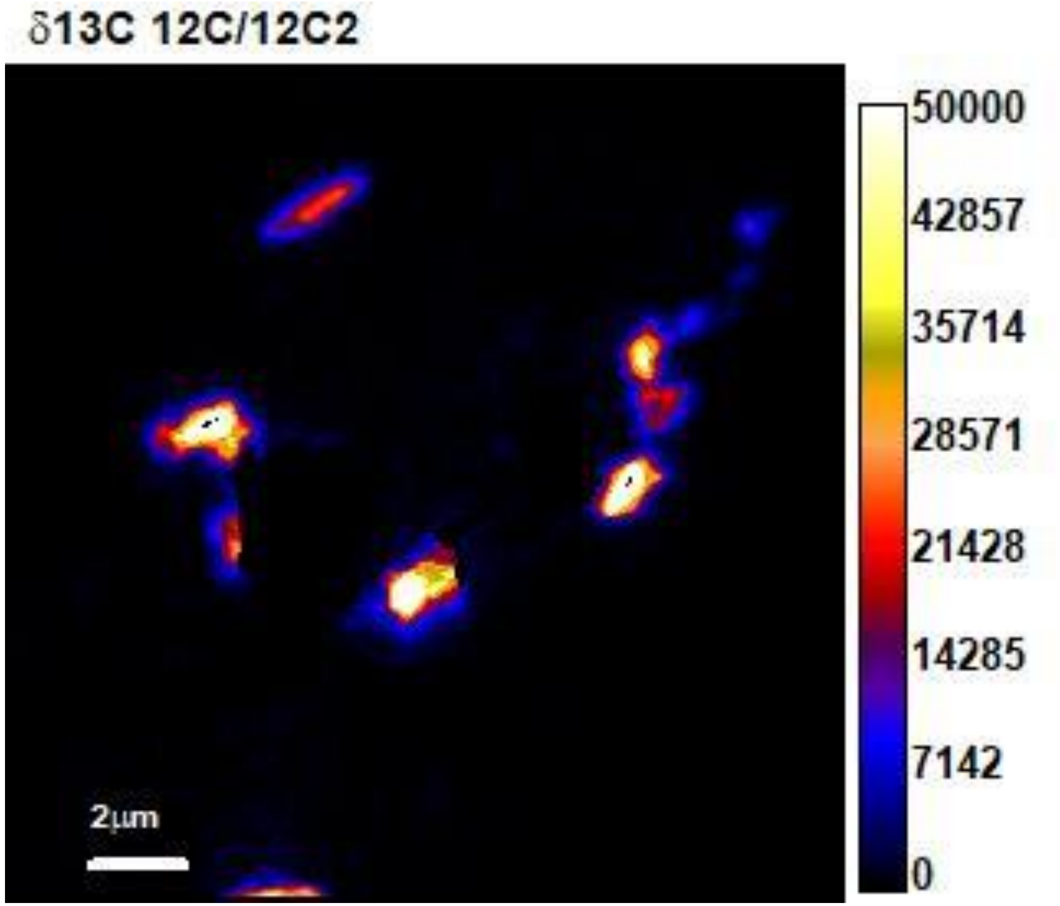

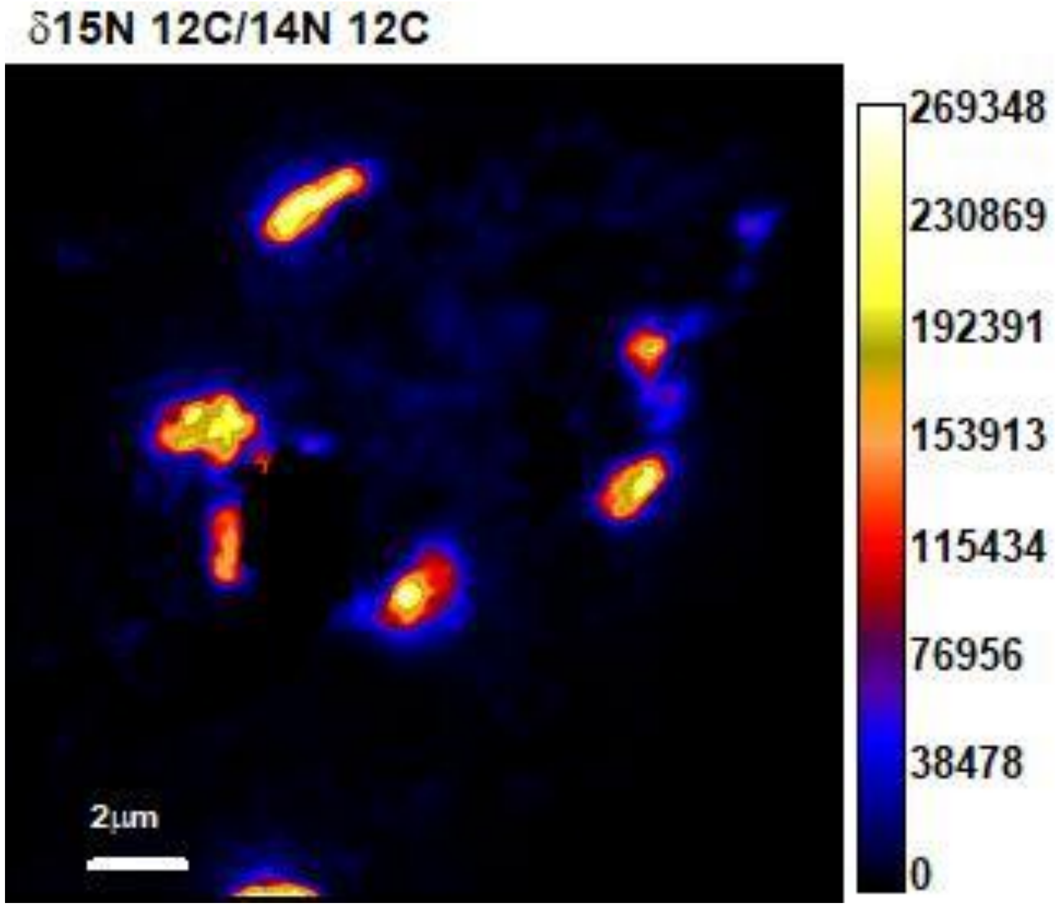

Im-6, D1-4,20x20, 4 layers.

Several ROIS to be defined

Sample DA2

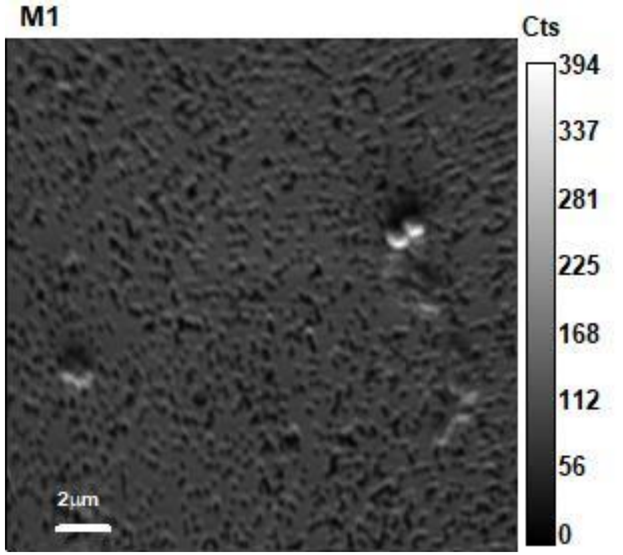

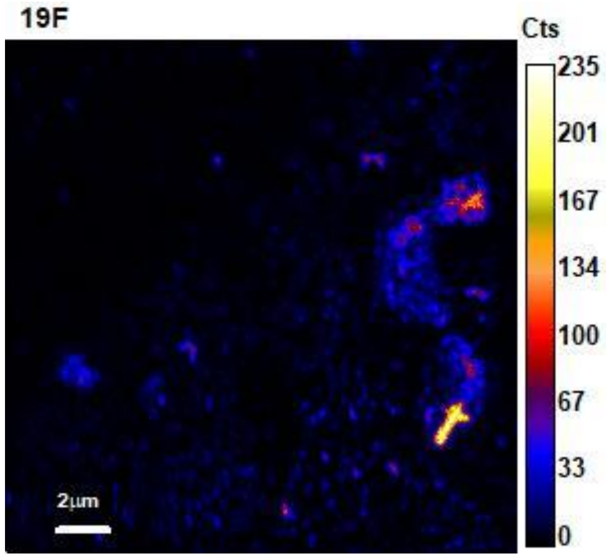

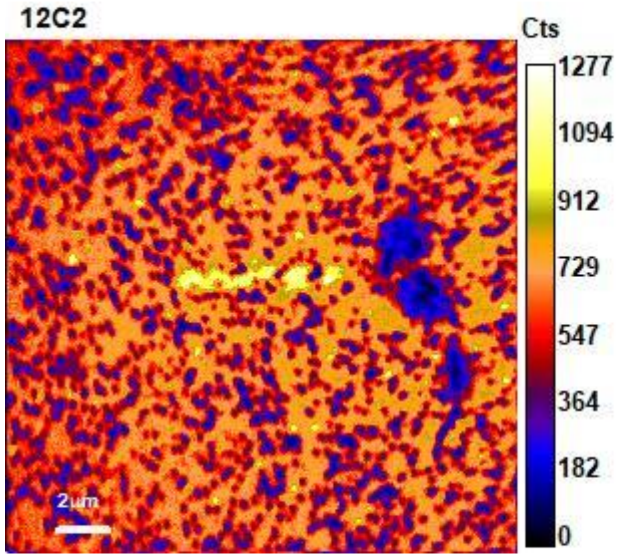

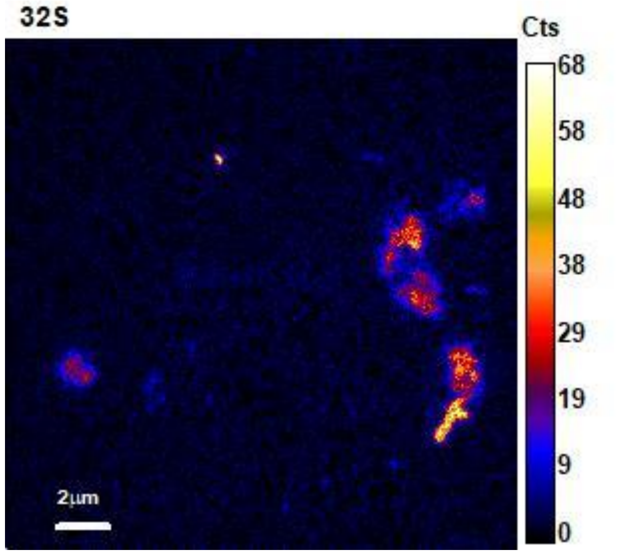

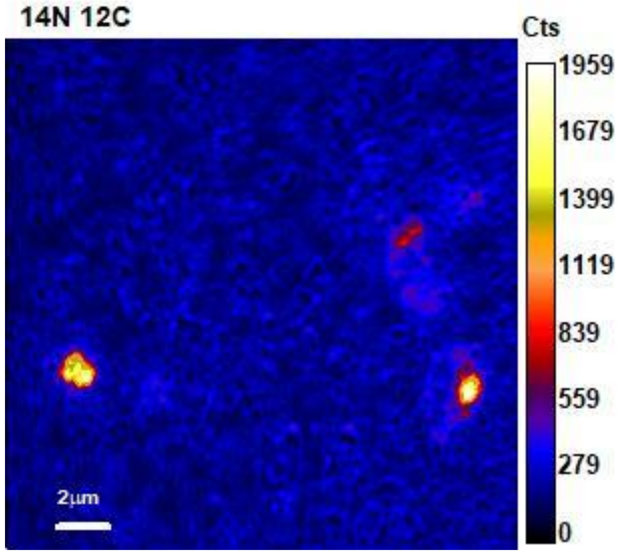

Im-7, D1-4,20x20, 4 layers.

Sample DA2

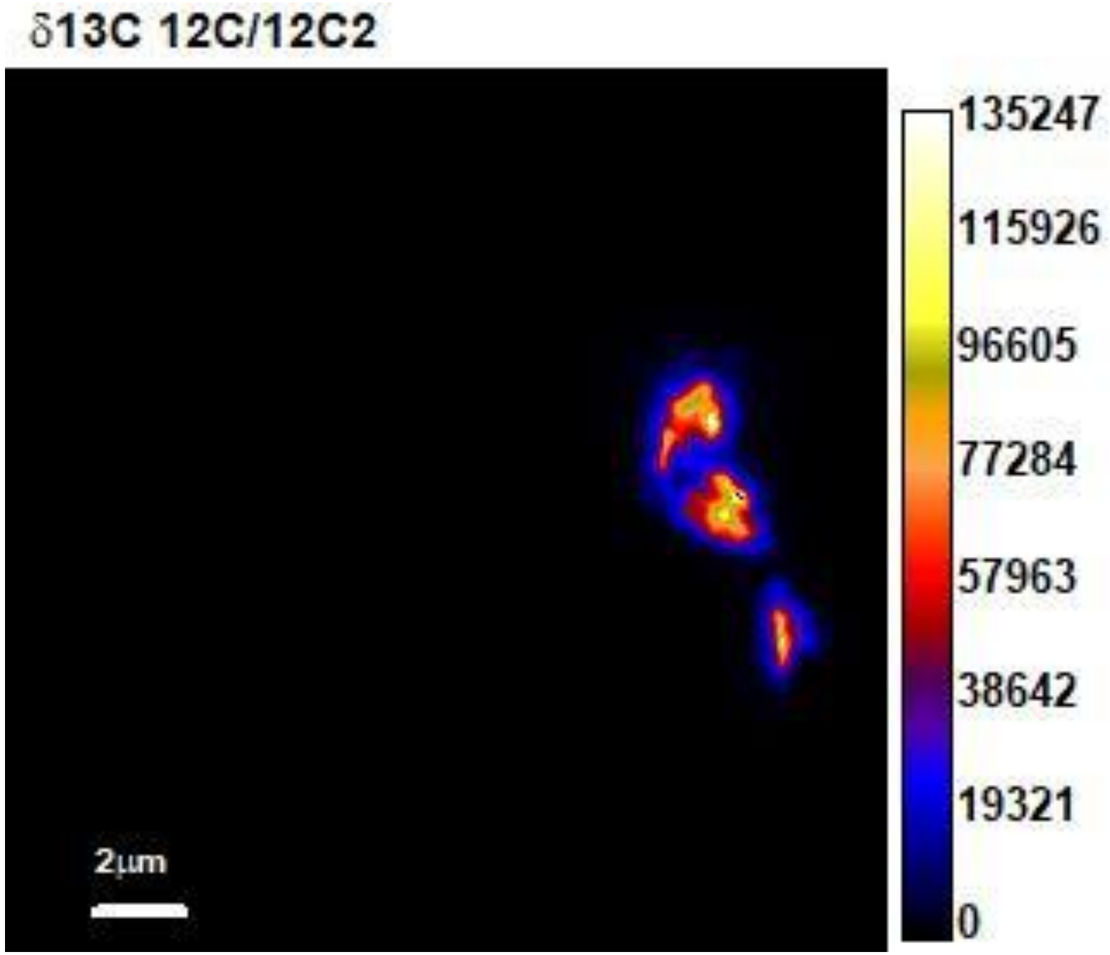

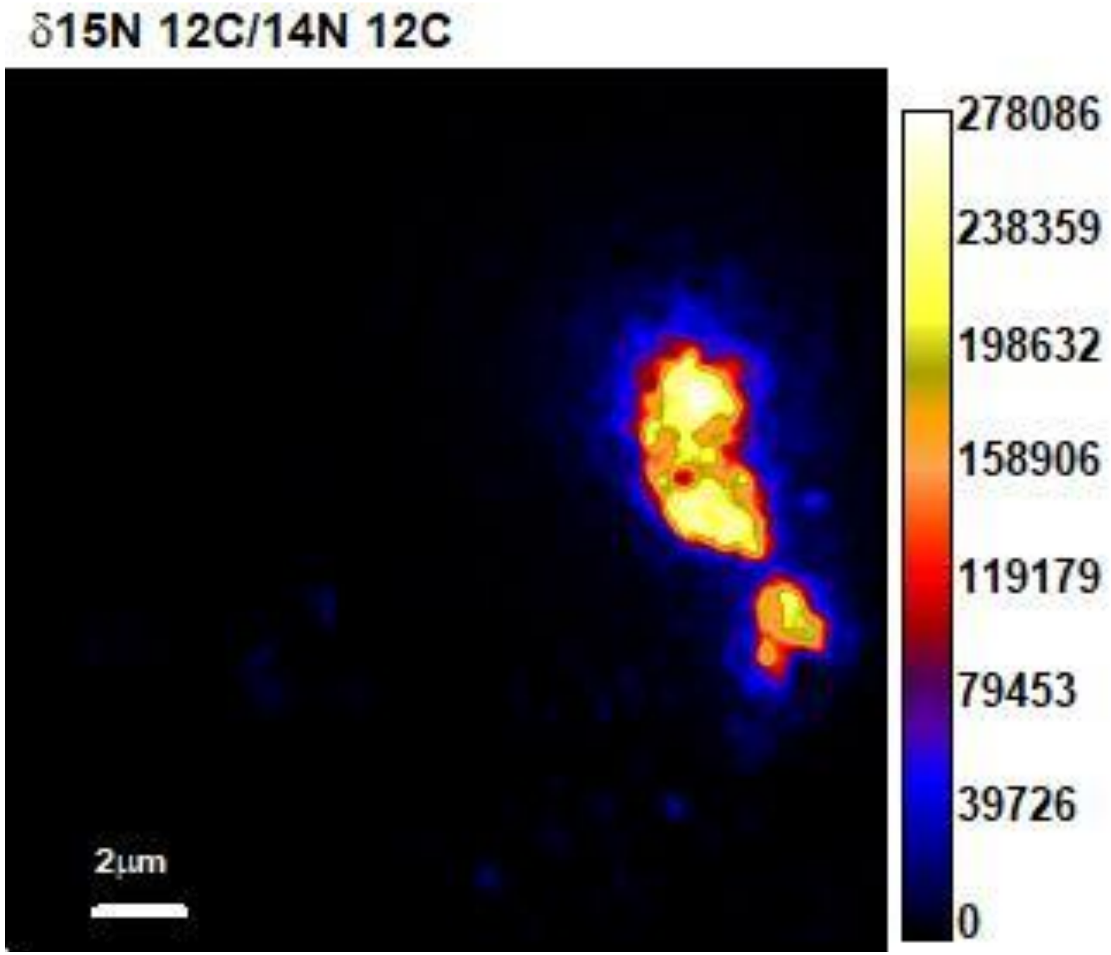

Im-7, D1-4,20x20, 4 layers.

Several ROIS to be defined

Sample LO4

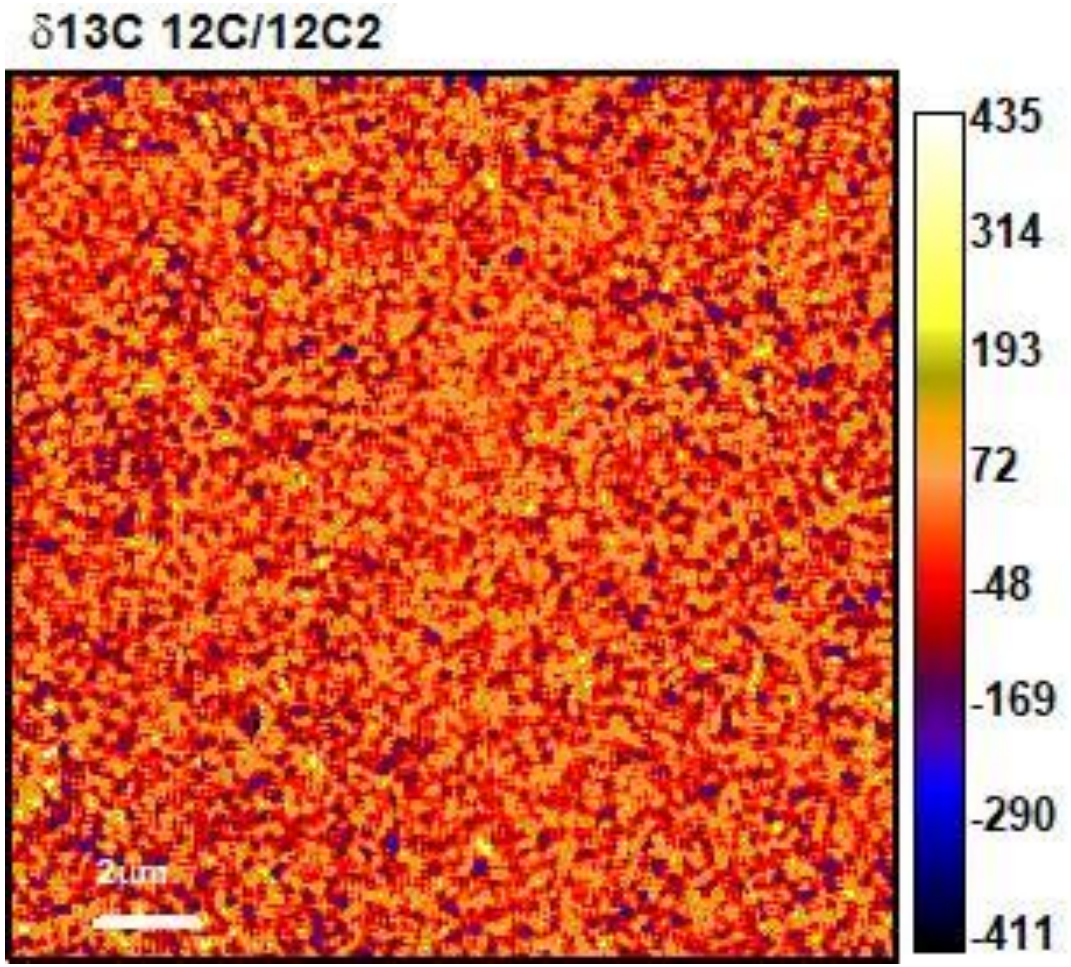

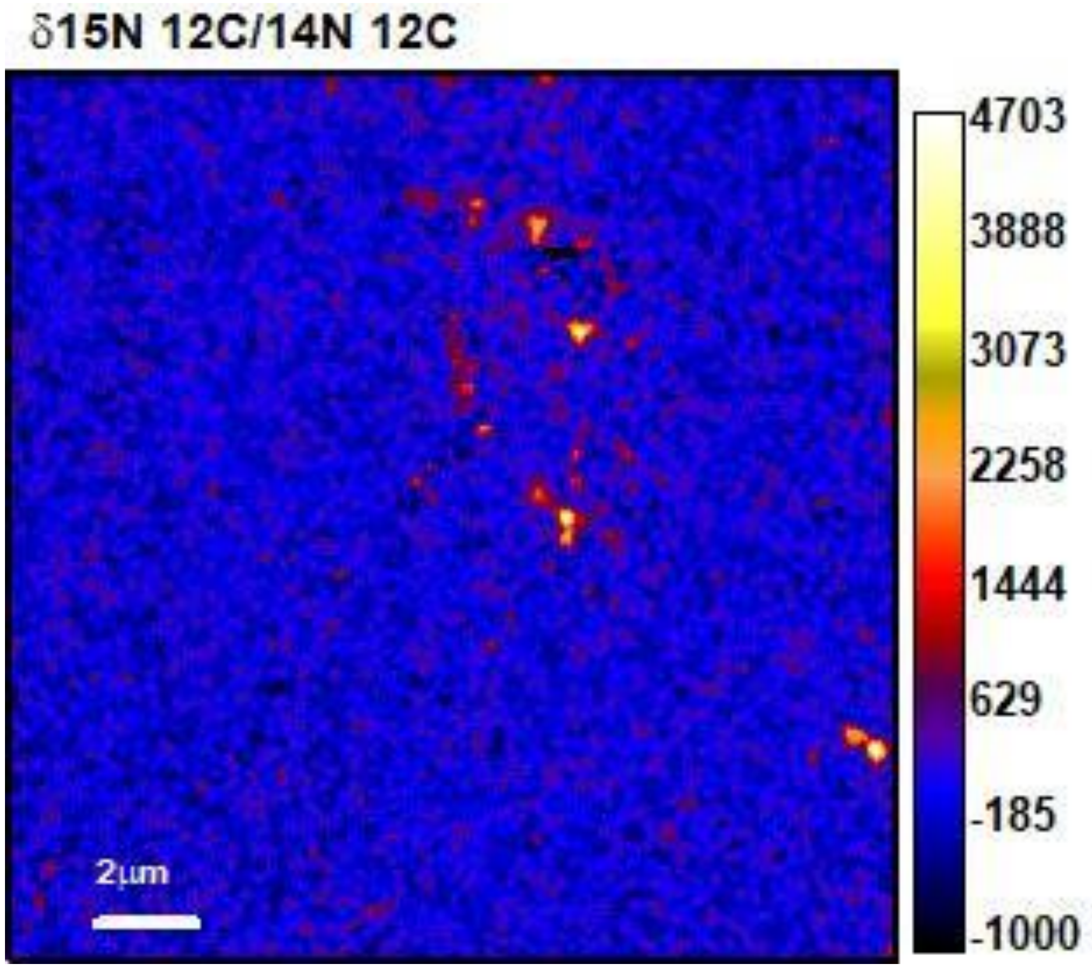

Im-8, D1-4,18x18, 4 layers.

Not labelled in 13C and weird in 15N

Sample LO4

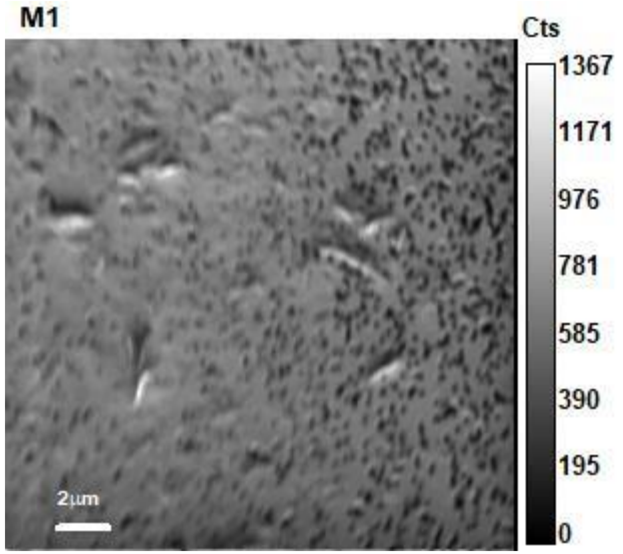

Im-9, D1-4,20x20, 4 layers.

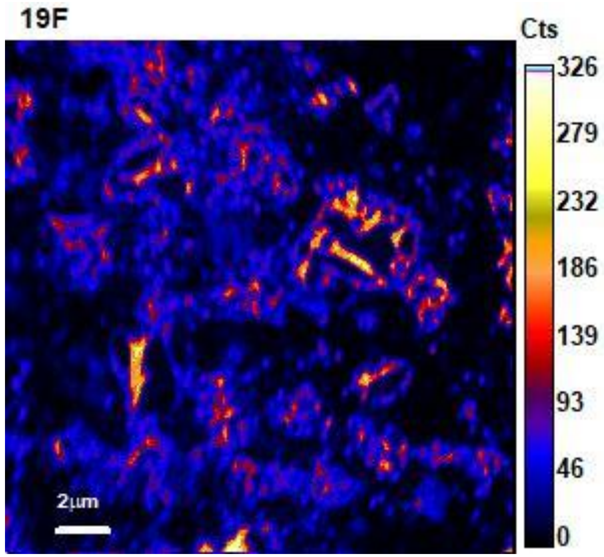

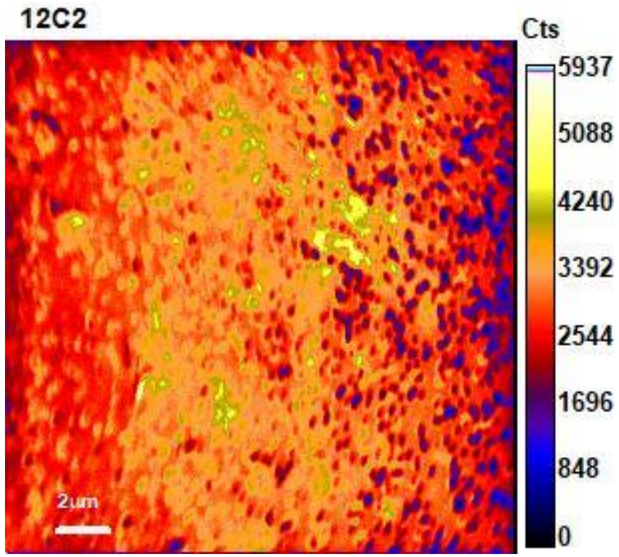

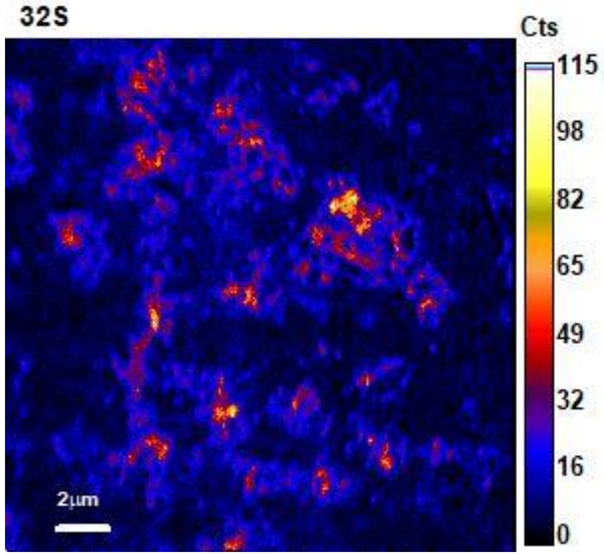

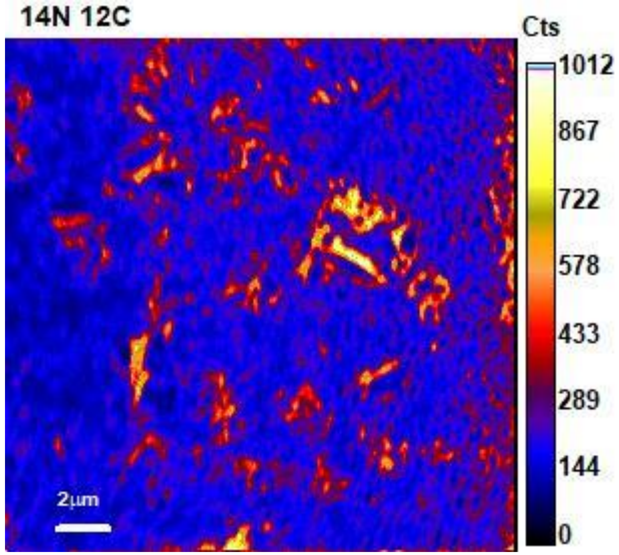

Sample LO4

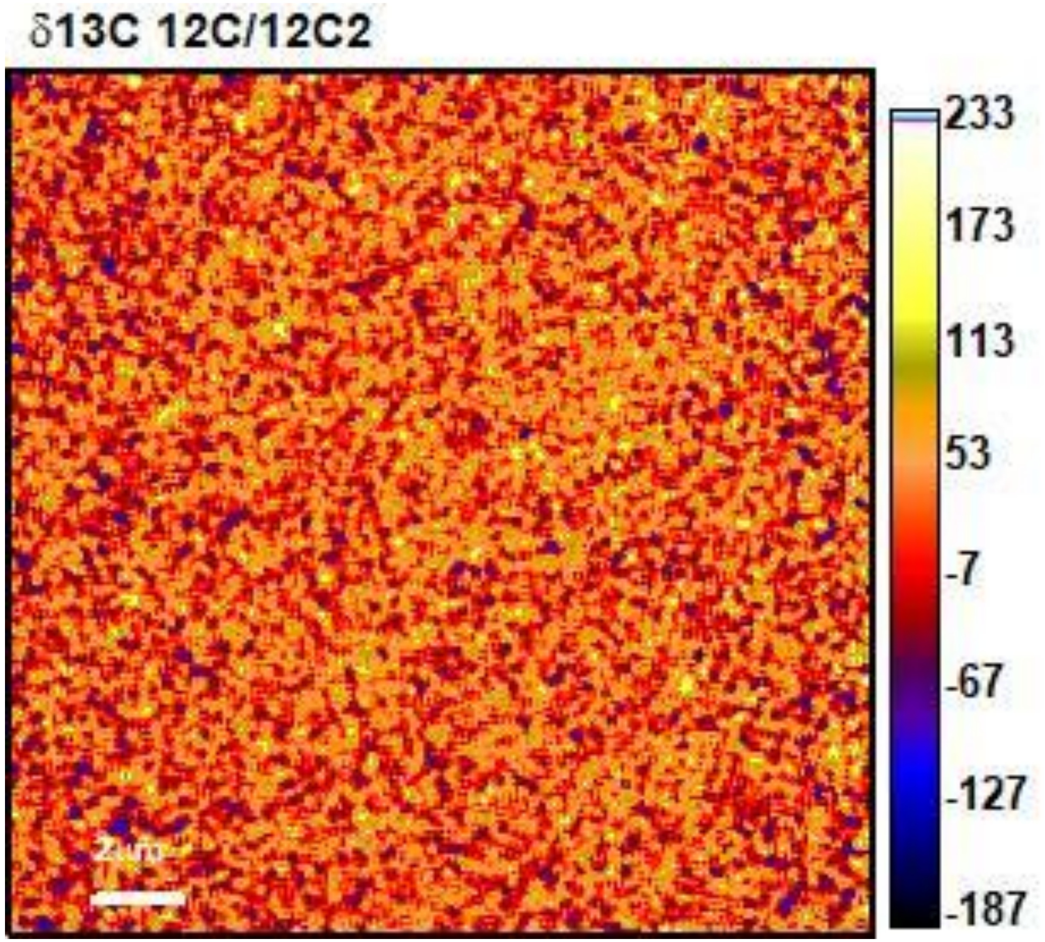

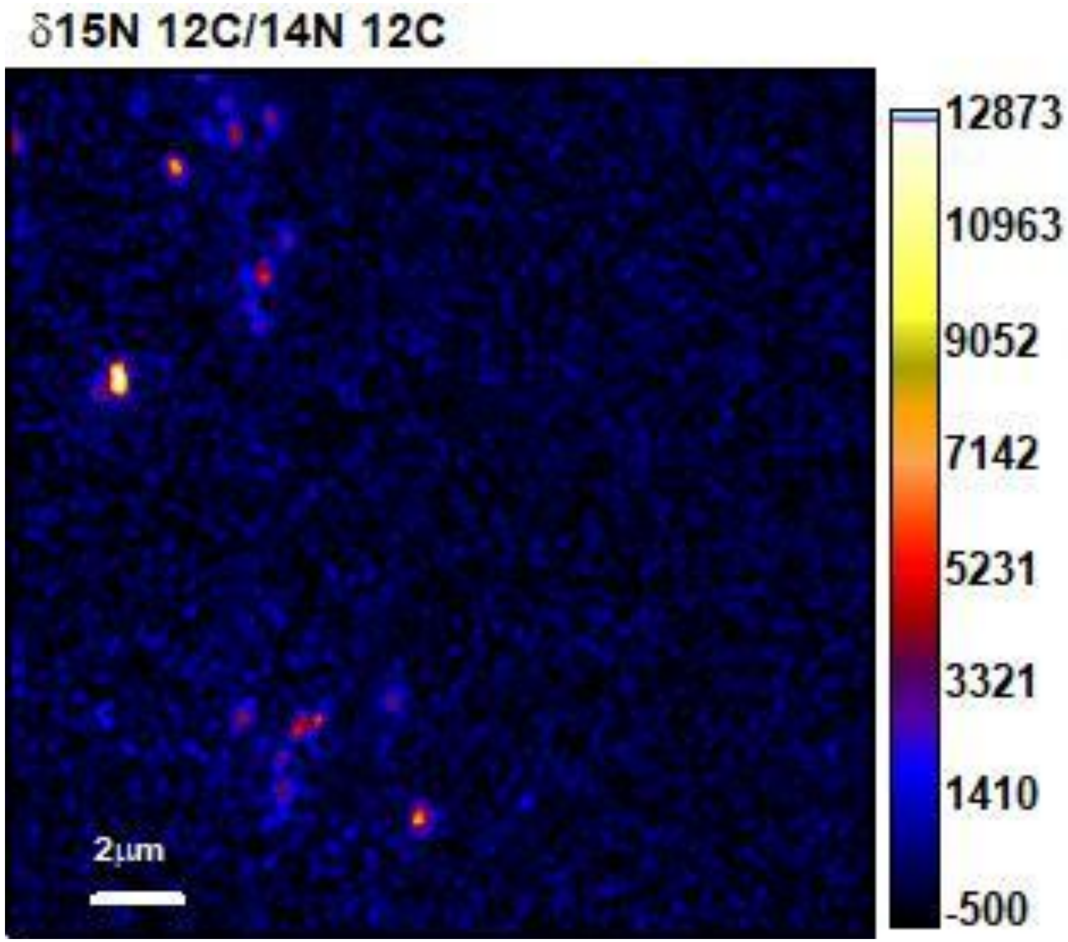

Im-9, D1-4,20x20, 4 layers.

Not labelled in 13C and weird in 15N

Sample LO2

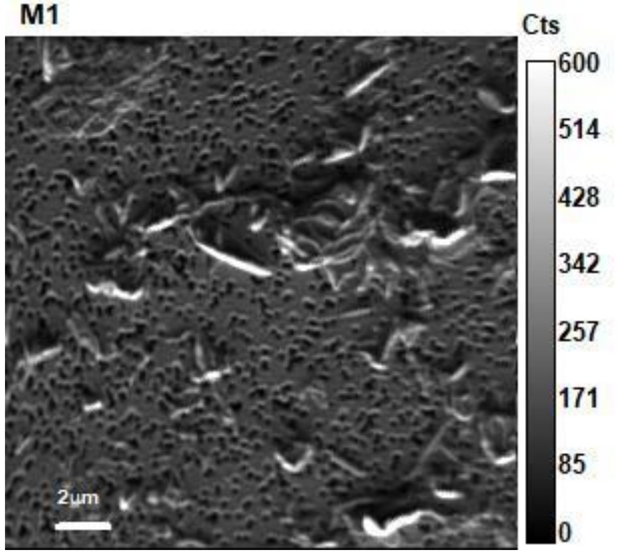

Im-10, D1-4,20x20, 4 layers.

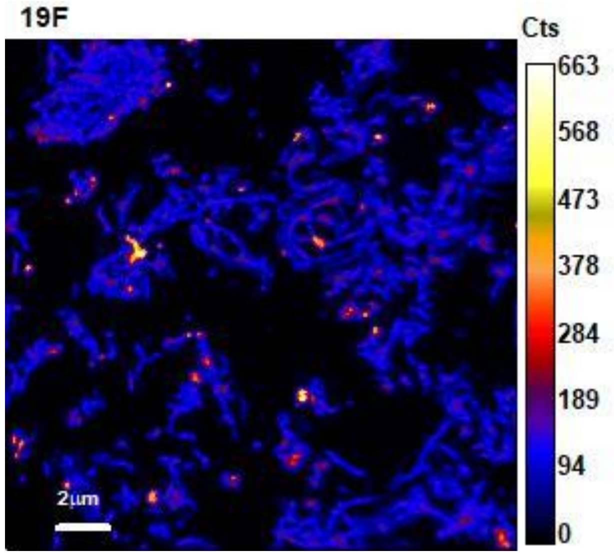

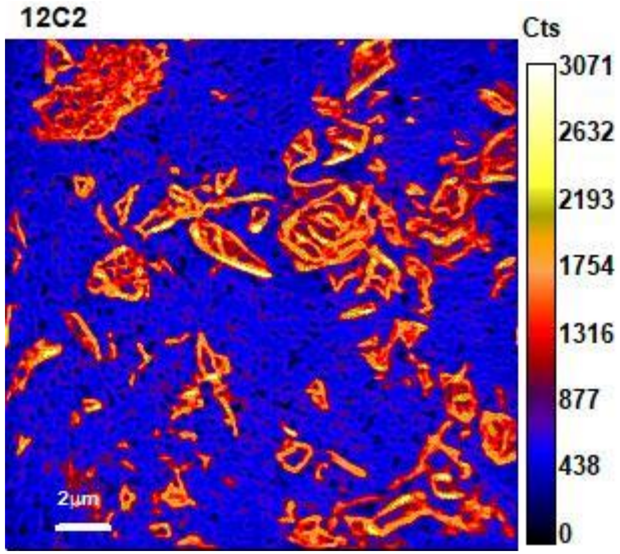

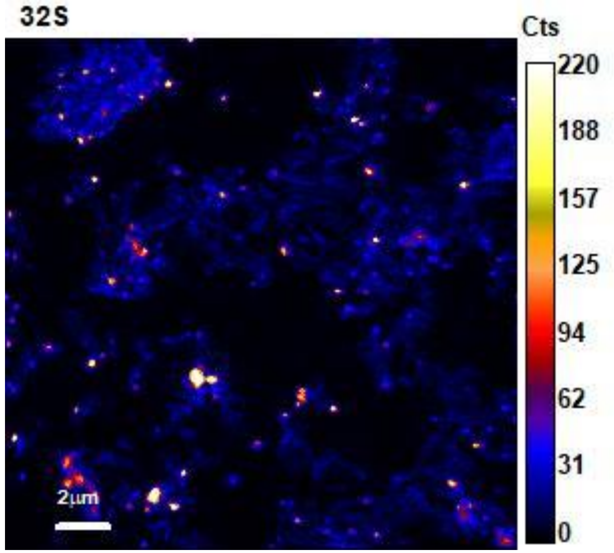

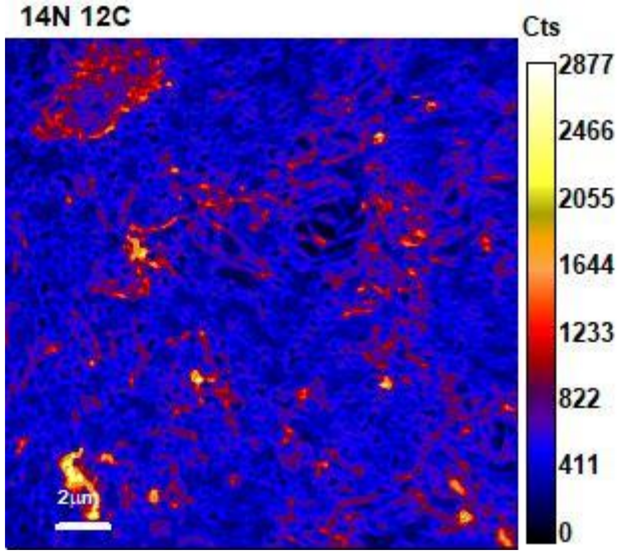

Sample LO2

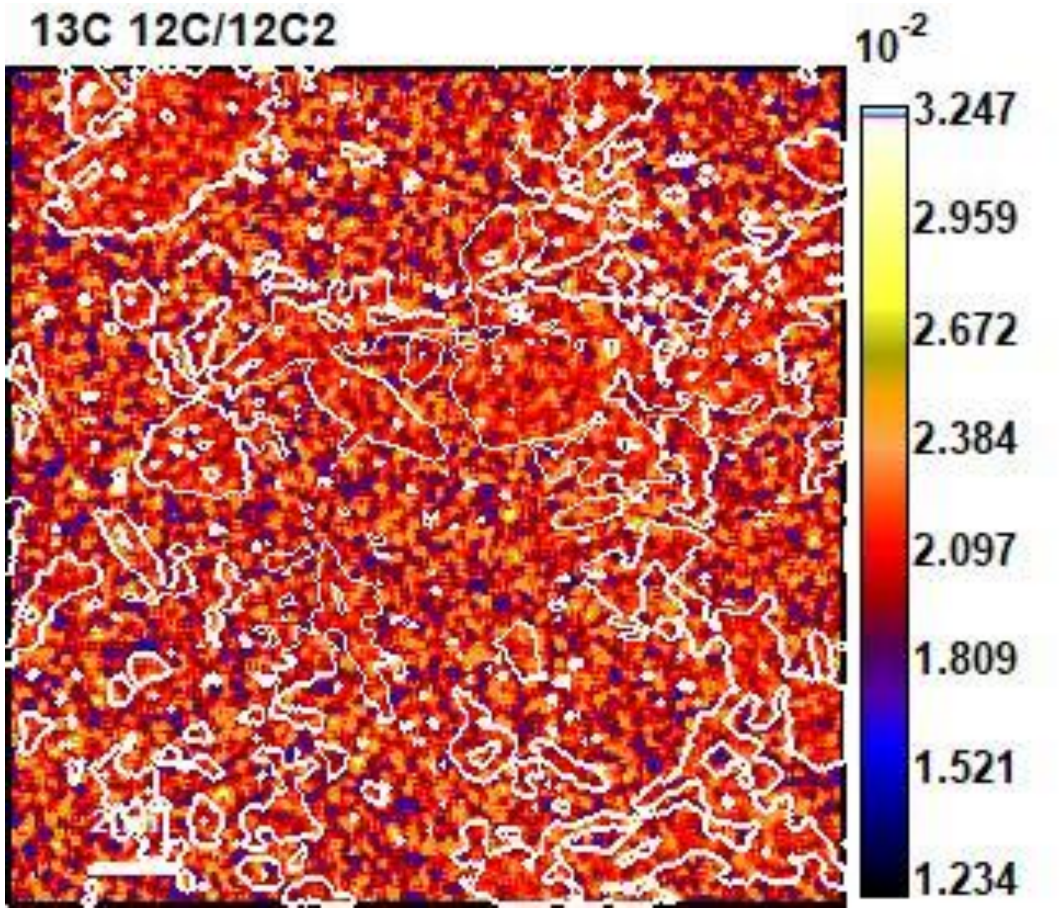

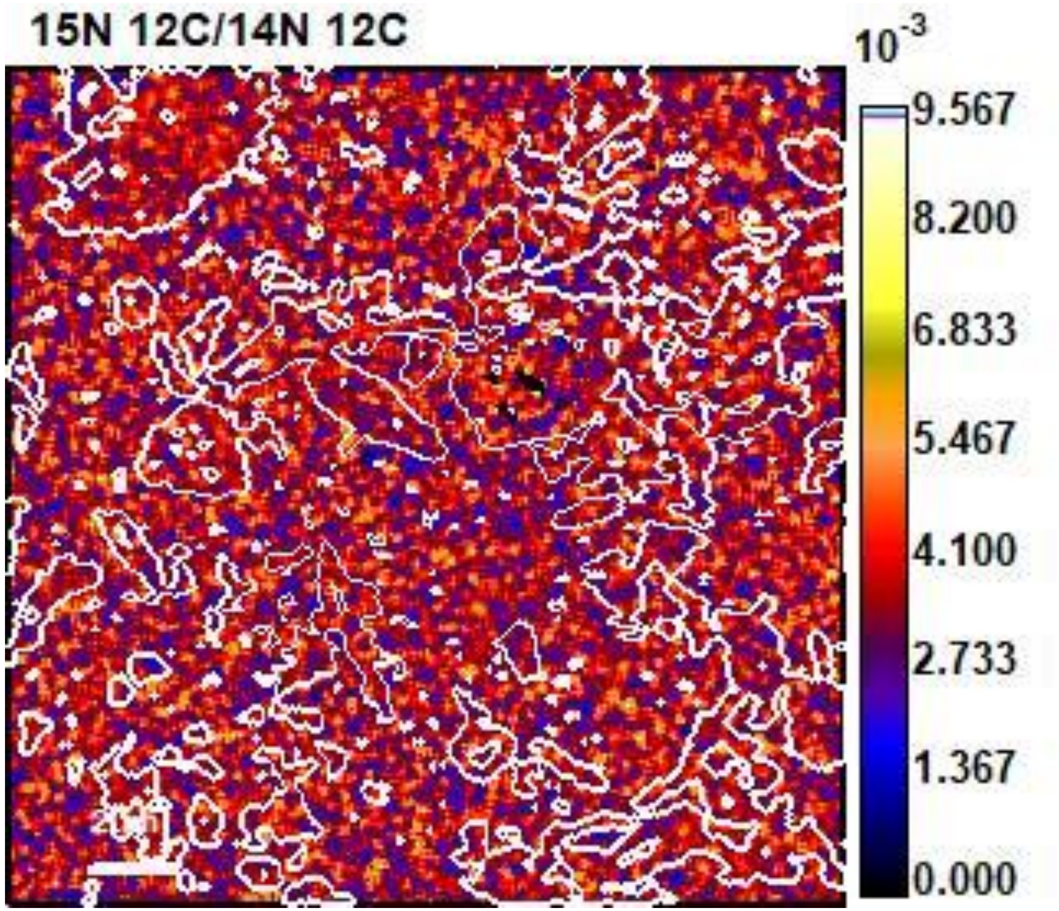

Im-10, D1-4,20x20, 4 layers.

Not labelled in 13C and in 15N

Sample LO2

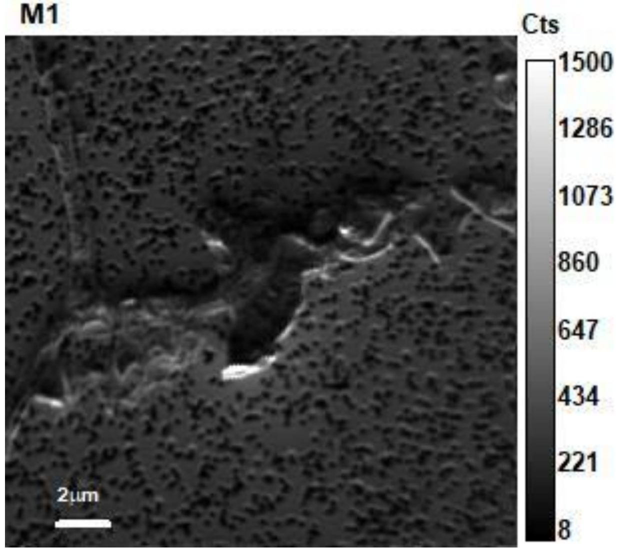

Im-11, D1-4,20x20, 4 layers.

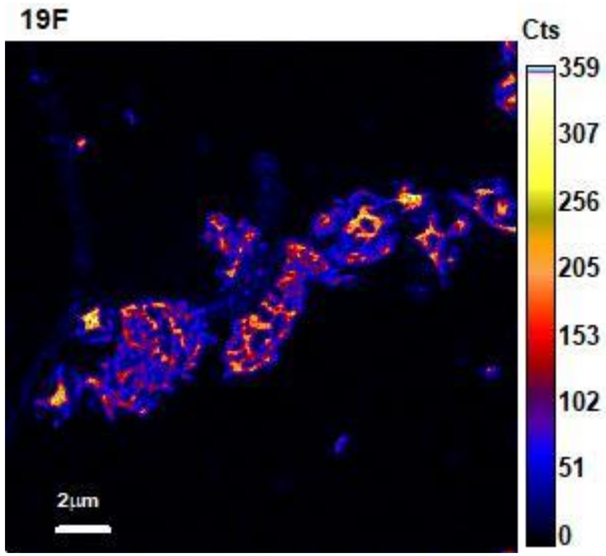

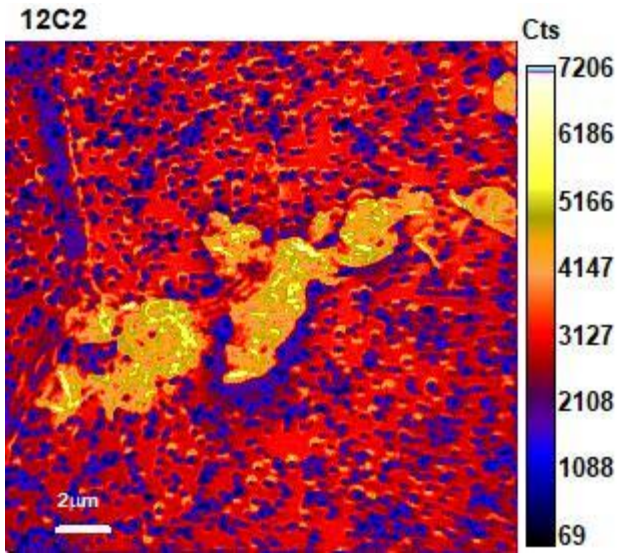

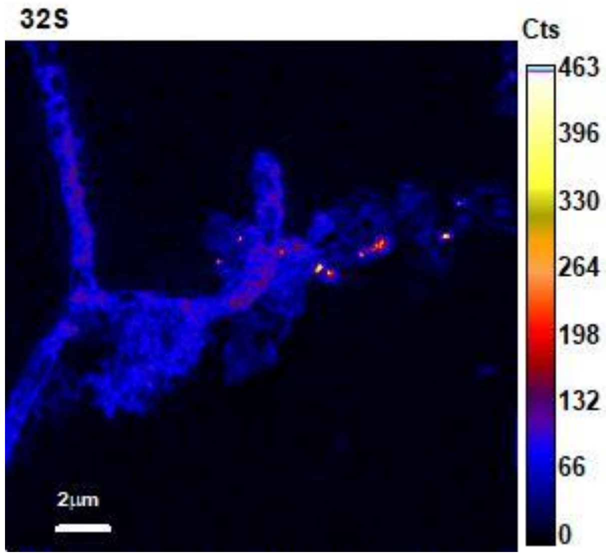

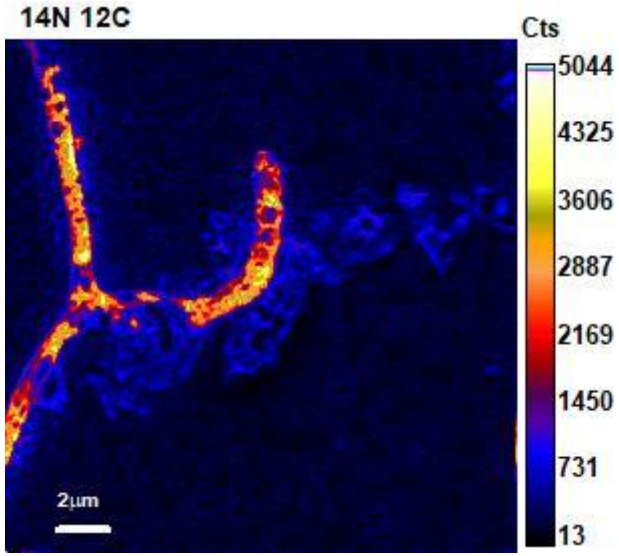

Sample LO2

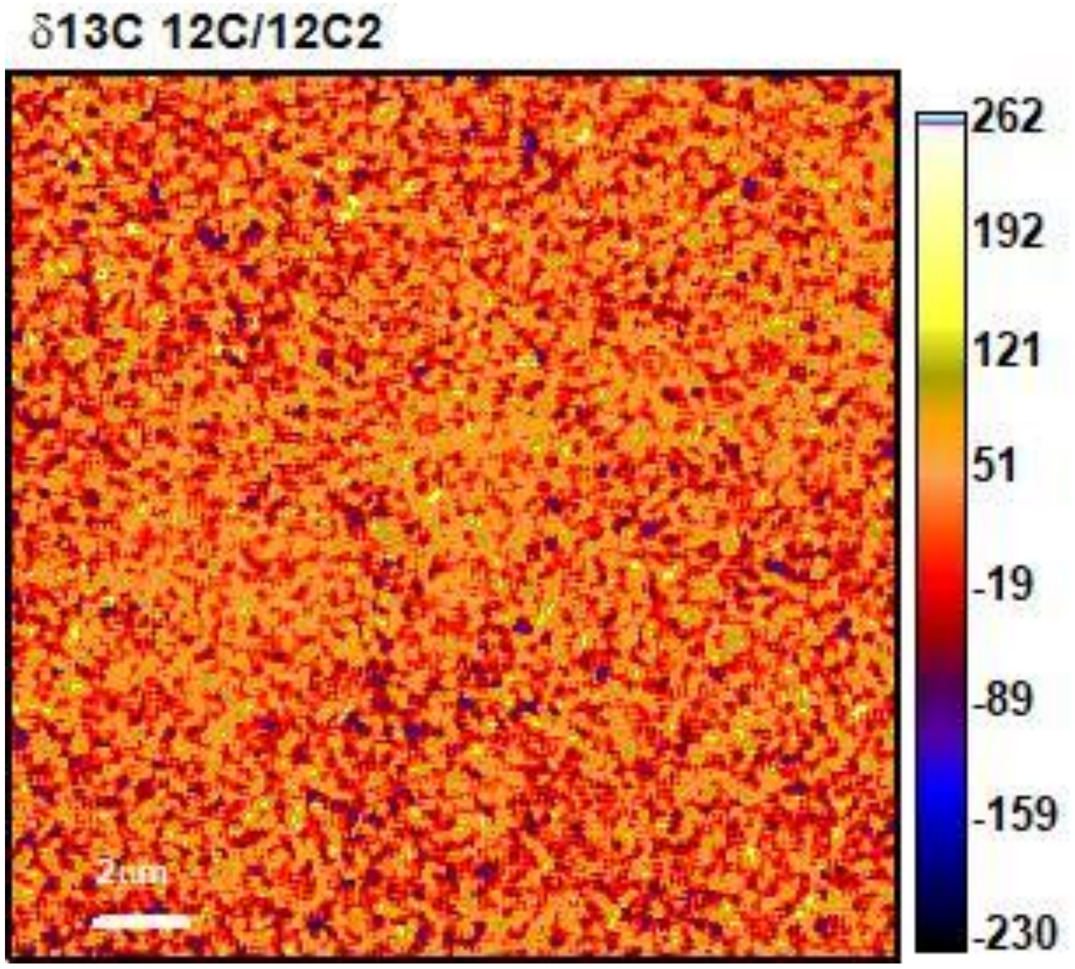

Im-11, D1-4,20x20, 4 layers.

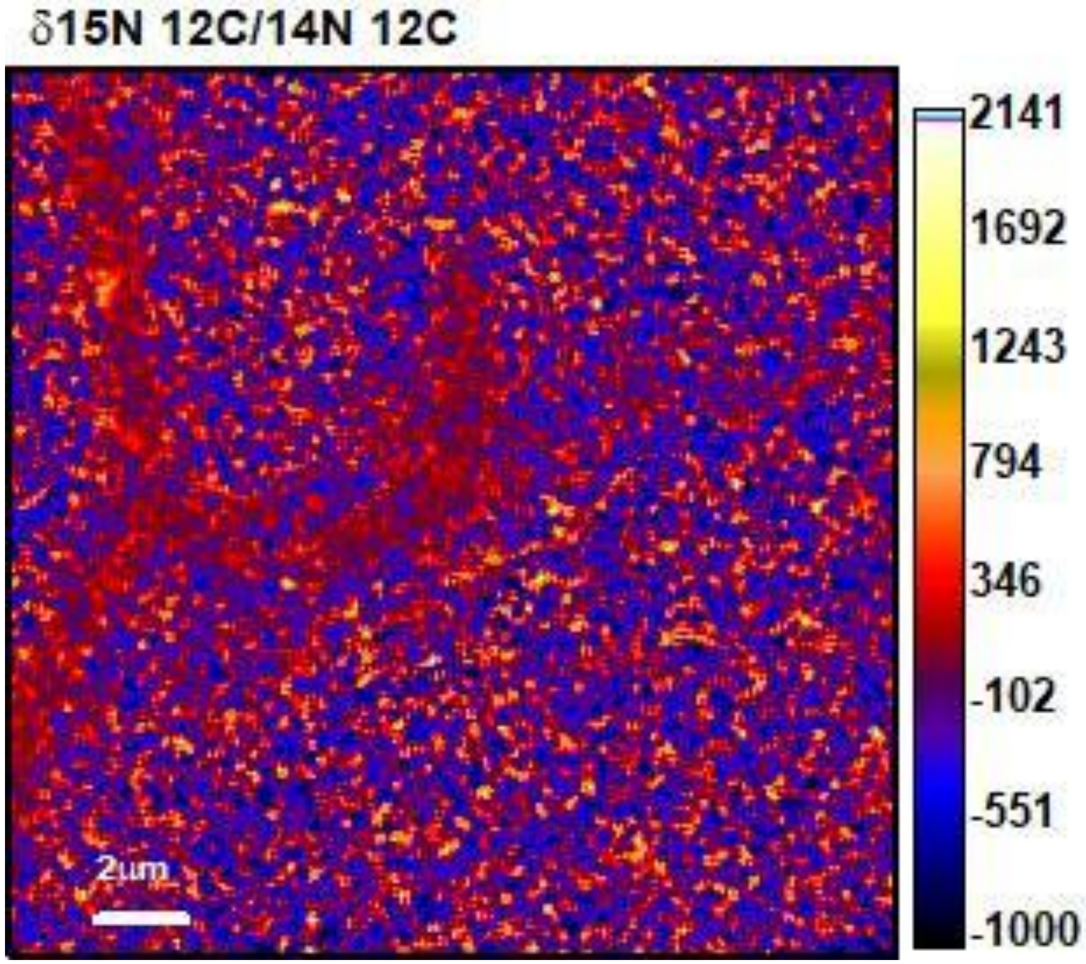

Not labelled in 12C and weird in 15N

Control C1

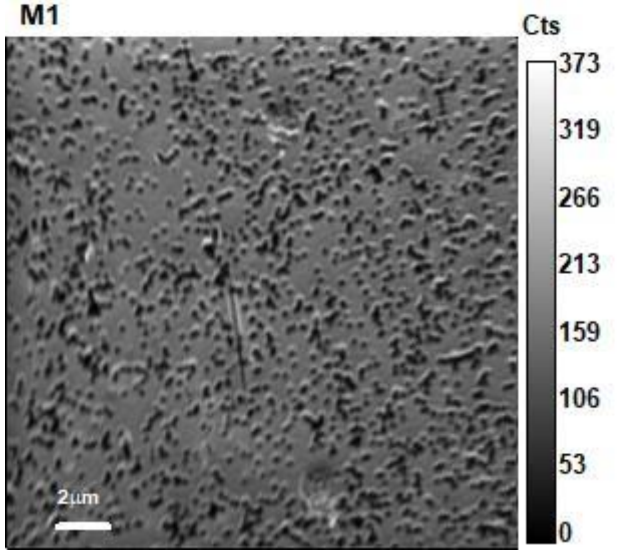

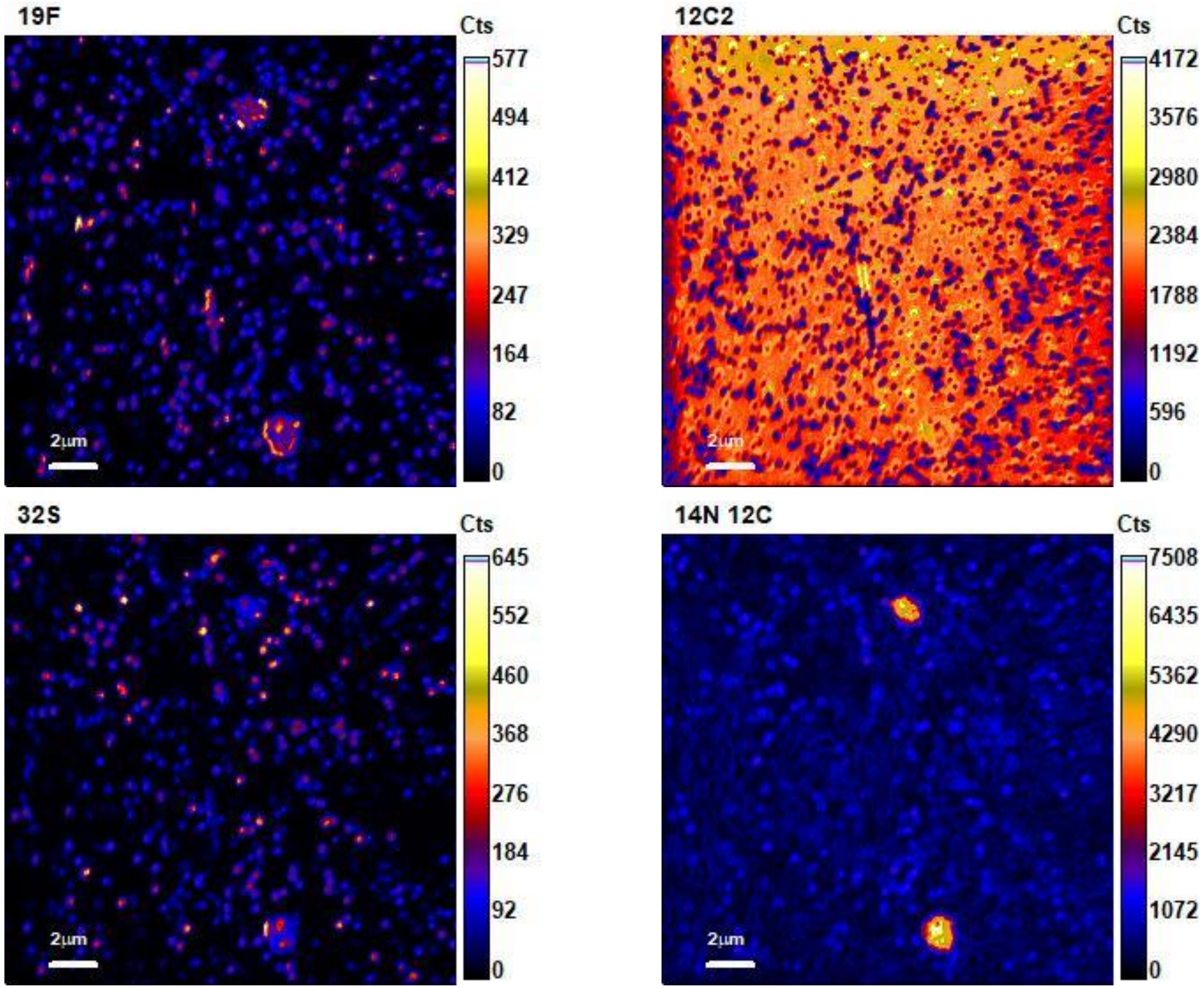

Im-12, D1-4,20x20, 4 layers.

Control C1

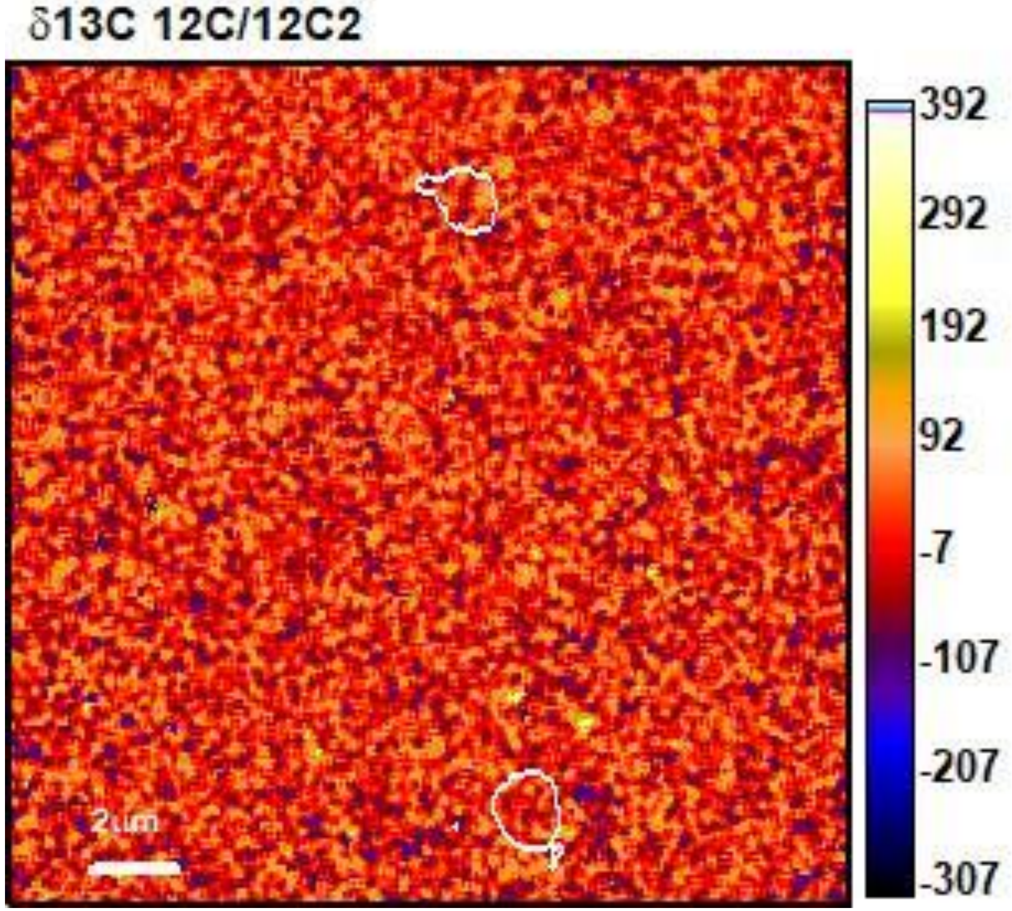

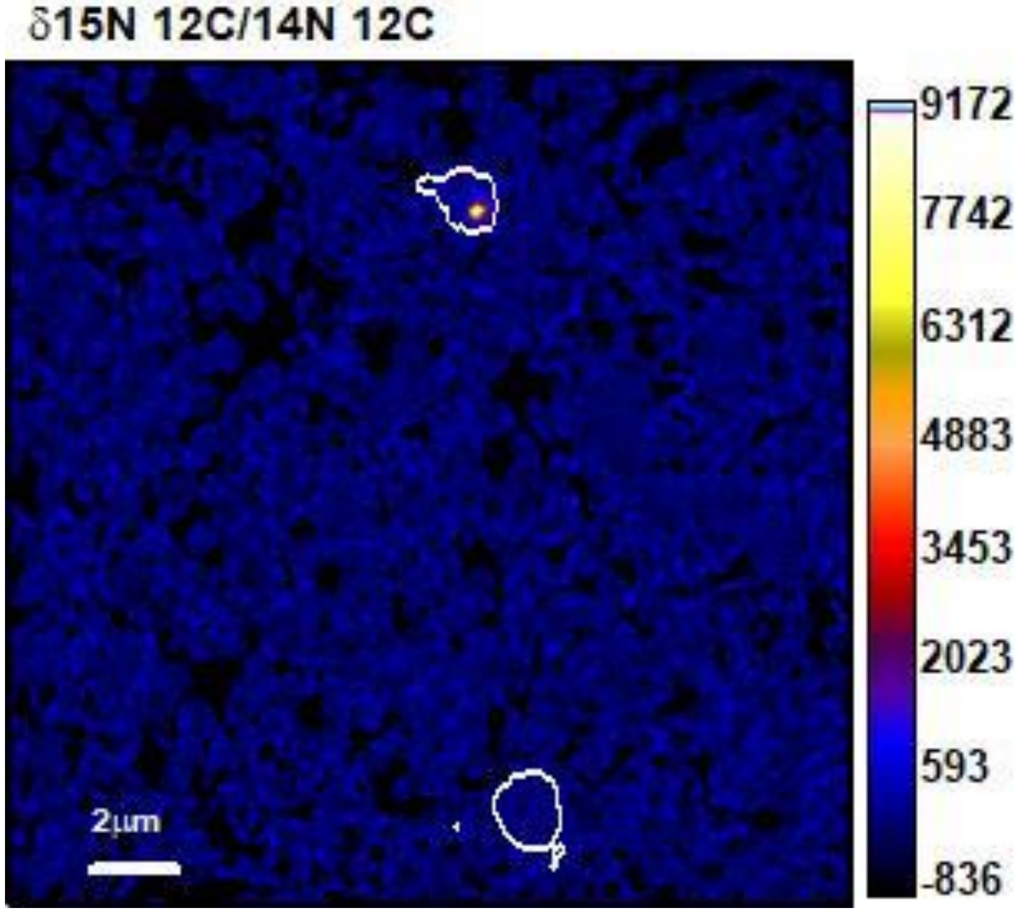

Im-12, D1-4,20x20, 4 layers.

Enrichment in 15N in one of the ROI
Nothing in 13C

Sample DA3

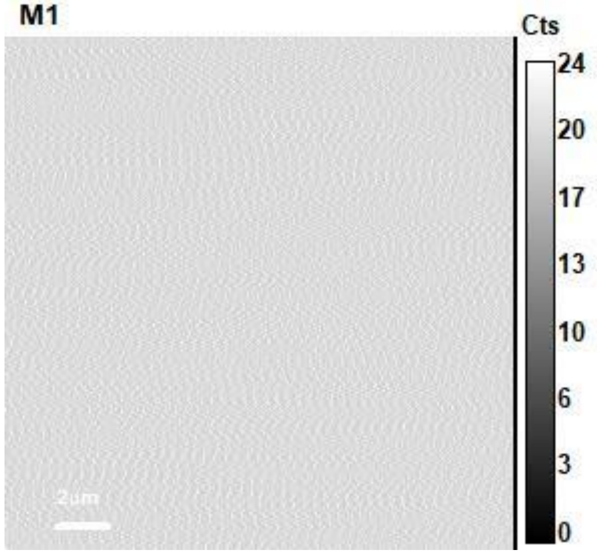

Im-13, D1-4,20x20, 4 layers.

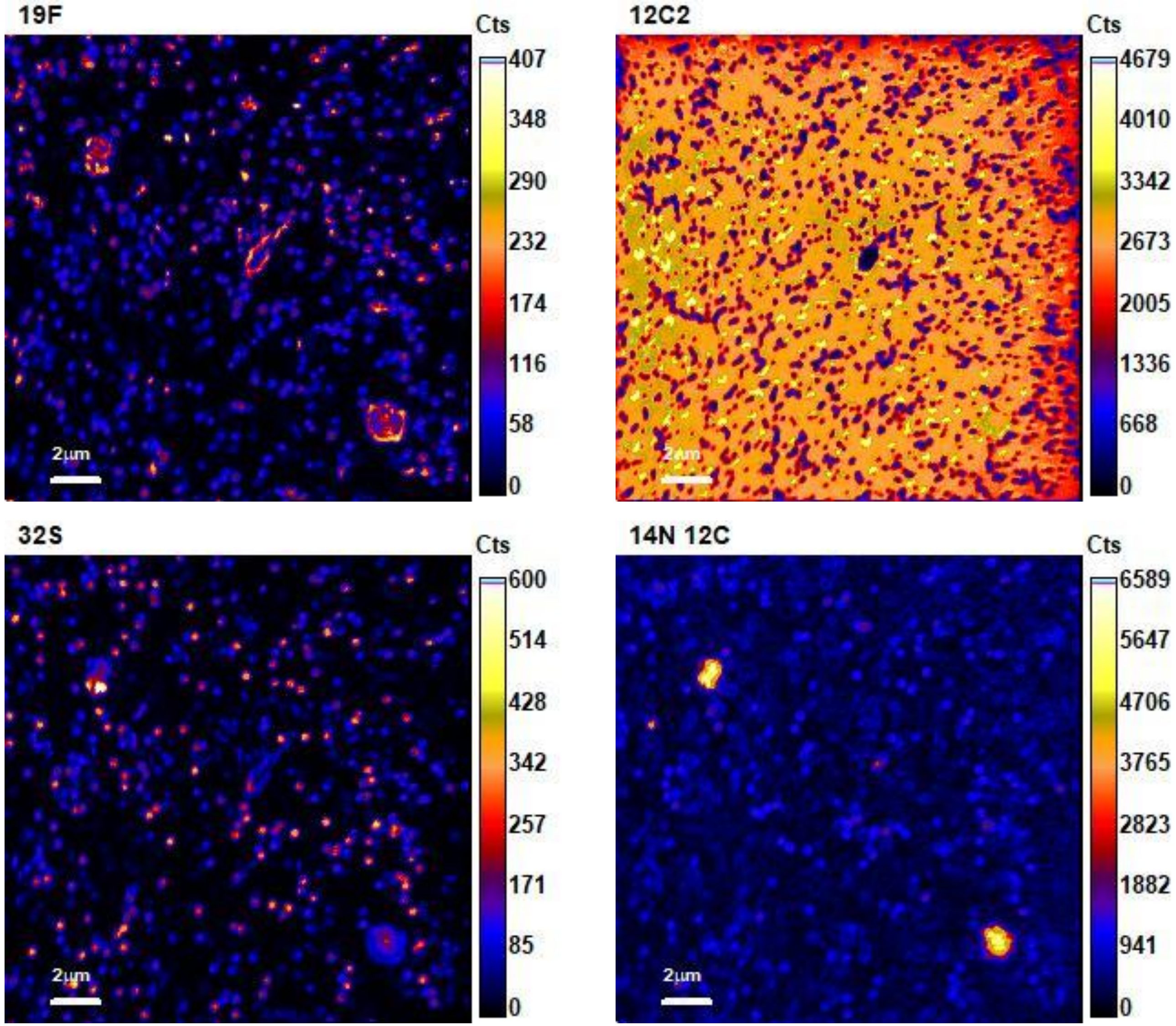

Sample DA3

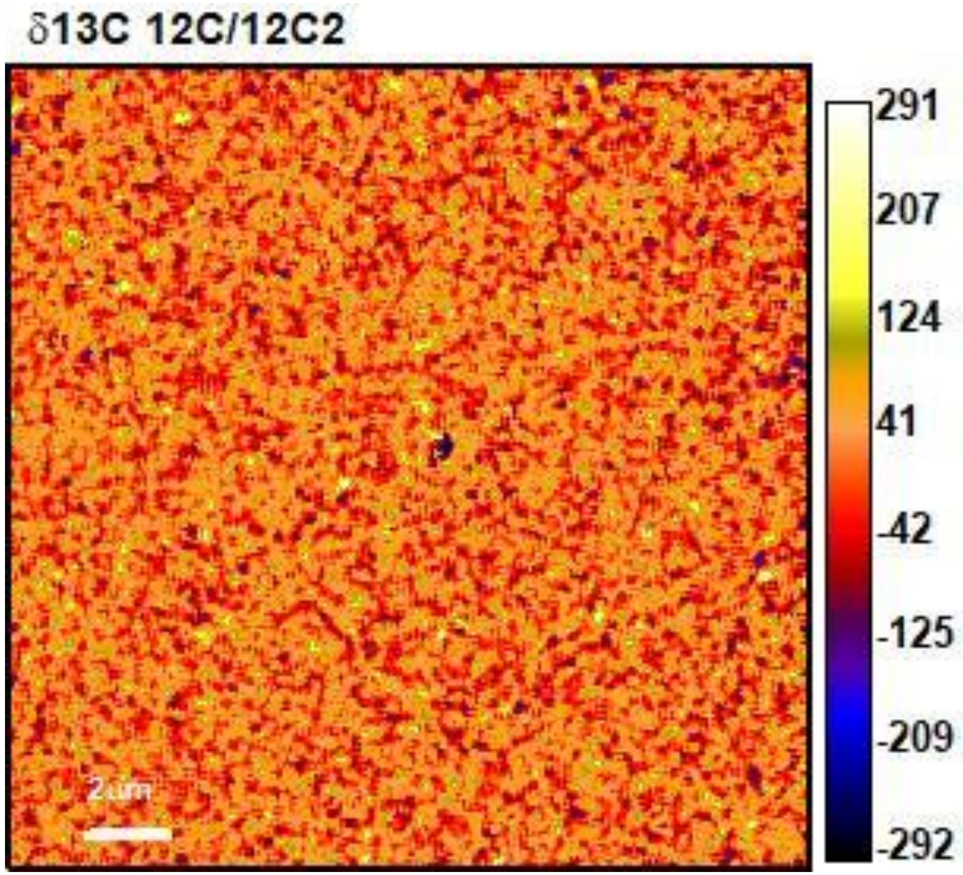

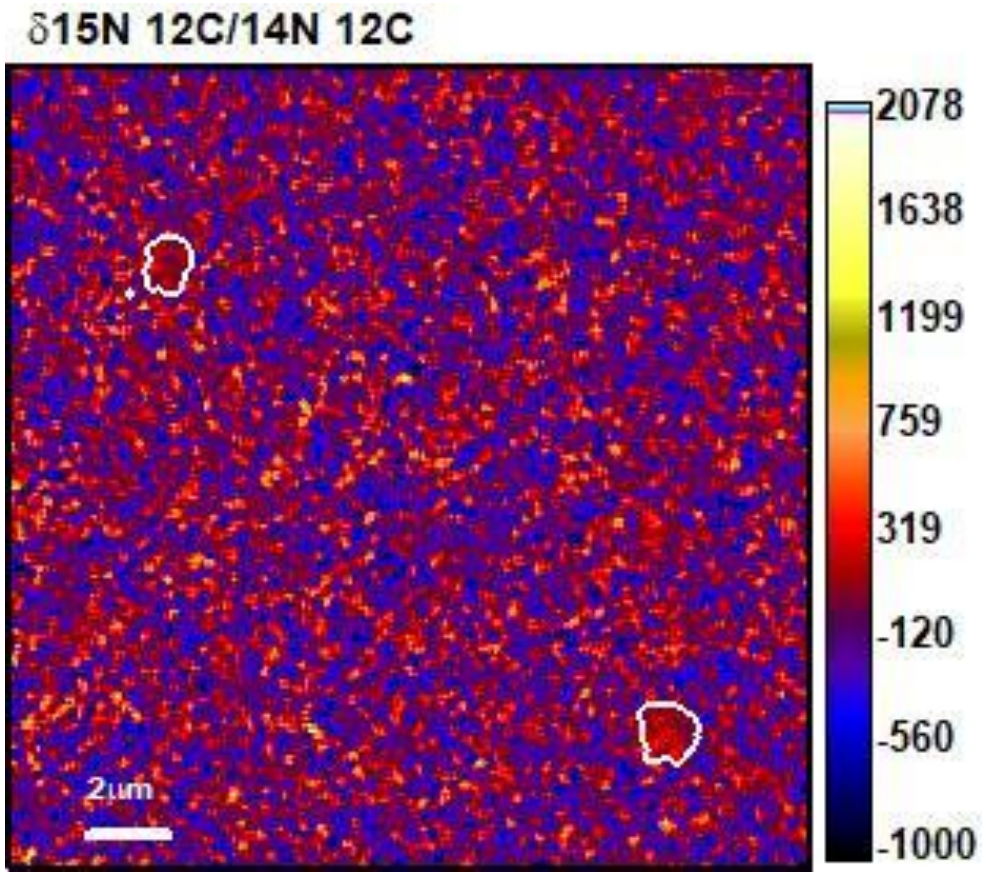

Im-2, D1-4,15x15, 4 layers.

Nothing in 13C but maybe in 15N (150 permil)

Sample DA3

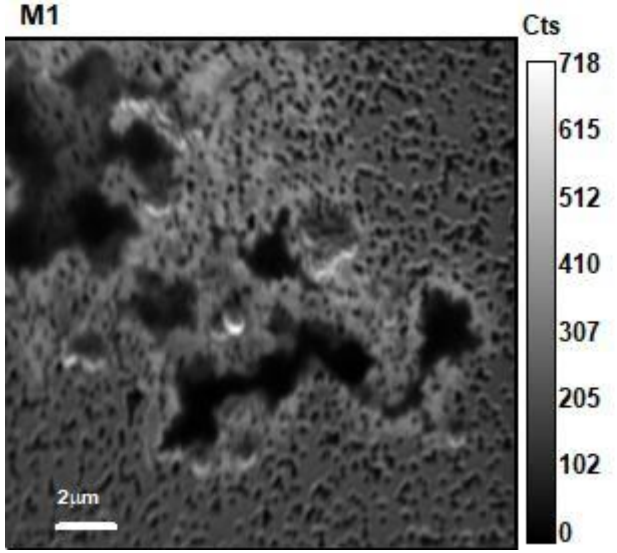

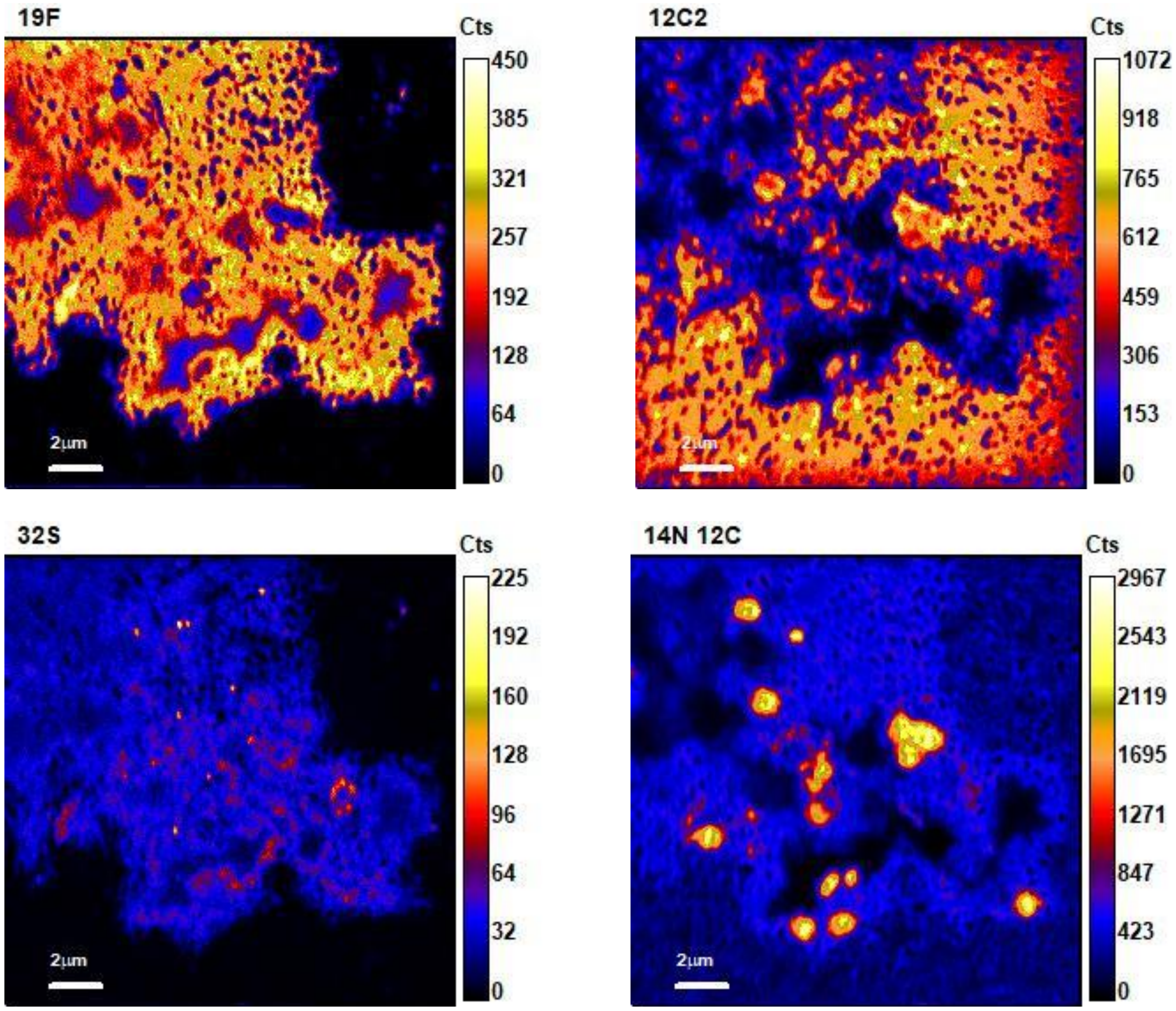

Im-14, D1-4,18x18, 4 layers.

Sample DA3

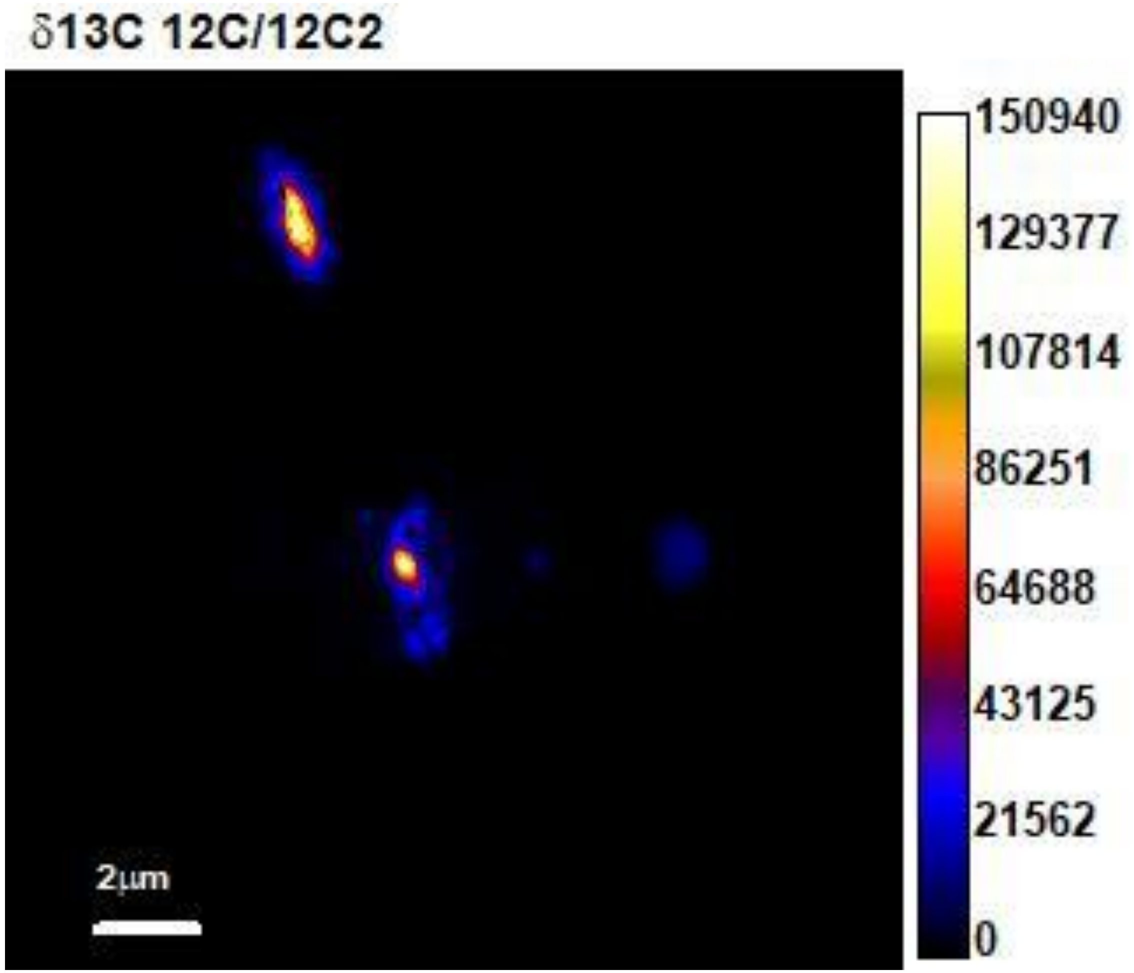

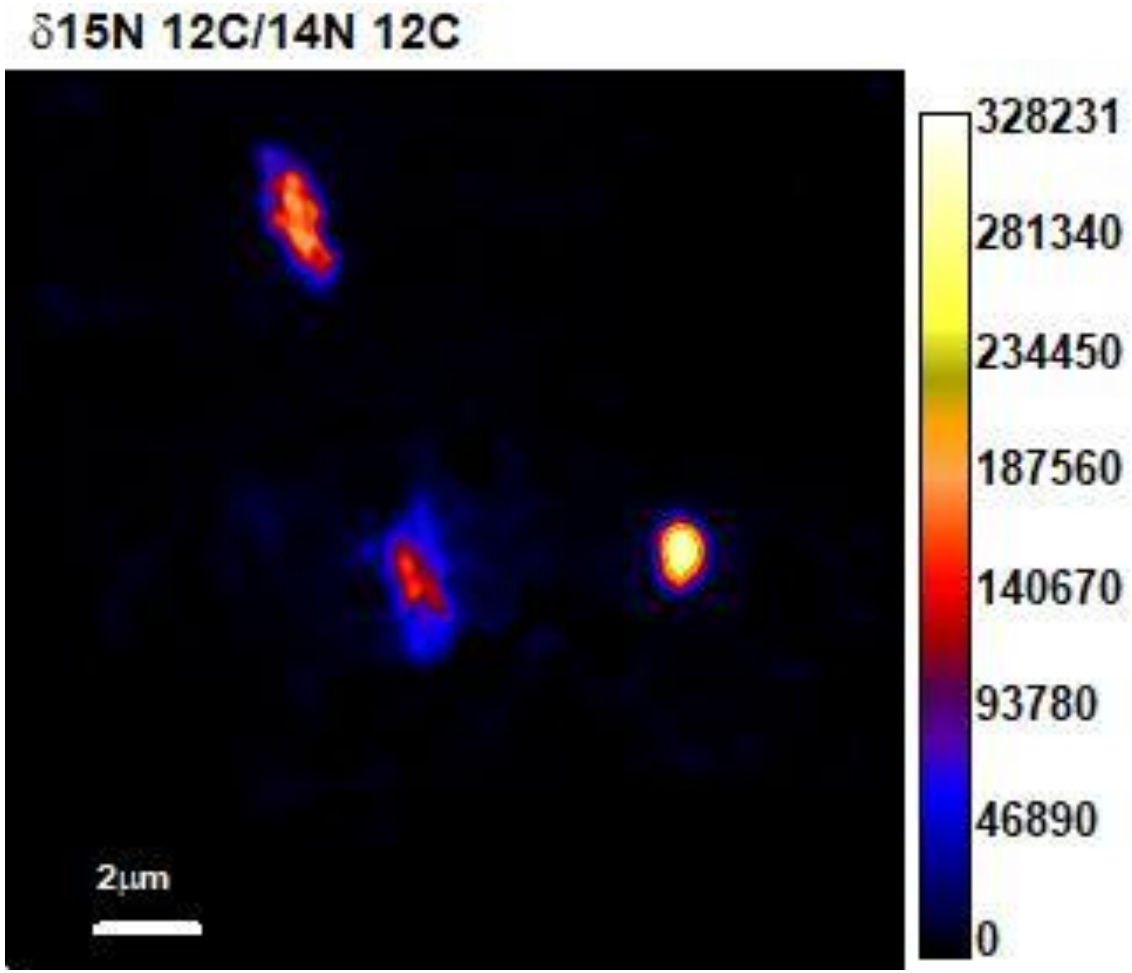

Im-14, D1-4,18x18, 4 layers.

Sample DA3

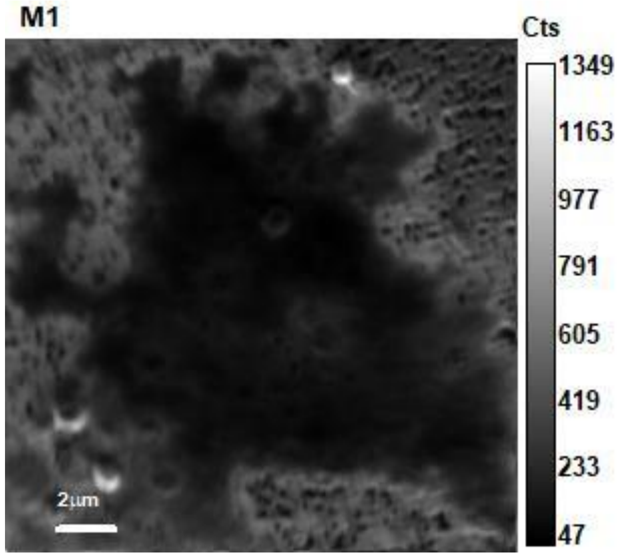

Im-15, D1-4,18x18, 4 layers.

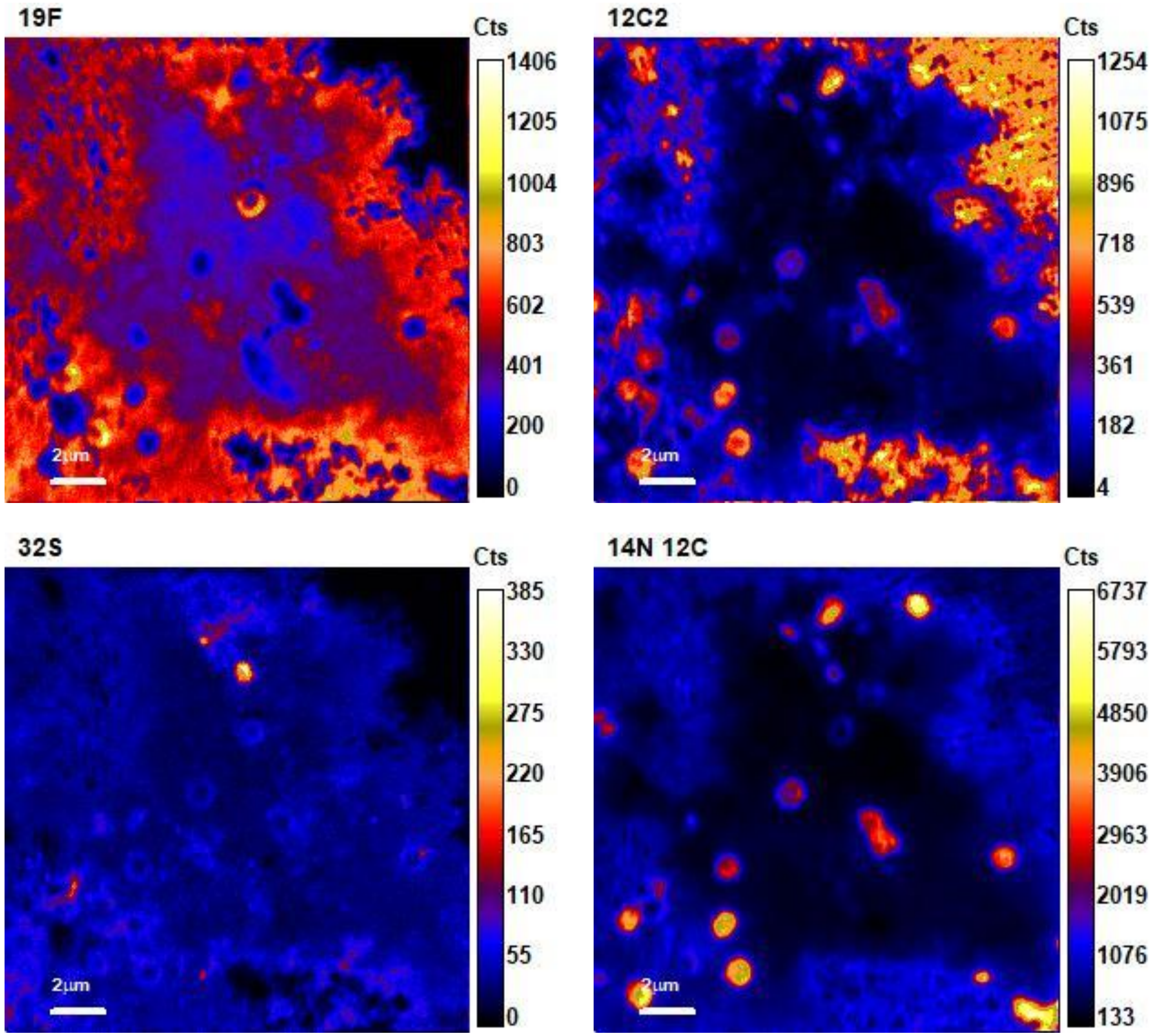

Sample DA3

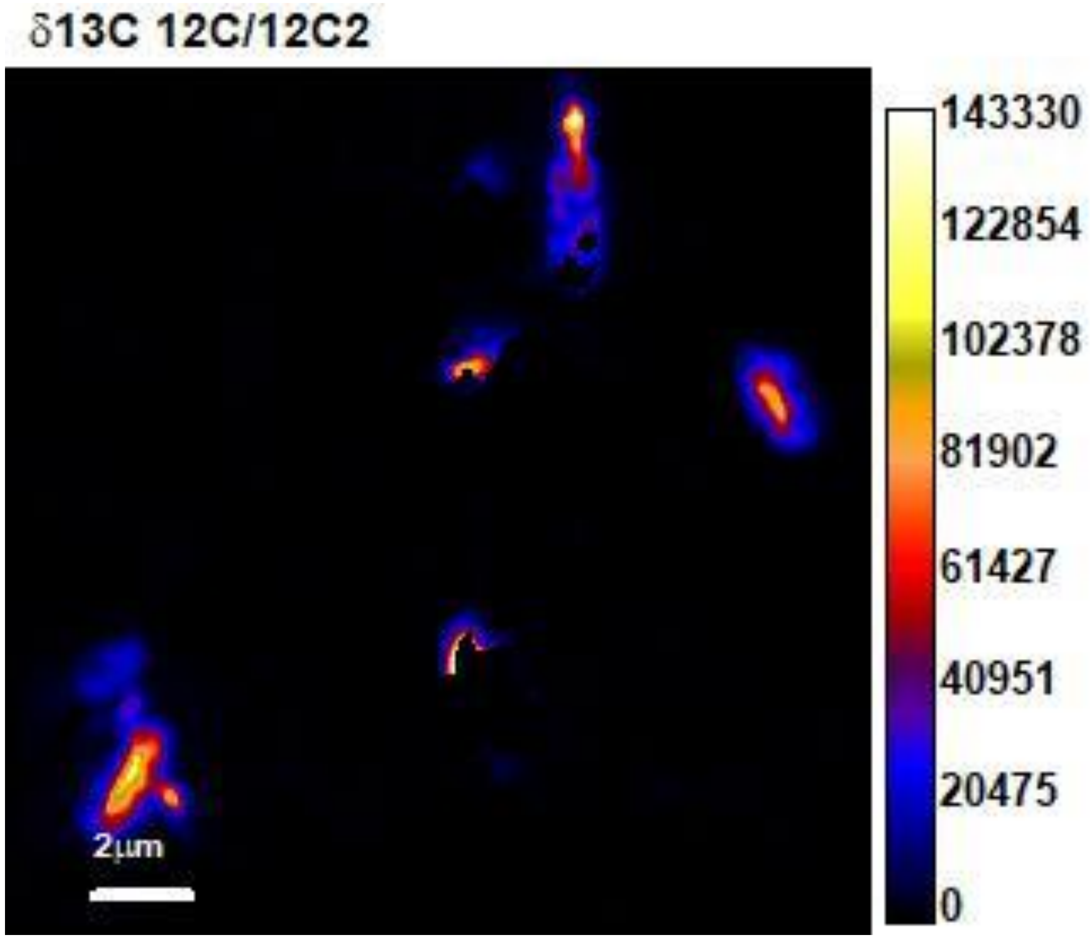

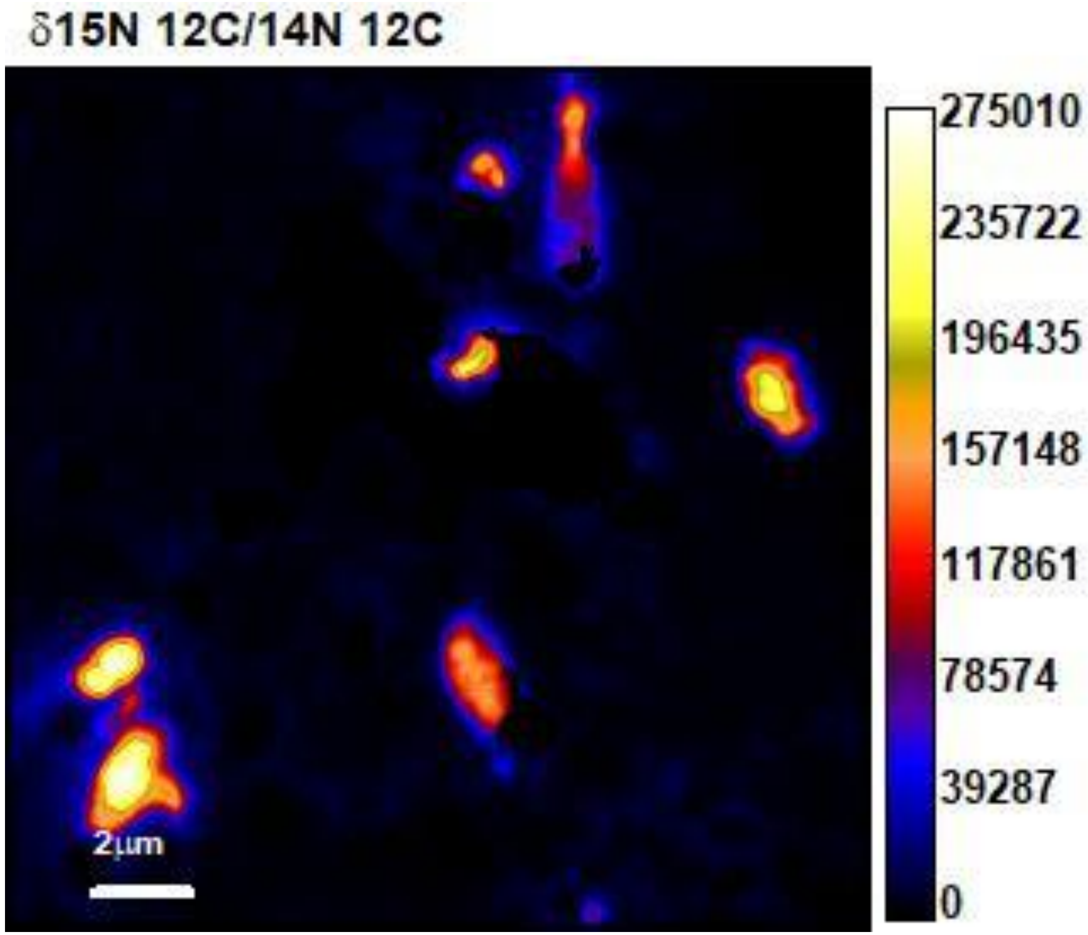

Im-15, D1-4,18x18, 4 layers.

Control C4

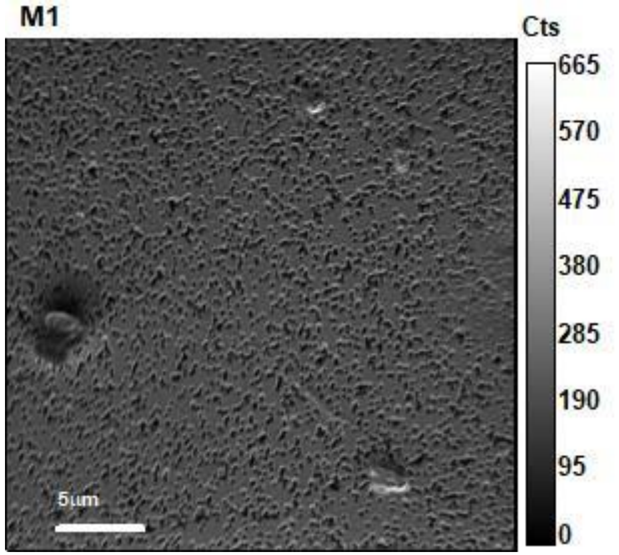

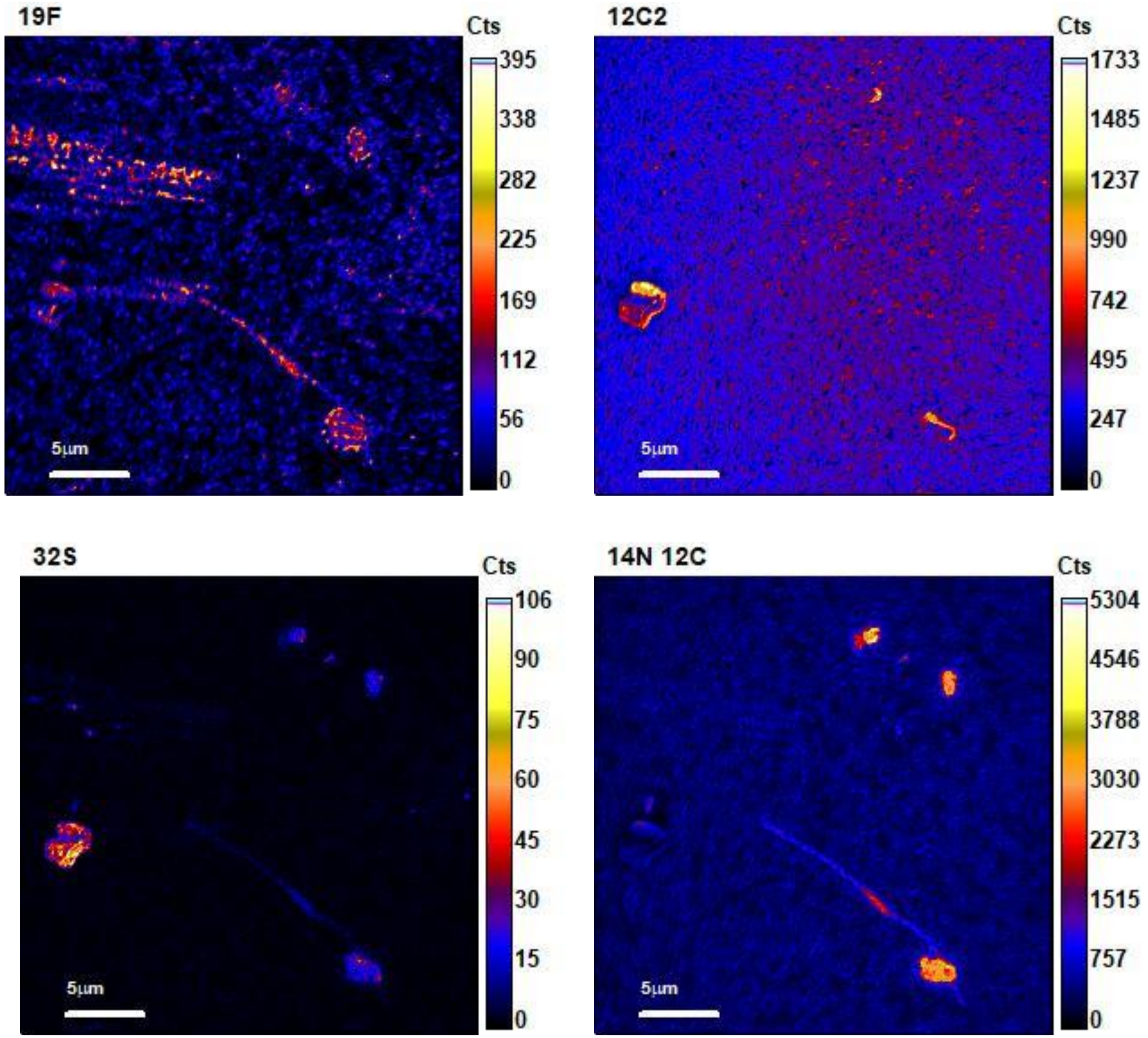

Im-16, D1-4,30x30, 4 layers.

Control C4

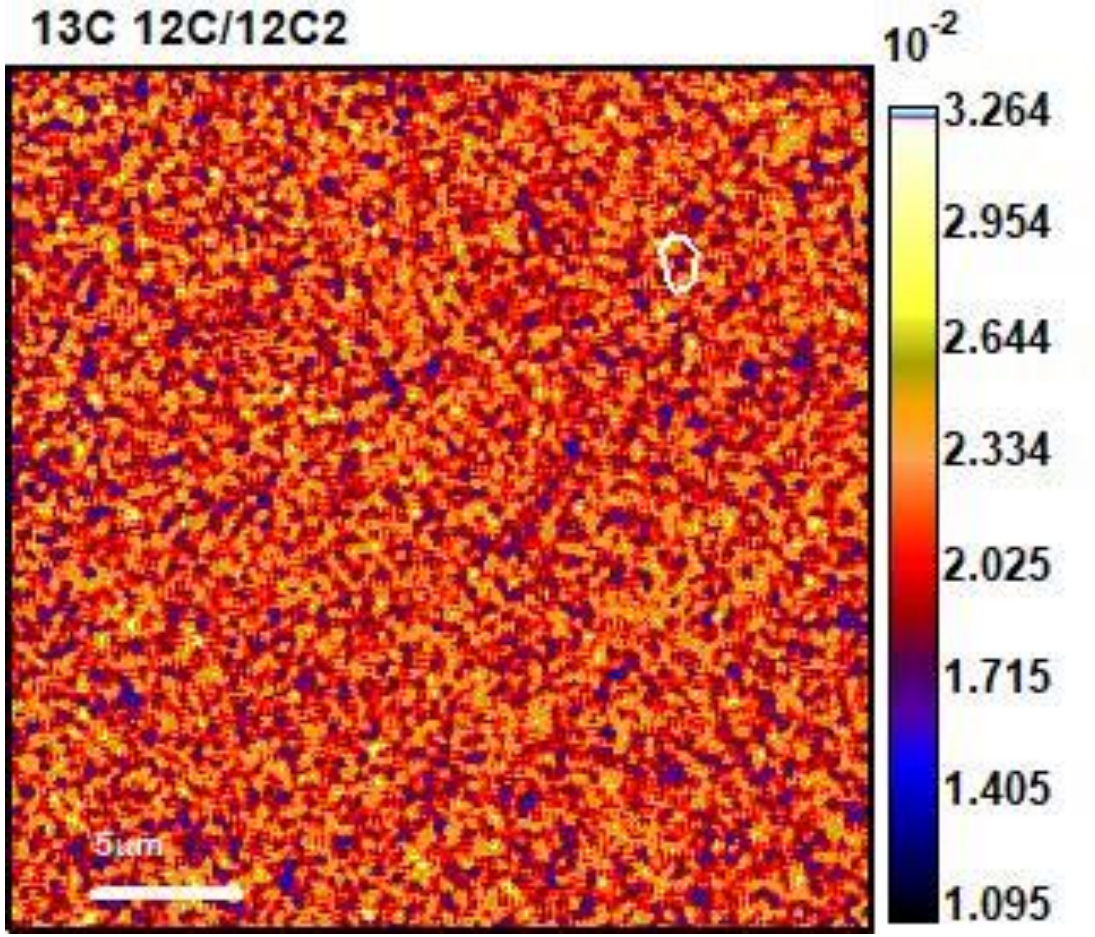

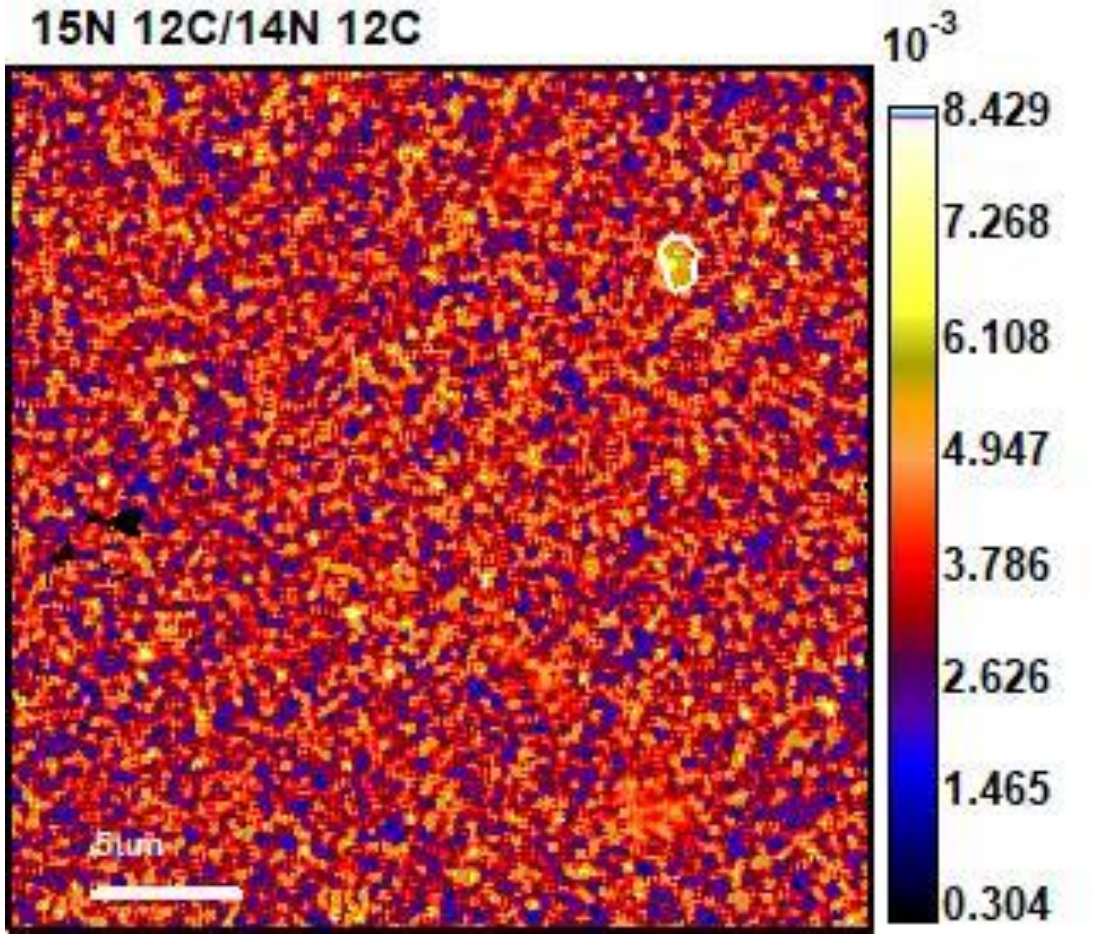

Im-16, D1-4,30x30, 4 layers.

No label in 13 BUT one ara enriched in 15N
D-15N12C/14N12C~495±35

Control C1: nothing in 13, possible enrichment in 15N
Control C4: nothing in 13, possible enrichment in 15N
DA-1: enriched in 13C and 15N
DA-2: enriched in 13C and 15N
DA-3: enriched in 13C and 15N
LO-2: nothing in 13, baseline high in 15N
LO-4: nothing in 13C, some hotspots in 15N
