# Supplementary material for: Black Sea picocyanobacteria exploit ancestral traits for energy and chlorophyll production in dark anoxia
Source: ISME Commun. 2026 Jul 17;6(1):ycag159. doi: 10.1093/ismeco/ycag159 (PMC13588713; doi:10.1093/ismeco/ycag159)
Supplement: Supplementary file 1 [file web_material_ycag159.zip › Suppl-Info rev.pdf]

SUPPLEMENTARY INFORMATION FILE

Black Sea picocyanobacterium exploits ancestral traits for energy and chlorophyll production in dark anoxia

Maciej Bartosiewicz[1,2]*, Pedro J. Cabello-Yeves[3,4,5]*, Ester M. Eckert[6], Andrea Di Cesare[6], Richard J. Puxty[5], Anna Przytulska[1], Helena Osterholz[7], Anders Meibom[8,9], Stephane Escrig[8], Nina Dzhembekova[10], Jakob Zopfi[1], Moritz F. Lehmann[1] and Cristiana Callieri[6©]

[1] Department of Environmental Sciences, University of Basel, Basel, Switzerland
[2] Institute of Geophysics, Polish Academy of Sciences, Warsaw, Poland
[3] Evolutionary Genomics Group, Departamento de Producción Vegetal y Microbiología, Universidad Miguel, Hernández, San Juan de Alicante, Spain
[4] Cavanilles Institute of Biodiversity and Evolutionary Biology, University of Valencia, Valencia, Spain
[5] School of Life Sciences, University of Warwick, Coventry, UK
[6] National Research Council, Institute of Water Research, Verbania, Italy
[7] Marine Chemistry, Leibniz Institute for Baltic Sea Research Warnemünde, Rostock, Germany
[8] Laboratory for Biological Geochemistry, École Polytechnique Fédérale de Lausanne (EPFL), Lausanne, Switzerland
[9] Center for Advanced Surface Analysis, Institute of Earth Sciences, University of Lausanne, Lausanne, Switzerland
[10] Institute of Oceanology, Bulgarian Academy of Sciences, Varna, Bulgaria

*These authors contributed equally

** Corresponding author:

Prof. Dr. Cristiana Callieri
Senior Associate Researcher, CNR-IRSA Institute of Water Research
Largo Tonolli 50, 28922, Verbania, Italy
E mail cristiana.callieri@irsa.cnr.it

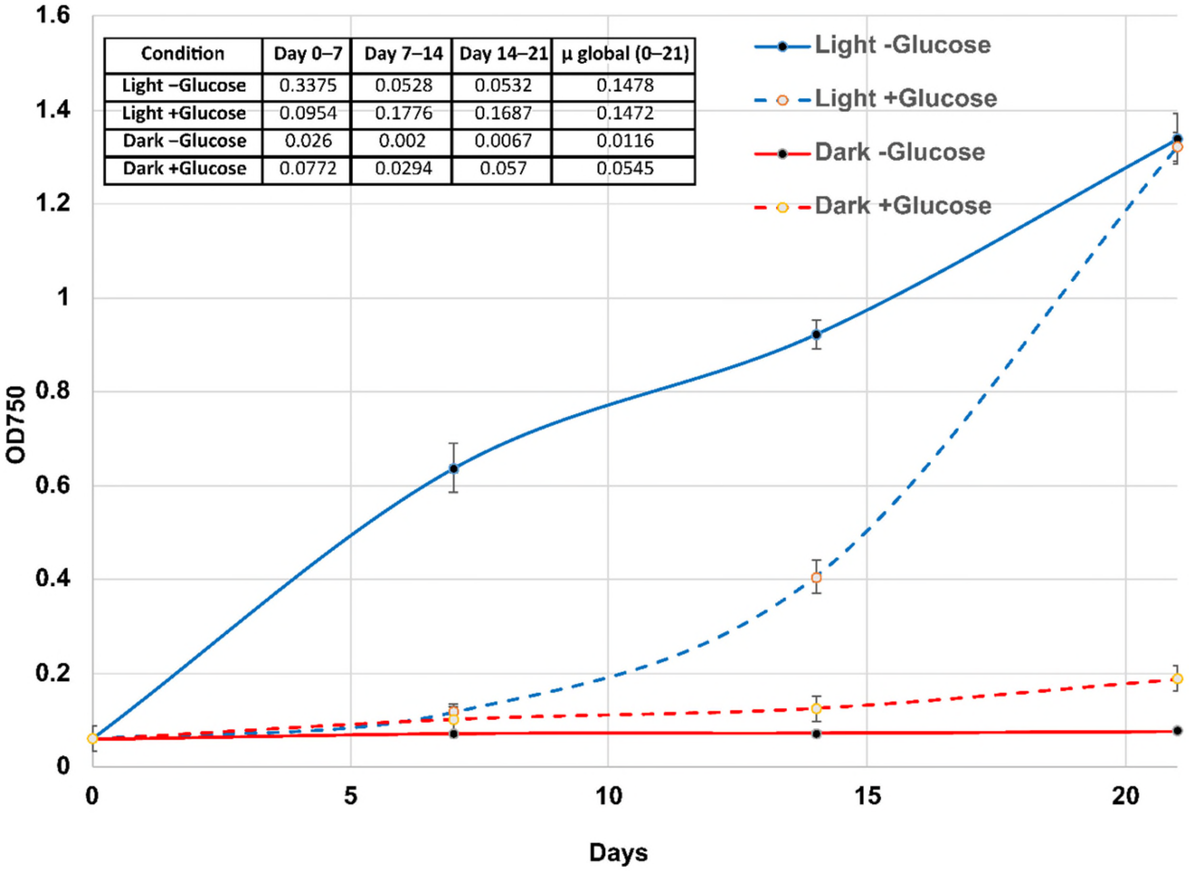

Condition	Day 0–7	Day 7–14	Day 14–21	μ global (0–21)
Light –Glucose	0.3375	0.0528	0.0532	0.1478
Light +Glucose	0.0954	0.1776	0.1687	0.1472
Dark –Glucose	0.026	0.002	0.0067	0.0116
Dark +Glucose	0.0772	0.0294	0.057	0.0545

Figure S1: 21-day growth curves of BSA11S strain under light/dark, +/- 0.1% glucose and oxic conditions. Net growth (μ) was calculated for 7-day intervals. Error bars based on standard deviation of triplicates are shown. Absorbance at 750 nm (expressed as OD750) was used as a proxy to monitor biomass.

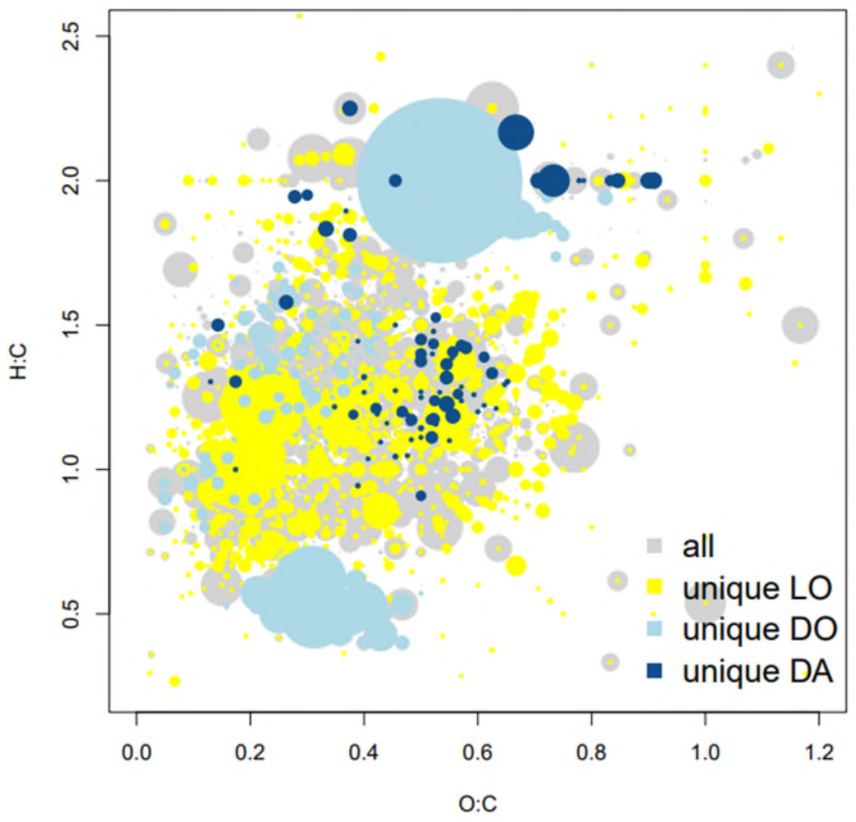

Figure S2: Van Krevelen diagram of all molecular formulas (gray) and molecular formulas unique to the dark anoxic (DA), dark oxic (DO), and light oxic (LO, control) treatments. Circle size is proportional to the molecular formula relative intensity averaged for replicates, with shared formula relative intensities × 10^3 and unique formula relative intensities ×10^4.

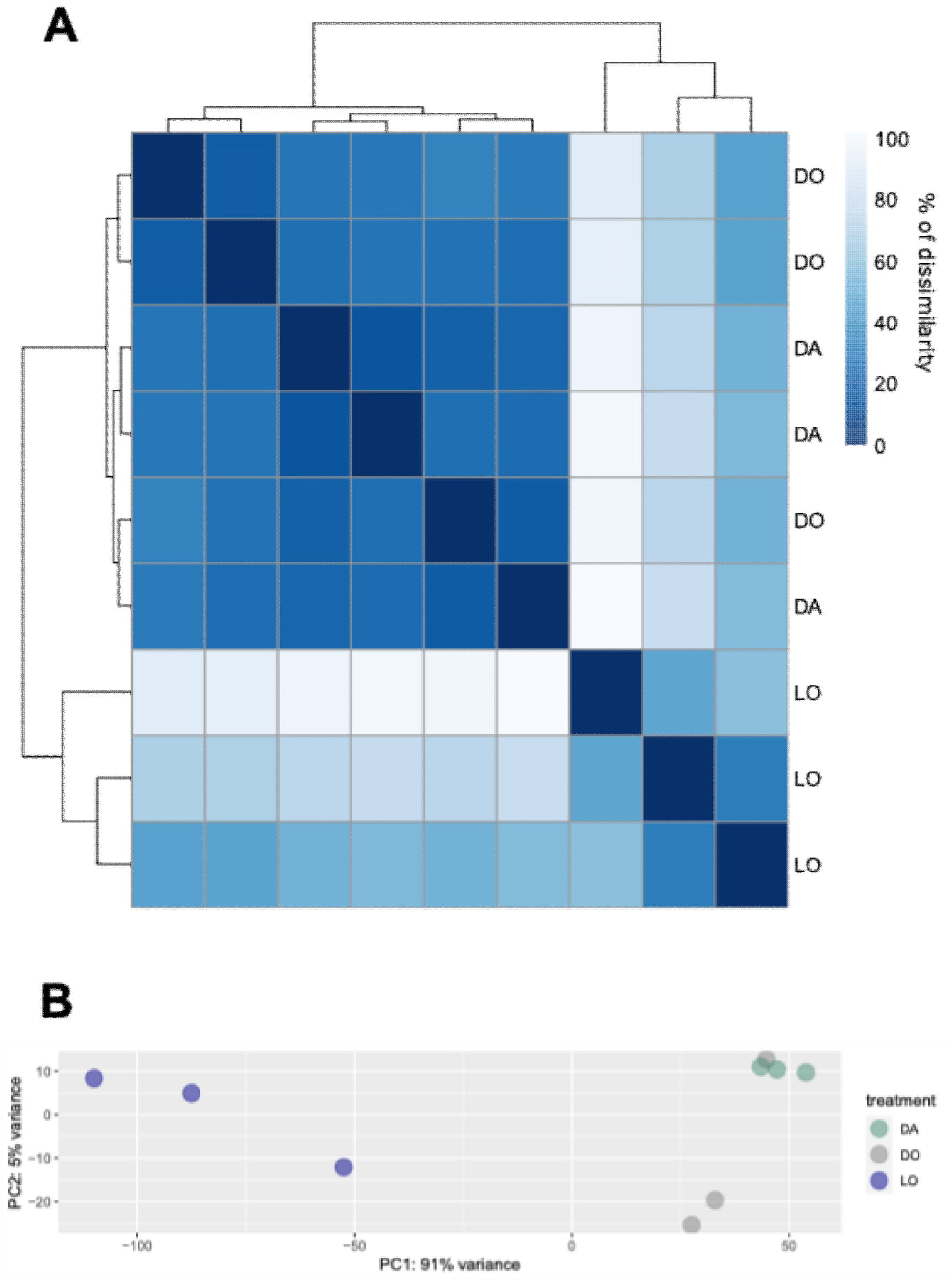

Figure S3. A) Heatmap of Bray-Curtis dissimilarities of transcriptome samples, ordered according to hierarchical clustering based on normalized gene abundance data (DeSeq2). B) PCA plot of triplicate LO, DA, and DO treatments.

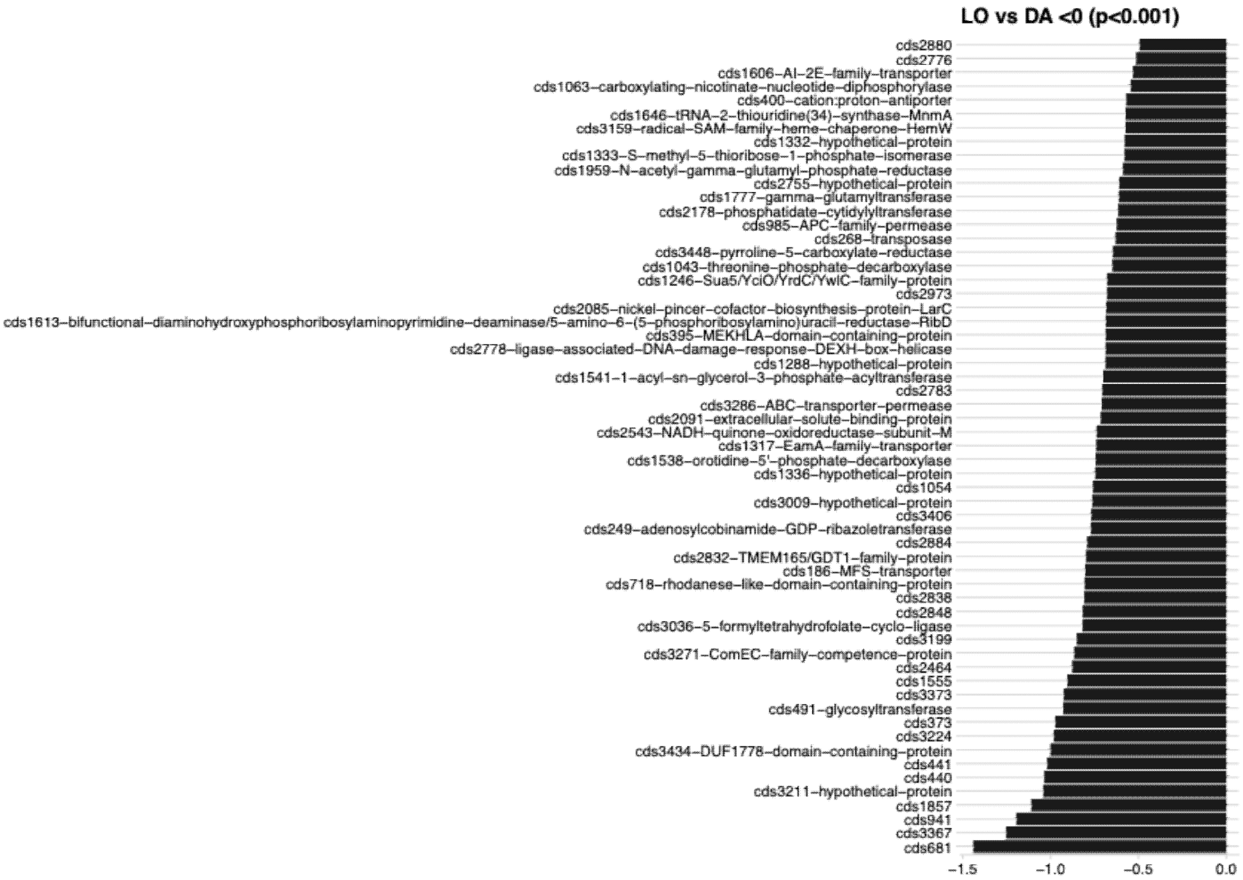

Figure S4. Top-60 significantly up-regulated genes for the DA vs. LO treatments, as determined with DeSeq2. X-axis indicates the log2Fold change between the two treatments.

Fig. S4 shows the top-60 up-regulated genes in DA vs LO. Amongst other genes overexpressed in DA, we found genes encoding transposases, DUF3427 domain-containing proteins, hypothetical proteins, tRNAs (for Val, Tyr, Cys, Pro, Asn, Ser, Arg, Leu, Phe or Glu), NAD/NADP transhydrogenase, bacterioferritin (involved in Fe storage), translesion-error-prone DNA polymerase V autoproteolytic subunit and orotidine 5'-phosphate decarboxylase (relevant for pyrimidine biosynthesis), as well as diverse transporter genes (i.e., Na+/H+-antiporter, cation efflux/transporters for Fe/Co/Zn/Cd and ABC-type dipeptide/oligopeptide/nickel transport systems, Fig. S3, Supplementary Dataset 2). Additional up-regulated genes under DA conditions (log2-fold change of between -0.5 to -1.5 in LO compared to DA conditions) included genes encoding fumarate hydratase, 5,10-methenyltetrahydrofolate synthetase, which might deplete folate to slow one-carbon biosynthetic metabolism. Pertinent to the S cycle, we observed an upregulation under DA conditions of the rhodanese, an alternative S cycle enzyme which converts thiosulfate and cyanide into sulfite and thiocyanate, a pathway that might be relevant in the sulfate-rich euxinic waters of the Black Sea. Notably, the NDH-I3 ChpY/CupA (CDS2542), which contributes to the high-affinity CO_2 transport in low-CO_2-inducible acquisition, was also upregulated in DA (see Supplementary dataset

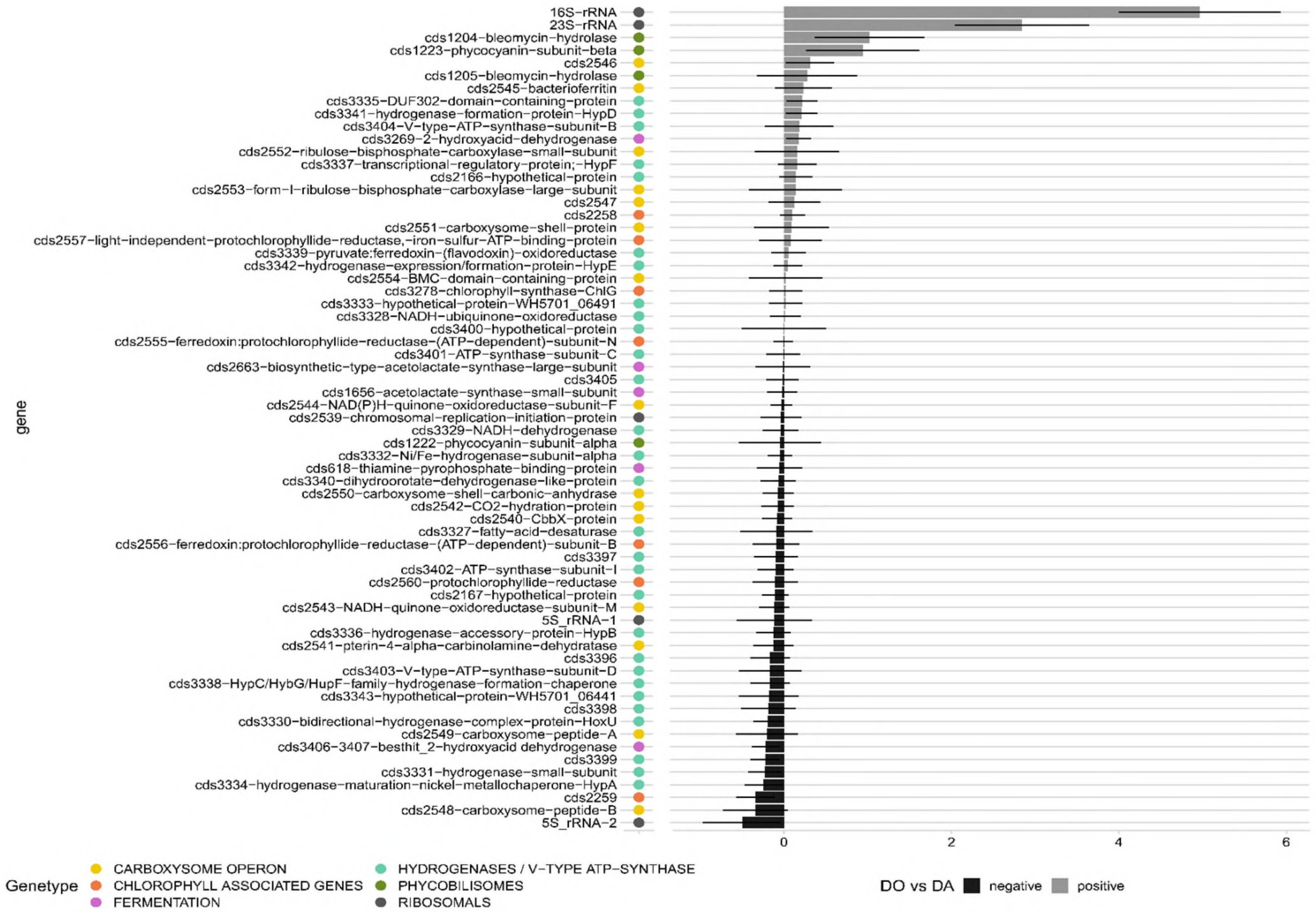

Figure S5. List of key genes relatively overexpressed in the DO treatment (positive values in log2Fold change up to 10 with p-values<0.01), and genes that are relatively overexpressed under DA conditions (negative values). Different genes are circle-colour coded according to different metabolic pathways or processes.

Table S1: Weighted molecular characteristics of the core dissolved organic matter population (DOM present in all samples) as well as characteristics of DOM molecular formulas (MFs) detected in all samples of one treatment (treatment_all) and molecular formulas MFs unique to each treatment (treatment_uni), including molecular mass, as well as elemental ratios, fraction of the carbohydrate-like compounds (%carbo), and nominal oxidation state of carbon atoms (NOSC). Standard deviation represents variability of replicate FT-ICR-MS analyses.

DOM	Molecular mass (Da)	H/C	O/C	N/C	S/C	P/C	% Carbo	NOSC
Core	371±5	1.33±0.011	0.42±0.20	0.007±0.002	0.002±0.000	0.0002±0.0000	0.13±0.11	-0.47±0.04
LO_all	367±0.6	1.31±0.00	0.43±0.00	0.150±0.000	0.004±0.000	0.0005±0.0000	0.17±0.02	-0.40±0.00
DO_all	372±0.0	1.35±0.00	0.40±0.00	0.005±0.000	0.003±0.000	0.0004±0.0000	0.11±0.01	-0.54±0.00
DA_all	379±0.2	1.32±0.00	0.44±0.00	0.008±0.000	0.003±0.000	0.0003±0.0000	0.47±0.02	-0.42±0.00
LO_uni	368±2	1.22±0.007	0.41±0.02	0.053±0.001	0.011±0.0015	0.0029±0.00070	0.11±0.00	-0.26±0.02
DO_uni	383±3	1.34±0.002	0.39±0.00	0.004±0.000	0.006±0.0002	0.0013±0.00025	0.03±0.00	-0.53±0.01
DA_uni	447±2	1.51±0.001	0.53±0.01	0.026±0.001	0.020±0.0006	0.0021±0.00001	0.14±0.02	-0.32±0.00

Table S2: Transcriptomics raw sequenced reads statistics for light oxic (LO), dark oxic (DO) and dark anoxic incubations (DA).

SAMPLE	SEQUENCED READS	TOTAL TRIMMED READS	AVERAGE READ LENGTH (bp)
57395_ID1683_1-L-O-1-T3_S1_L001_R1_001	35,391,541	28,043,279	140.1
57396_ID1683_2-L-O-2-T3_S1_L001_R1_001	29,481,209	17,027,840	113.7
57397_ID1683_3-L-O-3-T3_S1_L001_R1_001	49,491,952	41,955,718	138.2
57399_ID1683_7-D-O-1-T3_S1_L001_R1_001	42,289,529	40,491,573	124.1
57400_ID1683_8-D-O-2-T3_S1_L001_R1_001	19,216,119	15,229,038	133.6
57401_ID1683_9-D-O-3-T3_S1_L001_R1_001	22,961,139	17,586,075	125.9
57402_ID1683_10-D-A-4_S1_L001_R1_001	25,214,425	23,778,313	123.7
57403_ID1683_11-D-A-5_S1_L001_R1_001	29,280,056	27,640,827	114.4
57398_ID1683_5-D-A-2-T3_S1_L001_R1_001	30,286,534	25,739,174	112.1

Table S3. Molecular formulas, abundance (as relative intensity × 10^4) and nominal oxidation state of carbon of the carbohydrate-like compounds between different experimental treatments (light oxic, LO, dark oxic, DO and dark anoxic, DA).

Carbohydrate-like	Average rel. intensity			
**MF**	**LO**	**DO**	**DA**	**NOSC**
$C_9H_{18}O_8S$	2.53	1.34	10.23	0.00
$C_{11}H_{22}O_9S$	1.96	2.46	9.92	-0.18
$C_{13}H_{26}O_{10}S$	0.59	1.20	5.77	-0.31
$C_{10}H_{22}O_7S$	0.00	1.47	4.76	-0.60
$C_{15}H_{30}O_{11}S$	0.00	0.00	3.34	-0.40
$C_{11}H_{22}O_{10}S$	0.00	0.00	1.75	0.00
$C_{10}H_{20}O_9S$	0.00	0.00	1.69	0.00
$C_{17}H_{34}O_{12}S$	0.00	0.00	1.63	-0.47
$C_{13}H_{26}O_{11}S$	0.00	0.00	1.56	-0.15
$C_{11}H_{22}O_8S$	0.00	0.00	1.53	-0.36
$C_{10}H_{20}O_8S$	0.00	0.52	1.23	-0.20
$C_4H_6O_3$	0.49	1.79	1.19	0.00
$C_{12}H_{24}O_{10}S$	0.00	0.00	1.17	-0.17
$C_{11}H_{22}O8S_2$	0.00	0.00	0.59	-0.36
$C_9H_{18}O_7S$	0.00	0.00	0.59	-0.22
$C_{14}H_{28}O_{11}S$	0.00	0.00	0.53	-0.29
$C_5H_{12}O_4S$	0.47	0.00	0.00	-0.40
$C_5H_8O_4$	1.01	0.00	0.00	0.00
$C_6H_{10}O_6$	0.53	0.00	0.00	0.33
$C_7H_{14}O_6S$	1.83	0.00	0.00	0.29
$C_8H_{16}O_7S$	0.53	0.00	0.00	0.00
$C_9H_{18}O_9S$	1.25	0.00	0.00	0.22
$C_{10}H_{16}O_7$	0.55	0.00	0.00	0.00
$C_{10}H_{16}O_7S$	1.05	0.00	0.00	0.00
$C_{11}H_{20}O_8$	0.49	0.00	0.00	-0.36
$C_{12}H_{18}O_{10}S$	0.54	0.00	0.00	0.33
$C_{12}H_{22}O_9$	0.00	0.53	0.00	-0.33
$C_{14}H_{26}O_{10}$	0.00	2.12	0.00	-0.43
$C_{16}H_{26}O_{15}S$	0.61	0.00	0.00	0.38
$C_{16}H_{34}O_{12}S$	0.54	0.00	0.00	-0.50
$C_{18}H_{28}O_{16}$	1.14	0.00	0.00	0.22
$C_{22}H_{38}O_{17}$	0.62	0.00	0.00	-0.18

Supplementary dataset 1: BSA11S isolate genome CDS annotation with KO numbers, Nr, COG, TIGRFAMs and Cyanorak (CK) clusters.

Supplementary dataset 2: BSA11S CDS up and downregulation (log2FoldChange) in LO vs DA treatments.
